# A small molecule inhibits Ebola virus entry through glycoprotein stabilization

**DOI:** 10.1038/s41467-026-77332-3

**Published:** 2026-09-01

**Authors:** Fan Bu, Gang Ye, Kiran L. Sharma, Bruno La Rosa, Murugesan Palaniappan, Martin M. Matzuk, Damian W. Young, Hailey Turner-Hubbard, Kimberly Morsheimer, Callum Owen, Katalin Toth, Michael Cameron, Robert Davey, Srinivas Chamakuri, Peter Dosa, Fang Li

**Affiliations:** 1 Department of Pharmacology, University of Minnesota Medical School, Minneapolis, MN USA; 2 Center for Emerging Viruses, University of Minnesota, Minneapolis, MN USA; 3 Center for Drug Discovery, Department of Pathology and Immunology, Baylor College of Medicine, Houston, TX USA; 4 National Emerging Infectious Diseases Laboratories, Boston University, Boston, MA USA; 5 Department of Virology, Immunology, and Microbiology, Boston University School of Medicine, Boston, MA USA; 6 Verna and Marrs McLean Department of Biochemistry and Molecular Pharmacology, Baylor College of Medicine, Houston, TX USA; 7 Department of Chemistry, University of Florida Scripps Institute, Jupiter, FL USA; 8 Institute for Therapeutics Discovery and Development, Department of Medicinal Chemistry, College of Pharmacy, University of Minnesota, Minneapolis, MN USA

**Keywords:** Ebola virus, Cryoelectron microscopy, Drug screening, Medicinal chemistry

## Abstract

Ebola virus (EBOV) causes outbreaks with high fatality rates, yet potent small-molecule inhibitors targeting EBOV glycoprotein (GP) remain limited. Here, using DNA-encoded chemical libraries, we screen 4.73 billion compounds against EBOV GP and, through stereochemical optimization, identify MWAC-3634. This compound binds GP with high affinity (K_d_=40.7 nM), potently neutralizes infection (IC₅₀ = 0.65 nM), shows no detectable cytotoxicity, and exhibits favorable pharmacokinetics in mice. This potency substantially exceeds that of known small-molecule inhibitors targeting EBOV GP. Cryo-EM at 2.54 Å resolution reveals that MWAC-3634 occupies a hydrophobic cavity at the GP subunit interface and stabilizes GP, supporting an entry-blocking mechanism distinct from previously described GP-destabilizing inhibitors. In a lethal-challenge mouse model, MWAC-3634 administered either intraperitoneally or orally substantially improves survival and reduces clinical signs. These findings suggest that EBOV GP requires an optimal level of stability for entry, identify GP stabilization as an effective anti-filovirus strategy, and establish MWAC-3634 as a promising lead compound for development.

## Abstract (web summary)

Researchers identified MWAC-3634, a small molecule that blocks Ebola virus entry by stabilizing its surface glycoprotein, revealing a distinct anti-Ebola strategy. Oral or injected treatment improved survival and reduced disease signs in mice.

## Introduction

Ebola virus (EBOV) remains a major threat to global health and national security, owing to its high case-fatality rates and recurrent human outbreaks^1^. EBOV causes severe hemorrhagic fever, with an overall fatality of ~40%. It accounts for most documented human filovirus infections (33,820 cases to date), including the 2014–2016 West African epidemic (28,652 cases and 11,325 deaths)^2,3^. Outbreaks likely arise from both animal and human reservoirs: bats are a probable natural host^4^, and the virus can persist in survivors for years and later re-emerge^5^. Given EBOV’s long-term coexistence with humans and recurring spillovers, developing effective treatment strategies remains an urgent priority.

Current interventions against EBOV represent significant advances but also face limitations. FDA-approved vaccines and antibody therapies have improved prevention and treatment of EBOV disease^6–8^. However, these interventions require cold-chain transport and injection-based delivery, limiting their deployment in low-resource, remote, and warm-climate regions where EBOV outbreaks typically occur^9,10^. In addition to these approaches, investigational small-molecule antivirals targeting different steps of the EBOV infection cycle, particularly viral replication, have also been explored and have shown promise^11,12^. Such agents, however, act only after viral entry has occurred and typically require access to intracellular targets. In contrast, entry inhibitors can block infection at the earliest obligate step of the viral life cycle and may therefore provide an early line of defense, while also offering pharmacologic advantages by targeting an extracellular or endosomal process rather than a cytosolic target. These considerations highlight the need for orally available, potent small-molecule therapeutics that directly target EBOV entry.

The glycoprotein (GP) of EBOV mediates viral entry into host cells and is a primary target for therapeutic intervention^13^. GP is displayed on the viral surface as a homotrimer, consisting of three copies each of the receptor-binding subunit GP1 and the membrane-fusion subunit GP2^14^. GP1 harbors a receptor-binding site, which is shielded by a glycan cap and a mucin-like domain^13,15^. GP2 contains an internal fusion loop (IFL) and two heptad repeats (HR1 and HR2)^16^. During maturation, GP is cleaved at the GP1/GP2 boundary by the host protease furin, yet GP1 and GP2 remain associated in a metastable pre-fusion conformation. During cell entry, GP1 engages cell-surface factors to facilitate viral attachment, after which the virus is internalized by endocytosis^17^. In endosomes, cathepsins remove the glycan cap and mucin-like domain, exposing the receptor-binding site^18^. The exposed receptor-binding site then binds its host receptor Niemann-Pick C1 (NPC1) on the endosomal membrane^19,20^. Subsequently, GP2 undergoes a transition to its lowest-energy post-fusion state, in which HR1 and HR2 form a six-helix bundle that enables the IFL to merge the viral and endosomal membranes^21,22^. This conformational transition must be tightly regulated in both space and time, occurring only after NPC1 engagement and at the endosomal membrane. Together, these observations raise the possibility that perturbing the spatiotemporal regulation of GP conformational change could block EBOV entry.

Extensive efforts have been devoted to discovering small-molecule compounds targeting EBOV GP. These compounds have been identified from high-throughput chemical libraries using virus-infected cells (rather than directly targeting GP)^23–26^, virtual docking libraries^27^, or repurposed drug libraries^28–30^. Most reported compounds bind EBOV GP with K_d_ values in the millimolar range and block entry with IC₅₀ values in the micromolar range, with only a few approaching ~100 nM. Only a subset has been structurally characterized with experimentally defined GP binding sites^27,29–31^. Notably, all such compounds (including the well-known toremifene) occupy a cavity between GP1 and GP2, where they neutralize entry by destabilizing GP (Table S1)^27,29,30^. To our knowledge, no prior experimental high-throughput screen has directly used purified EBOV GP as the target, likely reflecting the difficulty of adapting purified GP-based binding assays to conventional high-throughput screening formats. Consequently, vast chemical spaces remain unexplored, contributing to the current lack of highly potent small-molecule inhibitors directly targeting EBOV GP.

DNA-Encoded Chemistry Technology (DEC-Tec) is an emerging platform for drug discovery that enables rapid, cost-effective screening of vast chemical libraries, eliminating the need for traditional high-throughput screening assays^32–36^. By tagging compounds with unique DNA barcodes, DEC-Tec allows direct identification of target binders through next-generation sequencing, bypassing the need for custom probes and complex detection systems. DNA-encoded chemical libraries typically contain far more compounds than conventional chemical libraries, providing much broader coverage of chemical space.

In this study, we identified a potent small-molecule inhibitor of EBOV entry that acts by stabilizing GP, revealing a distinct mechanism of neutralization. Using DNA-encoded chemical libraries, we screened 4.73 billion compounds against the EBOV GP ectodomain and, following optimization of an initial hit, developed MWAC-3634. MWAC-3634 exhibited favorable pharmacokinetics in mice and, as a proof of concept, improved survival and clinical disease metrics following either intraperitoneal or oral dosing in a lethal-challenge mouse model. Together, these results highlight DEC-Tec as a powerful tool for anti-filovirus drug discovery, identify GP stabilization as an anti-filovirus strategy, and establish MWAC-3634 as a potent and promising lead compound for further therapeutic development.

## Results

### Screening of DEC-Tec libraries for EBOV GP-binding compounds

**Fig. 1:**
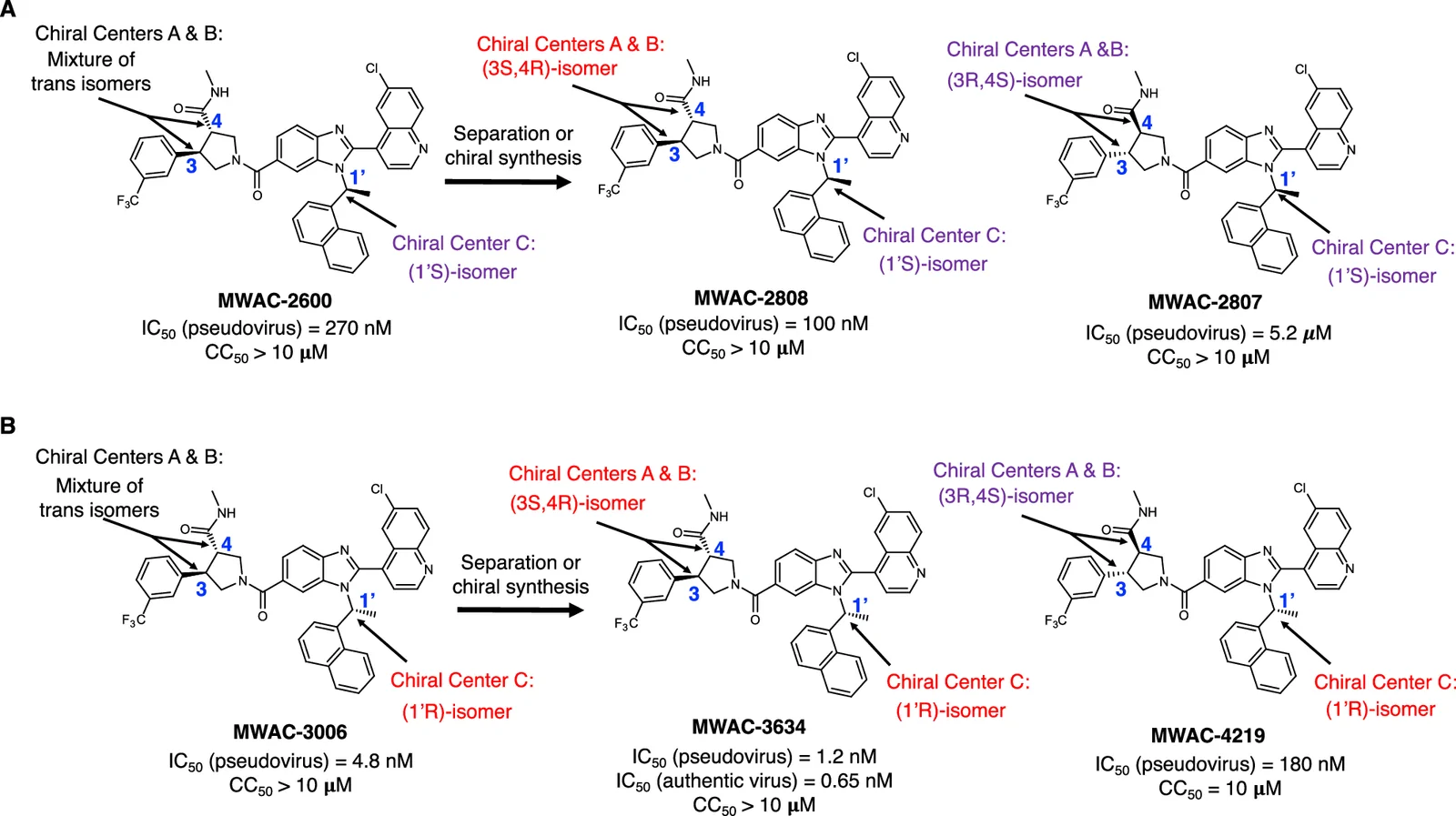
Stereochemical optimization of the lead EBOV GP-targeting compound identified through DEC-Tec screening. **A** MWAC-2600, the top hit compound identified from DEC-Tec screening, contains three chiral centers. The first round of stereochemical optimization involved separating two trans isomers at chiral centers (**A** and **B**). **B** The second round of optimization focused on altering the configuration at chiral center C from S to R. Chemical structures, chiral-center positions, antiviral potency, and cytotoxicity for MWAC-2600 and its derivative compounds are shown. Selected stereochemical configurations are labeled in red, alternative configurations are labeled in purple, and chiral-center positions are numbered in blue. IC₅₀, half-maximal inhibitory concentration; CC₅₀, half-maximal cytotoxic concentration.

To identify small-molecule inhibitors of EBOV entry, we used a recombinant His₆-tagged EBOV glycoprotein (GP) ectodomain previously employed in the discovery of GP-targeting nanobodies^37^. Using this protein as the target, we screened 4.73 billion unique compounds from the Baylor Center for Drug Discovery’s DNA-encoded chemical library collection^32–36^. Affinity selections were performed with 1 µM His₆-tagged EBOV GP ectodomain, alongside a control selection without the target protein to assess non-specific binding. Illumina next-generation sequencing revealed a consistently enriched chemical series exhibiting strong structure-enrichment relationships (Fig. S1). Based on these results, we synthesized two off-DNA candidate compounds: the quinoline derivative MWAC-2600 (Fig. 1A) and the triazole derivative MWAC-2601 (Fig. S2). Both compounds consist of three building blocks: BB1 (the fragment closest to the DNA), BB2, and BB3 (the fragment farthest from the DNA) (Fig. S1). For BB1, we opted for the CF₃-substituted phenyl-pyrrolidine unit over the dichlorophenyl-pyrrolidine moiety for both MWAC-2600 and MWAC-2601 because of its lower molecular weight.

Both MWAC-2600 and MWAC-2601 were evaluated using EBOV pseudovirus entry and cell toxicity assays. In the pseudovirus entry assay, retroviruses pseudotyped with EBOV GP (i.e., EBOV pseudoviruses) were used to infect human cells in the presence of varying concentrations of each compound. The neutralizing potency (IC₅₀) was defined as the concentration required to inhibit pseudovirus entry by 50%. In the cytotoxicity assay, compounds were incubated with cells at various concentrations, and cytotoxicity (CC₅₀) was defined as the concentration causing 50% cell death. MWAC-2600 inhibited EBOV pseudovirus entry with an IC₅₀ of 270 nM and exhibited minimal cytotoxicity under assay conditions, with a CC₅₀ greater than 10 µM (Figs. 1A and S3A). In contrast, MWAC-2601 was a weak inhibitor, with an IC₅₀ of 51 µM, although it also showed low cytotoxicity (CC₅₀ > 10 µM) (Figs. S2 and S3B). These results suggest that MWAC-2600 is the more promising EBOV entry inhibitor, demonstrating both potency and safety.

### Stereochemical optimization and evaluation of top EBOV GP-binding compound

MWAC-2600 contains three chiral centers: centers A and B on the pyrrolidine ring (positions 3 and 4), which exist as a mixture of trans isomers, and center C adjacent to the naphthyl group (position 1′), which is fixed in the (S)-configuration. Chiral HPLC was used to separate the diastereomeric mixture at centers A and B, yielding two distinct compounds: the (3S,4R)-isomer, designated MWAC-2808, and the (3R,4S)-isomer, designated MWAC-2807 (Fig. 1A). The stereochemistry of MWAC-2808 was confirmed via de novo diastereoselective synthesis (see Protocol S1 in the Supplementary Information)^38^. MWAC-2808 exhibited higher potency than MWAC-2600 (IC₅₀ against pseudoviruses = 100 nM) (Fig. S3C), whereas MWAC-2807 was markedly less potent (IC₅₀ against pseudoviruses = 5.2 µM) (Fig. S3D). Both compounds showed low cytotoxicity under the assay conditions (Figs. S4B and S4C). These findings establish the (3S,4R) configuration at centers A and B as optimal for activity in this compound series.

**Fig. 2:**
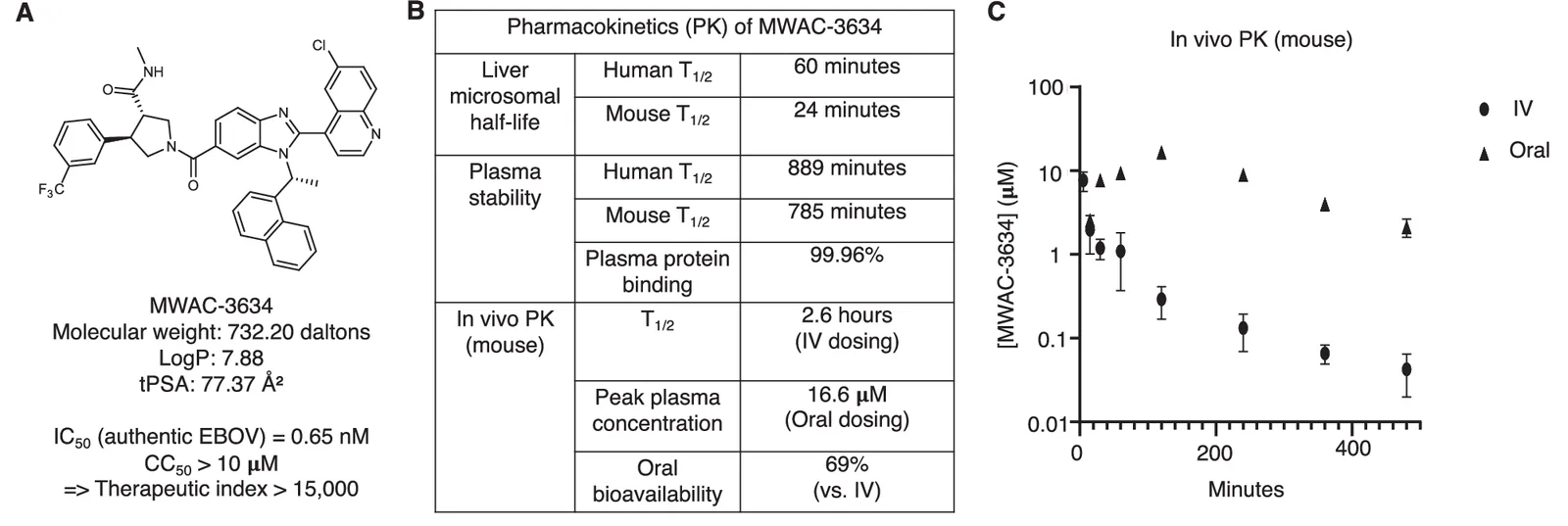
Pharmacokinetic properties of MWAC-3634, the lead compound following stereochemical optimization. **A** Overview of MWAC-3634, including its chemical structure, molecular weight, LogP (octanol-water partition coefficient), topological polar surface area (tPSA), anti-EBOV potency, cytotoxicity, and calculated therapeutic indices. **B** Summary of MWAC-3634’s pharmacokinetic properties, including liver microsomal half-life, plasma stability, and in vivo pharmacokinetics. **C** Oral bioavailability of MWAC-3634 compared with intravenous (IV) administration in mice. Circles and triangles indicate IV and oral administration, respectively. Data are presented as mean ± SD (*n* = 3 mice per administration route); each mouse represented one biological replicate, and serial blood samples were collected from the same mice at the indicated time points. PK pharmacokinetics, T₁/₂ half-life, IC₅₀ half-maximal inhibitory concentration, CC₅₀ half-maximal cytotoxic concentration, SD standard deviation. Source data are provided as a Source Data file.

We next examined the effect of modifying the third chiral center of MWAC-2600 by switching the configuration at center C from (S) to (R). The resulting compound, MWAC-3006 (Fig. 1B), potently neutralized EBOV pseudovirus entry with an IC₅₀ of 4.8 nM and exhibited no cytotoxicity under assay conditions (CC₅₀ > 10 µM) (Figs. S3E and S4D). Like MWAC-2600, MWAC-3006 consisted of a mixture of (3S,4R)- and (3R,4S)-diastereomers. To identify the more active isomer, both diastereomers were synthesized and evaluated individually. The (3S,4R)-diastereomer proved more potent and was designated MWAC-3634. This compound neutralized pseudovirus entry mediated by GP from the prototypic EBOV strain Mayinga with an IC₅₀ of 1.2 nM (Fig. S5A). It also inhibited authentic wild-type EBOV Mayinga infection with an IC₅₀ of 0.65 nM (Fig. S5B). In contrast, the (3R,4S)-diastereomer, MWAC-4219, inhibited EBOV pseudovirus entry with a much weaker IC₅₀ of 180 nM (Fig. S3F), although it also showed low cytotoxicity under assay conditions (CC₅₀ = 10 µM) (Fig. S4E). Notably, MWAC-3634 exhibited no cytotoxicity under assay conditions (CC₅₀ > 10 µM) (Fig. S4F), corresponding to a therapeutic index of >15,000 for cytotoxicity (Fig. 2A). To assess whether MWAC-3634 retains activity across EBOV strains, we tested it against pseudoviruses bearing GP from three additional EBOV strains. MWAC-3634 neutralized pseudovirus entry with IC₅₀ values of 0.83, 0.63, and 1.8 nM against the Ituri, Mbandaka, and Makona strains, respectively (Fig. S5A). To assess potential cross-filovirus activity, we next evaluated MWAC-3634 against SUDV and MARV pseudoviruses. In addition to EBOV, MWAC-3634 inhibited entry of both SUDV and MARV pseudoviruses, with IC₅₀ values of 2.62 and 2.05 μM, respectively (Fig. S6). Thus, MWAC-3634 shows strong activity across tested EBOV strains but only modest cross-filovirus activity. In summary, through stereochemical optimization, MWAC-3634 emerged as the most potent EBOV-directed derivative and was selected for further characterization.

### Pharmacokinetic properties of top EBOV GP-binding compound

We evaluated the in vitro pharmacokinetic properties of MWAC-3634. The compound exhibited moderate stability in human liver microsomes, with a half-life (T₁/₂) of 60 min, and lower stability in mouse liver microsomes (T₁/₂ = 24 min) (Figs. 2B and S7A, B). It showed low aqueous solubility (0.08 µM). Notably, this solubility remains approximately 123-fold higher than its anti-EBOV IC₅₀ (0.65 nM), indicating that the compound can achieve concentrations within a range relevant for antiviral activity. MWAC-3634 also displayed high plasma protein binding (99.96%). The compound was also highly stable in plasma, with half-lives of 889 min in human plasma and 785 min in mouse plasma (Figs. 2B and S7C, D). Overall, while improvements in solubility and microsomal stability in mice may be necessary, MWAC-3634 exhibits favorable plasma stability.

We also evaluated the in vivo pharmacokinetic properties of MWAC-3634. Mice were dosed either orally (50 mg/kg, *n* = 3) or intravenously (2 mg/kg, *n* = 3) (Fig. 2B, C). All animals remained healthy following dosing. After IV administration, the half-life was 2.6 h. The oral bioavailability (%F) of MWAC-3634 was 69%. Following oral administration, plasma concentrations remained above 2 µM for eight hours. These encouraging results suggest that MWAC-3634, or an optimized derivative, is likely to achieve sufficient exposure to support in vivo antiviral evaluation.

### Functional characterization of top EBOV GP-binding compound

**Fig. 3:**
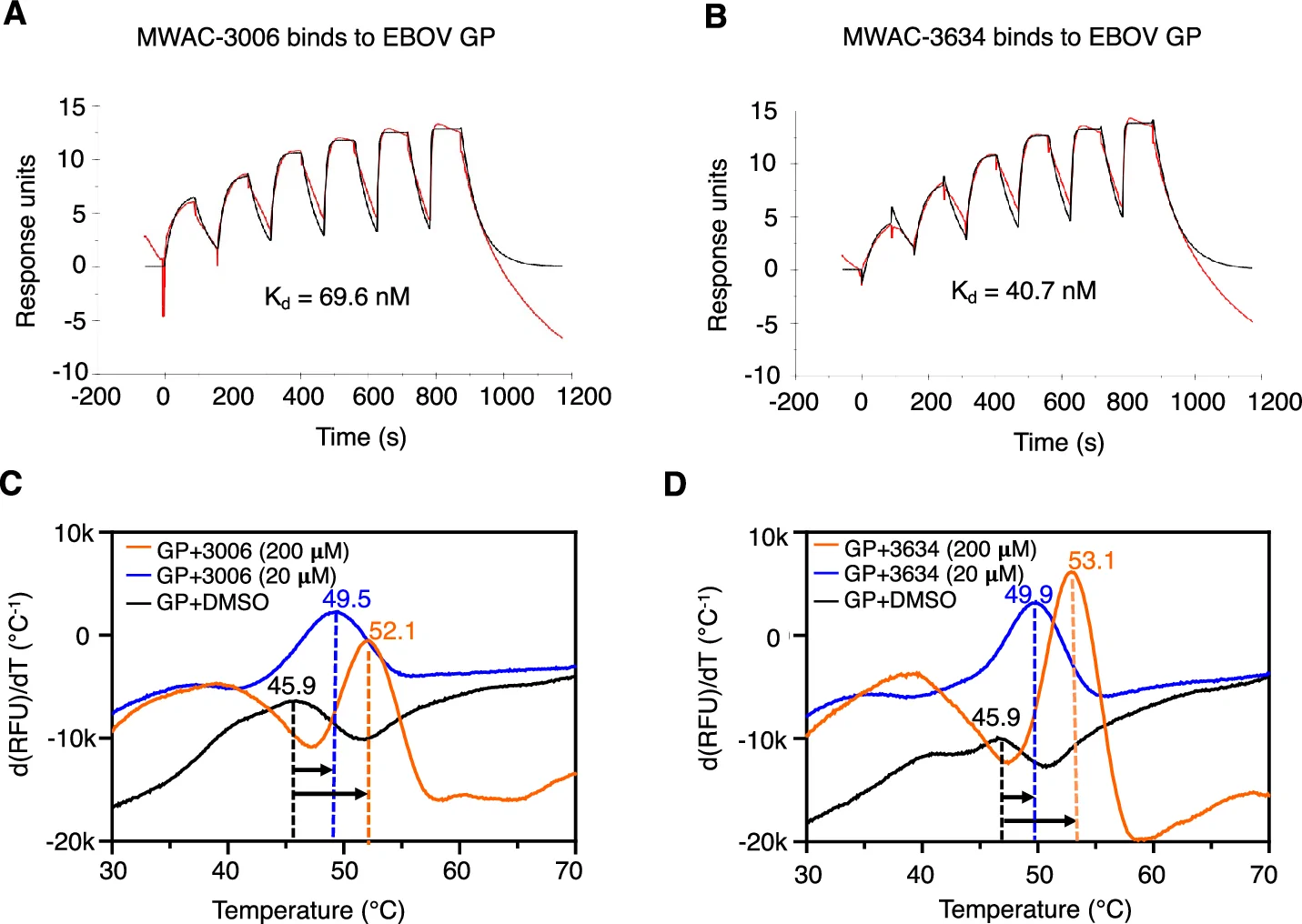
Biochemical mechanisms of EBOV GP-targeting compounds. **A**, **B** Binding affinities between EBOV GP-targeting compounds and recombinant EBOV GP ectodomain were determined by surface plasmon resonance (SPR). Red curves represent experimental sensorgrams, and black curves represent fitted binding curves. **C**, **D** Differential scanning fluorimetry assays were used to assess the effects of EBOV GP-targeting compounds on the thermal stability of the recombinant EBOV GP ectodomain. Orange and blue curves represent compound concentrations of 200 and 20 µM, respectively, and black curves represent the DMSO control. Dashed vertical lines indicate peak temperatures, and black arrows indicate thermal shifts relative to the DMSO control. Kd equilibrium dissociation constant, RFU relative fluorescence units, DMSO dimethyl sulfoxide.

To evaluate the target-binding affinity of MWAC-3634, we performed surface plasmon resonance (SPR) analysis using recombinant EBOV GP ectodomain. The EBOV GP ectodomain was immobilized on SPR chips, and varying concentrations of MWAC-3634 were injected while SPR signals were recorded. MWAC-3006, a mixture of two trans-isomers at chiral centers A and B (MWAC-3634 and MWAC-4219), was used for comparison. Our data showed that MWAC-3006 bound to EBOV GP with a dissociation constant (K_d_) of 69.6 nM (Fig. 3A), whereas MWAC-3634 bound with a K_d_ of 40.7 nM (Fig. 3B). These results indicate a modest improvement in binding affinity following stereochemical optimization and establish MWAC-3634 as a high-affinity EBOV GP binder.

To investigate the biochemical mechanism by which MWAC-3634 neutralizes EBOV entry, we examined its effect on the thermostability of the EBOV GP ectodomain by measuring compound-induced thermal shifts. Two concentrations of MWAC-3634 were tested, with MWAC-3006 again used for comparison. Our data showed that at 20 and 200 µM, MWAC-3006 increased the thermostability of EBOV GP ectodomain by 3.6 and 6.2 °C, respectively (Fig. 3C). MWAC-3634, the more potent component of MWAC-3006, produced larger thermal shifts of 4.0 °C (20 µM) and 7.2 °C (200 µM), respectively (Fig. 3D). Similar to the SPR results, these data further support the success of stereochemical optimization and reinforce MWAC-3634 as a strong EBOV GP binder that stabilizes the prefusion state of GP.

### Structural mechanisms of top EBOV GP-binding compound

**Fig. 4:**
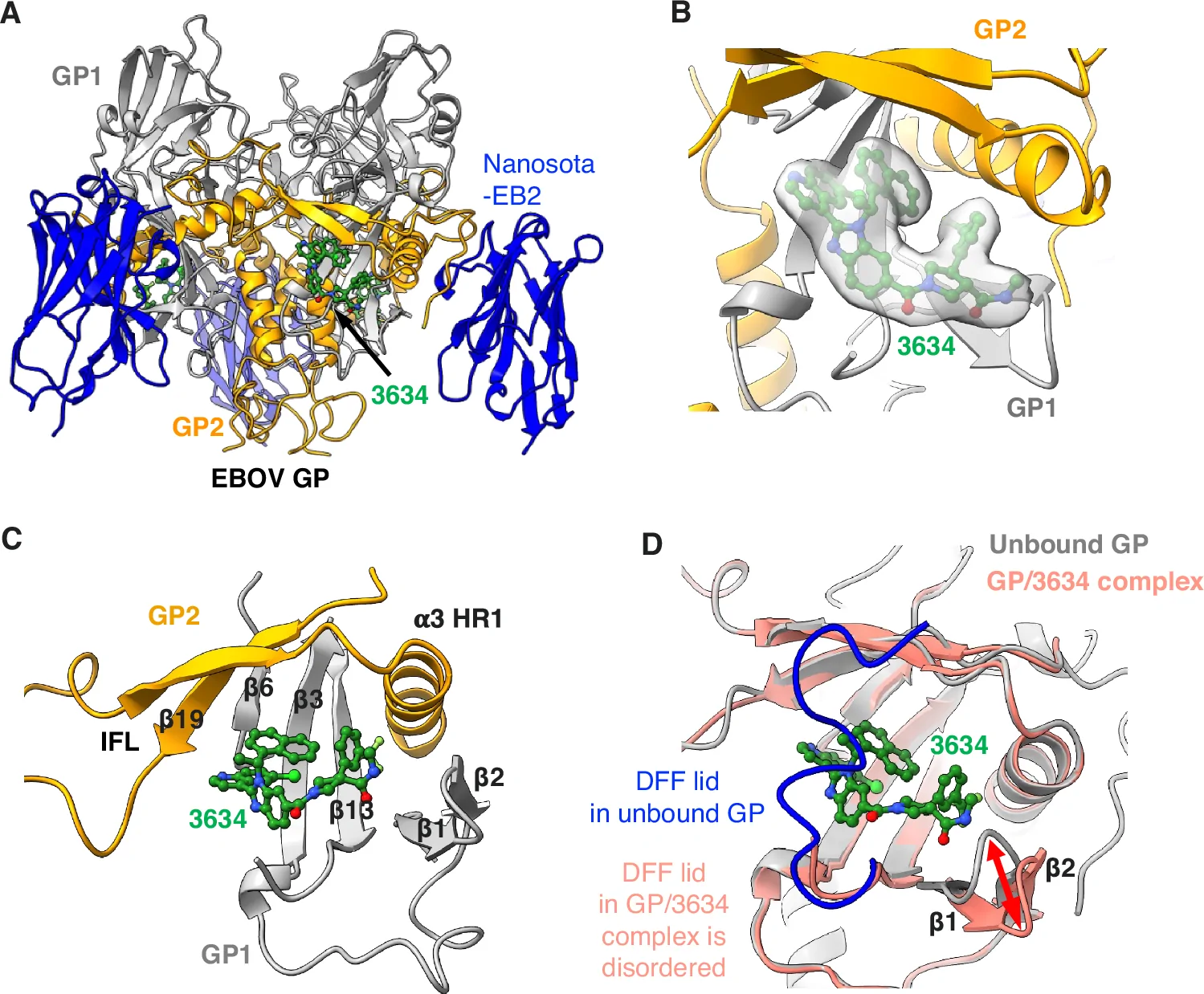
Structure of EBOV GP bound by MWAC-3634. **A** Cryo-EM structure of the ternary complex of the EBOV GP ectodomain, MWAC-3634, and Nanosota-EB2 (a GP-targeting nanobody used to enhance structural resolution), resolved at 2.54 Å. The GP1 and GP2 subunits of the EBOV GP ectodomain are shown in grey and orange, respectively; MWAC-3634 is shown in green; Nanosota-EB2 is shown in blue. **B** Cryo-EM density map of MWAC-3634 with its corresponding atomic model fitted. **C** Structural elements from both GP1 and GP2 that interact with MWAC-3634. **D** Structural changes in the EBOV GP ectodomain induced by MWAC-3634 binding. The structure of the unbound EBOV GP ectodomain (in grey, PDB: 9BSV) is superimposed with the MWAC-3634-bound structure (in pink). The DFF lid (blue in the apo structure) is displaced and becomes disordered upon MWAC-3634 binding. Additionally, the β1–β2 loop is pushed outward in the MWAC-3634-bound structure. The red arrow indicates outward displacement of the β1–β2 loop. Cryo-EM cryogenic electron microscopy, IFL internal fusion loop, HR1 heptad repeat 1, DFF Asp-Phe-Phe, PDB Protein Data Bank.

To investigate the structural basis of target binding by MWAC-3006, we purified the complex of recombinant EBOV GP ectodomain with MWAC-3006 and attempted to determine its cryo-EM structure. Previous EBOV GP-compound structures were determined by crystallography, where crystal packing and/or crystallization conditions may help stabilize GP and facilitate ligand density visualization. However, the GP-MWAC-3006 complex showed preferred particle orientation on cryo-EM grids, which limited the quality of the reconstruction^27,29–31^. To overcome this limitation, we purified a ternary complex comprising recombinant EBOV GP ectodomain, MWAC-3006, and Nanosota-EB2, a nanobody that binds the GP2 subunit of EBOV GP^37,39^. The addition of the nanobody improved particle orientation distribution and increased the effective molecular mass of the complex, thereby facilitating higher-quality cryo-EM reconstruction. As a result, the cryo-EM structure of the ternary complex was determined at 2.54 Å resolution (Fig. S8 and Table S2). In this structure, the density corresponding to the GP-bound compound was well-resolved and identified as MWAC-3634 (Fig. 4A, B). This is because MWAC-3634, the most potent target-binding component of MWAC-3006, became enriched during sample preparation and structural determination. These structural findings further confirm MWAC-3634 as the active component of MWAC-3006.

The cryo-EM structure revealed the binding site of MWAC-3634 within EBOV GP. In the apo structure of EBOV GP in the prefusion state, a large hydrophobic pocket is formed at the GP1/GP2 interface of each GP protomer, involving β1, β2, β3, β6, and β13 of GP1, as well as α3 (part of HR1), β19 and β20 (part of the IFL) of GP2 (Fig. 4C). This pocket is occupied and sealed by the DFF lid, named for its component residues Asp192 (D), Phe193 (F), and Phe194 (F); this lid stabilizes the GP structure (Fig. 4D)^40^. In the complex structure of EBOV GP bound to MWAC-3634, the compound occupies this hydrophobic pocket, interacting with β1, β2, β3, β6, and β13 of GP1 and α3 (part of HR1) and β19 (IFL) of GP2 (Fig. 4C). Compared to the apo structure, two major conformational changes were observed upon MWAC-3634 binding. First, the DFF lid, which occupies the pocket in the apo structure and would otherwise create a steric clash with MWAC-3634, becomes disordered, consistent with displacement of this lid upon MWAC-3634 binding (Fig. 4D). Second, the loop connecting β1 and β2 (i.e., the β1-β2 loop), which would also sterically conflict with MWAC-3634, is pushed away from the binding pocket (Fig. 4D). Thus, MWAC-3634 binding to the GP1/GP2 interface pocket induces significant conformational changes in EBOV GP.

**Fig. 5:**
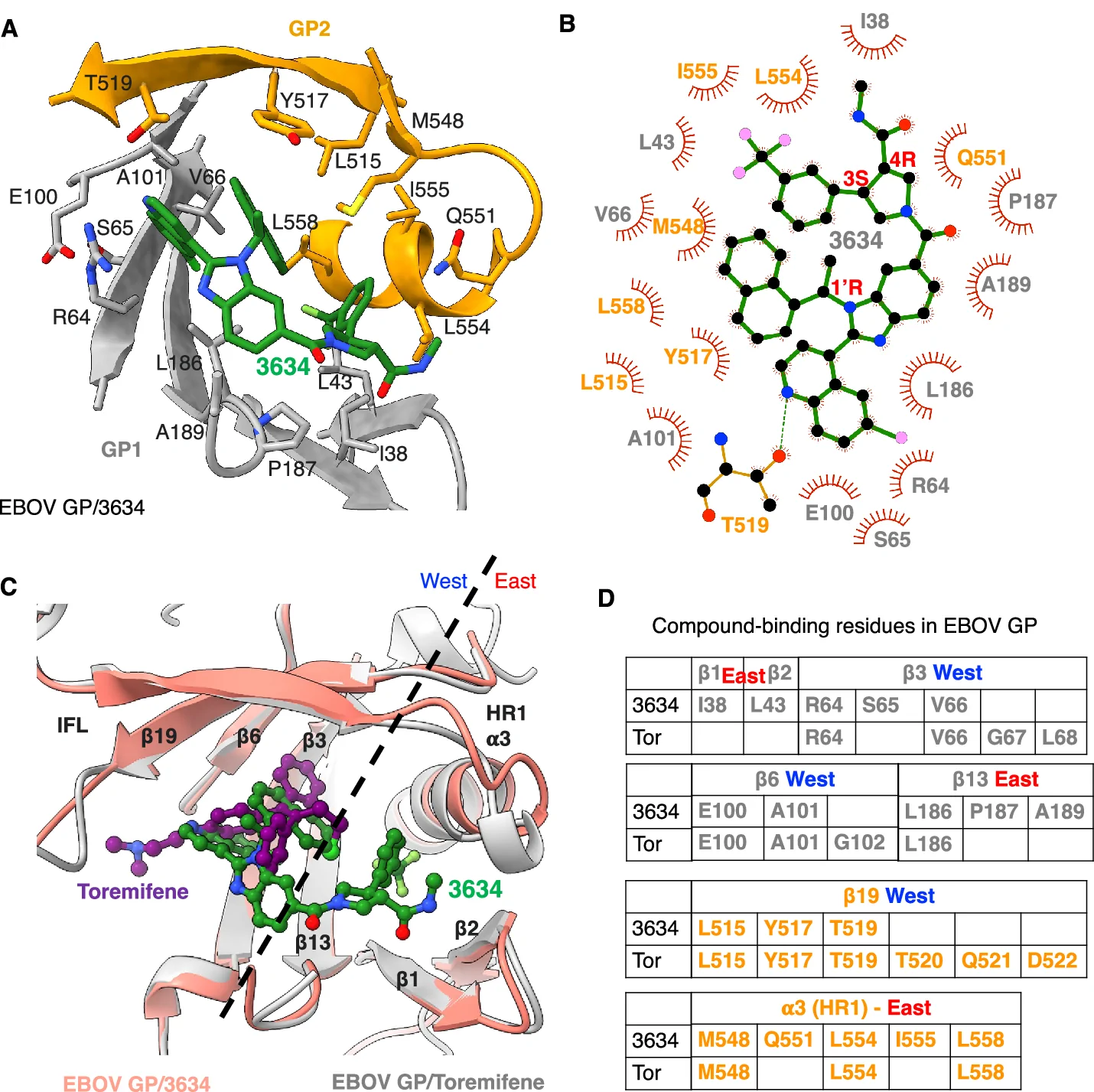
Detailed structural interactions between EBOV GP and MWAC-3634. **A** Residues on EBOV GP that directly interact with MWAC-3634. GP1 and GP2 residues are shown in grey and orange, respectively. **B** Detailed interactions between GP residues and MWAC-3634. Thr519 forms a hydrogen bond with MWAC-3634 (dashed line), Arg64 forms a cation–π interaction, and the remaining interactions are hydrophobic (indicated by spike arcs). **C** Comparison of GP-binding modes of MWAC-3634 (green) and toremifene (purple). The MWAC-3634-bound GP ectodomain (pink) is superimposed with the toremifene-bound GP ectodomain (grey; PDB: 5JQ7). The GP structure can be divided into western and eastern regions; MWAC-3634 interacts with both regions, whereas toremifene binds predominantly to the western region. **D** Comparison of GP residues involved in binding MWAC-3634 and/or toremifene. In (**C**), western and eastern regions are labeled in blue and red, respectively. Tor toremifene, IFL internal fusion loop, HR1 heptad repeat 1, PDB Protein Data Bank.

The cryo-EM structure also revealed detailed interactions between MWAC-3634 and EBOV GP. A total of 18 GP residues are directly involved in binding MWAC-3634 (Fig. 5A, B). Of these, only five are polar residues: Ser65, Arg64, Glu100, Thr519, and Gln551. Among the polar residues, Thr519 forms a hydrogen bond with MWAC-3634, while Arg64 engages in a cation–π interaction. The remaining three polar residues, along with all 13 nonpolar residues, contribute to hydrophobic interactions. These findings are consistent with the hydrophobic nature of the GP1/GP2 interface pocket and the interactions between GP and MWAC-3634. Structurally, MWAC-3634 folds into two segments, as oriented in Fig. 5C: the western portion interacts with eight residues from β3 and β6 of GP1 and β19 of GP2, while the eastern portion engages ten residues from β1, β2, and β13 of GP1, as well as α3 (HR1) of GP2 (Fig. 5C, D). These extensive contacts explain the strong binding affinity between MWAC-3634 and GP. Furthermore, the structure highlights the critical role of the optimized configurations of the three chiral centers. Any change in these chiral configurations would likely disrupt key GP-compound interactions (Fig. 5A, B), thereby weakening the compound’s binding affinity. Overall, this detailed structural insight explains the biochemical findings.

To date, all structurally characterized small-molecule compounds that target EBOV GP, including the repurposed cancer drug toremifene, bind to the same GP1/GP2 interface pocket (Fig. 5C, D and Table S1)^27,29,30^. However, these compounds primarily engage the western portion of the pocket. For example, among the 17 GP residues that interact directly with toremifene, 13 are located in the western region and only 4 in the eastern region. In contrast, MWAC-3634 interacts with 8 residues from the western portion and 10 from the eastern portion. Therefore, compared to previously reported GP-targeting compounds, MWAC-3634 forms more extensive contacts across multiple structural elements of GP, consistent with the SPR and thermostability data showing high-affinity GP binding and stabilization of the prefusion GP state. Together, these biochemical and structural findings support a model in which MWAC-3634 blocks EBOV entry by stabilizing prefusion GP, thereby increasing the energy barrier for transition to the post-fusion conformation.

### Proof-of-concept in vivo potency of MWAC-3634 in a lethal-challenge mouse model

To evaluate the proof-of-concept in vivo potency of MWAC-3634, we used a mouse model of lethal EBOV infection. Because wild-type (WT) EBOV does not infect wild-type mice, we assessed the in vitro neutralization potency of MWAC-3634 against a mouse-adapted (MA) EBOV bearing the S246P GP substitution and eight additional genomic substitutions. MWAC-3634 showed reduced potency against MA (IC_50_ = 2.87 nM) relative to WT (IC_50_ = 0.65 nM) (Fig. S5B). Nevertheless, MA EBOV provides a practical strain for in vivo evaluation despite its reduced neutralization sensitivity relative to WT.

**Fig. 6:**
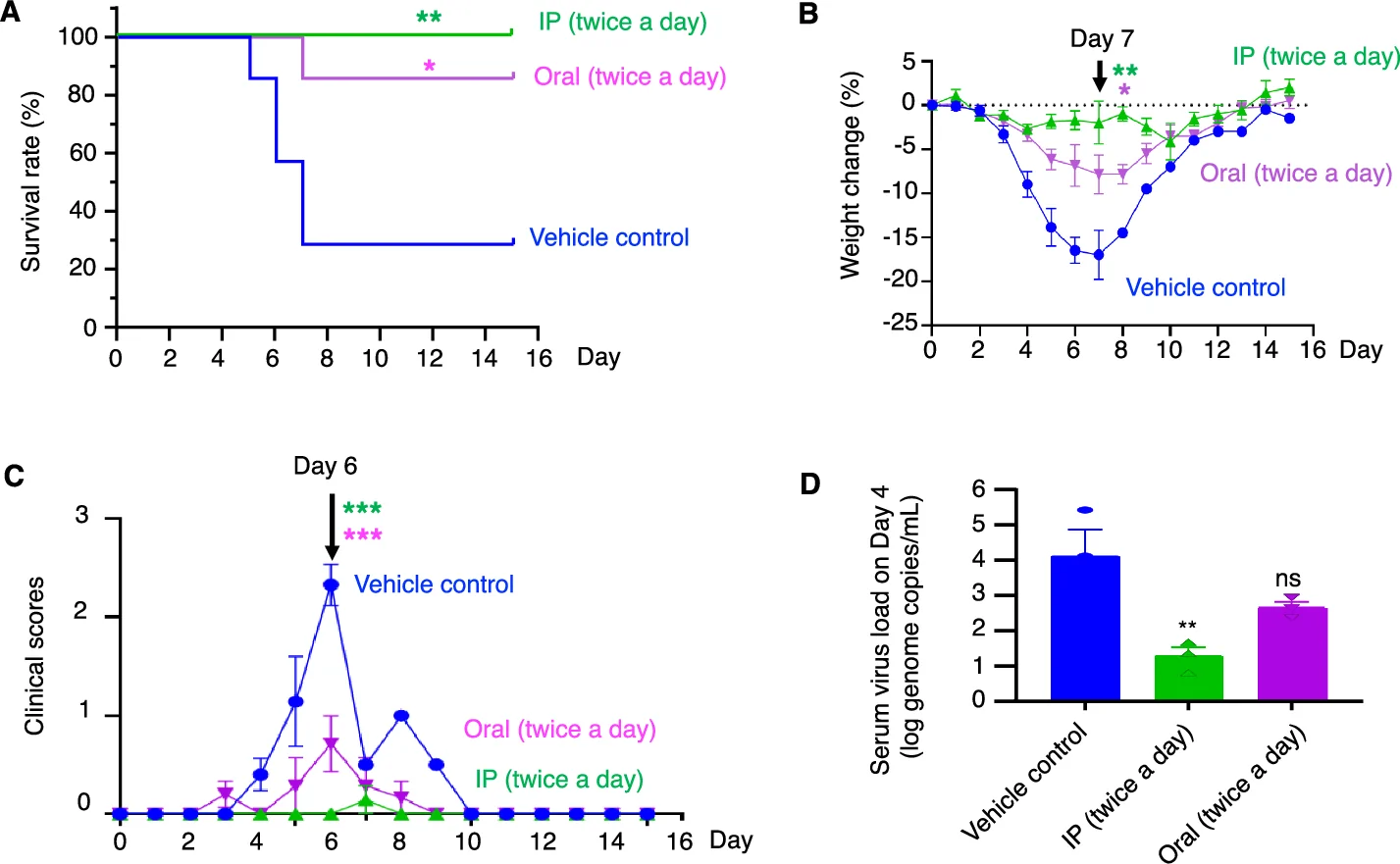
Proof-of-concept antiviral potency of MWAC-3634 in a lethal-challenge mouse model of EBOV infection. **A–C** Groups of 10 mice were challenged with mouse-adapted (MA) EBOV (see Fig. S5B) and treated twice daily with MWAC-3634 by intraperitoneal (IP) injection or oral administration, or with vehicle alone. On day 4, three mice per group were used for serum collection and removed from the study; the remaining seven mice were monitored through day 16. Each mouse represented one independent biological replicate. Vehicle control, IP treatment, and oral treatment are shown in blue, green, and magenta, respectively; circles, upward triangles, and downward triangles identify these groups where symbols are shown. **A** Survival curves were generated using the Kaplan–Meier method, and each treatment group was compared with the vehicle control using the Mantel–Cox log-rank test (*n* = 7 mice per group). For IP treatment versus vehicle, *χ*²(1) = 7.24, *p* = 0.0071, risk difference (RD) = 71.4 percentage points, 95% CI = [21.2, 91.8]. For oral treatment versus vehicle, *χ*²(1) = 4.90, *p* = 0.0268, RD = 57.1 percentage points, 95% CI = [5.8, 80.6]. **B** Body weight was recorded daily for each animal available at the time of measurement, and percentage change was calculated relative to that animal’s baseline. Animals that died on a given day were included in the body-weight data for that day. Data are presented as mean ± SEM when *n* ≥ 3; error bars are not shown when *n* < 3. Day 7 body-weight data were analyzed by one-way ANOVA, comparing each treatment group with the vehicle control (vehicle, *n* = 4; IP, *n* = 7; oral, *n* = 7). For IP treatment versus vehicle, *F*(1, 15) = 15.77, *p* = 0.0012, mean difference (MD) = 15.00 percentage points, 95% CI = [6.95, 23.05]. For oral treatment versus vehicle, *F*(1, 15) = 5.86, *p* = 0.0287, MD = 9.14 percentage points, 95% CI = [1.09, 17.19]. **C** Clinical scores were recorded daily only for animals alive at the time of assessment. Data are presented as mean ± SEM when *n* ≥ 3; error bars are not shown when *n* < 3. Day 6 clinical-score data were analyzed by one-way ANOVA, comparing each treatment group with the vehicle control (vehicle, *n* = 6; IP, *n* = 7; oral, *n* = 7). For IP treatment versus vehicle, *F*(1, 17) = 62.80, *p* = 4.15 × 10⁻⁷, MD = −2.33 clinical-score units, 95% CI = [−2.95, −1.71]. For oral treatment versus vehicle, *F*(1, 17) = 30.23, *p* = 3.92 × 10⁻⁵, MD = −1.62 clinical-score units, 95% CI = [−2.24, −1.00]. **D** Serum viral load was measured on day 4 in three mice per group. Genome copies per milliliter were back-calculated from quantification-cycle values using a standard curve of synthetic RNA corresponding to the amplicon. Data are presented as mean ± SEM. Viral-load values were log₁₀-transformed and analyzed by one-way ANOVA, comparing each treatment group with the vehicle control (*n* = 3 mice per group). For IP treatment versus vehicle, *F*(1, 6) = 16.36, *p* = 0.0068, MD = −3.14 log₁₀ genome copies/mL, 95% CI = [−5.04, −1.24]. For oral treatment versus vehicle, *F*(1, 6) = 3.44, *p* = 0.1131, MD = −1.44 log₁₀ genome copies/mL, 95% CI = [−3.34, 0.46]. For all applicable panels, tests were two-sided, and no adjustment was made for multiple comparisons. *p < 0.05; **p < 0.01; ***p < 0.001; ns not significant. RD risk difference, MD mean difference, CI confidence interval, SEM standard error of the mean, ANOVA analysis of variance. Source data are provided as a Source Data file.

Mice were challenged with a lethal dose of MA EBOV and treated twice daily with MWAC-3634 either orally or by intraperitoneal (IP) injection; vehicle-treated animals served as controls. Each group contained 10 mice. On day 4, three mice per group were randomly selected for serum virus-titer measurements and removed from subsequent survival and clinical assessments. Among the remaining seven mice, survival in the vehicle group was 28.6% (2/7), accompanied by an average 17% weight loss on day 7 (measured in four mice) and a mean clinical score of 2.3 on day 6 (measured in six mice) (Fig. 6A–C). Oral MWAC-3634 improved survival to 85.7% (6/7) with average day-7 weight loss of 7.9% and a mean day-6 clinical score of 0.7, whereas IP administration achieved 100% (7/7) survival with only 2% weight loss and no observable clinical signs (Fig. 6A–C). All clinical parameters in both treatment groups were significantly improved compared with the vehicle control.

Serum virus titers provided additional evidence of potency despite inherent limitations. Because control mice began to die by day 5, day 4 was the latest common sampling time, but this early measurement likely underestimates terminal viral loads, which typically surge immediately before death^41,42^. MWAC-3634 is a viral entry inhibitor that blocks new infection cycles, each requiring roughly 30 h per cell^43^; accordingly, day-4 virus titers capture only the early phase of inhibition. Even at this early point, average virus titers were reduced 176-fold in the oral group and 4019-fold in the IP group relative to controls, with statistical significance achieved only for the IP group (Fig. 6D). The greater reduction in serum virus titer in the IP group aligns with its complete protection and minimal disease signs, whereas the oral group, despite a smaller reduction in virus titer, still achieved near-complete survival and reduced disease signs. Together, these findings support in vivo anti-EBOV activity of MWAC-3634 in this lethal-challenge mouse model. Oral administration improved survival and disease outcomes, while IP delivery produced the greatest virological and clinical effects.

## Discussion

Current interventions against EBOV are limited by accessibility and efficacy, and previously reported small-molecule inhibitors targeting EBOV GP have generally shown only modest potency. Traditional discovery strategies, including cell-based screening, virtual docking, and drug repurposing, have also been constrained by limited chemical space or difficulty in directly targeting GP. These limitations underscore the need for alternative discovery approaches to identify more potent and accessible anti-filovirus therapeutics. In this study, we leveraged the emerging DNA-encoded chemical technology (DEC-Tec) to identify compounds targeting EBOV GP. DEC-Tec enables direct screening for target-binding compounds, eliminating the need for high-throughput protein-based assays^32–35^. It also allowed us to explore a vast chemical space (specifically, 4.73 billion unique compounds) that previous studies were unable to access. From this screen, we identified a hit compound that binds to EBOV GP and carried out two rounds of stereochemical optimization. This process yielded a highly potent compound, MWAC-3634, which binds to EBOV GP with a K_d_ of 40.7 nM and inhibits EBOV infection with an IC_50_ of 0.65 nM. These potency metrics are at least one to two orders of magnitude better than those of previously reported inhibitors. In addition to its high potency, MWAC-3634 demonstrated favorable pharmacokinetic properties, including high oral bioavailability (69% relative to IV), a reasonable in vivo half-life (2.6 h after IV dosing), and prolonged plasma stability (889-min half-life). Notably, total plasma concentrations following oral dosing remained above 2 µM for eight hours, consistent with sustained systemic exposure. Although MWAC-3634 has lower aqueous solubility than toremifene, its high antiviral potency and sustained plasma concentrations after oral dosing in mice support further structure-guided optimization (Table S1)^44,45^. Future analog development will focus on improving solubility and metabolic stability while preserving GP-binding affinity and antiviral potency, guided by the high-resolution GP-MWAC-3634 structure described below. Together, the high potency, lack of detectable cytotoxicity under assay conditions, and encouraging oral exposure support MWAC-3634 as a lead compound for further anti-EBOV therapeutic development.

Our structural study of the EBOV GP ectodomain in complex with MWAC-3634, enabled by the use of an EBOV GP-targeting nanobody to facilitate cryo-EM analysis, provided a high-resolution view of the compound-binding mode. The resulting ternary complex, comprising EBOV GP, MWAC-3634, and the nanobody, was highly stable, allowing us to achieve a resolution of 2.54 Å. The density map of MWAC-3634, along with its detailed interactions with EBOV GP, was well defined. MWAC-3634 binds to a hydrophobic pocket at the GP1/GP2 interface, engaging residues from both GP1 and GP2, including the HR1 and IFL regions from GP2, which are critical for membrane fusion. Its binding induces several notable conformational changes, notably displacing the DFF lid that normally covers the pocket. The precise complementarity between MWAC-3634 and the GP pocket helps explain the compound’s high potency and underscores the success of stereochemical optimization in enhancing its activity. This structural insight lays a strong foundation for rational optimization of the compound to further improve both its potency and pharmacokinetic properties.

The GP1-GP2 interface pocket targeted by MWAC-3634 is also present in other filoviruses, including Sudan virus (SUDV) and Marburg virus (MARV). Our published structures of SUDV and MARV GPs in complex with NPC1 indicate that this pocket is structurally present across these filoviruses, although the MWAC-3634-contacting residues are only partially conserved with respect to EBOV (Fig. S6B)^46,47^. Consistent with this partial conservation, MWAC-3634 retains measurable activity against both SUDV and MARV pseudoviruses, albeit at substantially reduced potency. These findings indicate that the GP1-GP2 interface pocket remains targetable across filoviruses, while sequence and structural variation near the compound-contacting residues shape the potency of GP-stabilizing inhibitors. Together, they identify the GP1-GP2 interface pocket as a tractable vulnerability across filoviruses. Whether future structure-guided optimization of this scaffold can improve cross-filovirus potency will require further study.

MWAC-3634 employs a distinct mechanism to neutralize EBOV entry. All previously characterized EBOV GP-targeting compounds with resolved structures, including toremifene (Table S1), bind to the same pocket at the GP1/GP2 interface and destabilize GP^27,29,30^. This destabilization may be linked to the displacement of the DFF lid, which helps stabilize the GP1/GP2 interface. Interestingly, although MWAC-3634 also displaces the DFF lid, it stabilizes GP rather than destabilizing it. Structural comparison between toremifene and MWAC-3634 reveals that MWAC-3634 forms more extensive contacts across multiple structural elements of GP. These broader stabilizing contacts likely compensate for local structural perturbations associated with DFF lid displacement, resulting in a net stabilization of the prefusion state. Our study points to two distinct mechanisms of EBOV neutralization: one through destabilization and the other through stabilization of GP. EBOV GP is metastable and must overcome an energy barrier to transition from the prefusion to the post-fusion state for membrane fusion^14^. Destabilizing compounds have been proposed to lower this barrier and trigger premature conformational changes, whereas our data support a model in which MWAC-3634 stabilizes prefusion GP, raises the barrier to GP conformational transition, and blocks viral entry. This model is inferred from thermostability assays, high-affinity GP binding, cryo-EM structural analysis, and viral entry inhibition. The markedly higher potency of MWAC-3634 compared to previously identified compounds suggests that GP stabilization is an alternative and effective mechanism for inhibiting EBOV entry. Together, these findings support a model in which EBOV GP function depends on a finely tuned metastable state, where deviations in either direction disrupt productive entry. Similar stabilization-based neutralization mechanisms have been described in other virus families, including fatty acid stabilization of the RBD-closed conformation of the SARS-CoV-2 spike protein to prevent ACE2 receptor binding and pocket factor stabilization of enterovirus capsids to prevent uncoating^48,49^. However, in filoviruses, this mechanism is distinguished by inhibition of the prefusion-to-postfusion transition. Overall, our findings raise the possibility that small-molecule stabilization of filovirus GPs may provide a strategy for inhibiting filovirus entry.

Although this study primarily focuses on the discovery and mechanism of anti-EBOV compounds, we also demonstrate, as a proof of concept, that MWAC-3634 improves outcomes in a lethal EBOV challenge mouse model. Twice-daily dosing by either the intraperitoneal (IP) or oral route improved survival and reduced weight loss and clinical disease relative to vehicle controls. IP administration was associated with full survival in this study and *a* ~ 4019-fold reduction in serum viral load by day 4, whereas oral dosing was associated with substantially improved survival and *a* ~ 176-fold reduction, although this did not reach statistical significance at this early time point. The greater viral suppression with IP dosing may reflect more consistent systemic exposure; notably, measurable effects were also observed following oral dosing, which is relevant given the logistical advantages of noninvasive administration during outbreaks. Decreased serum viral titers by day 4 are consistent with early antiviral activity, although these measurements likely underestimate peak viremia because control animals began to succumb by day 5. Moreover, because MWAC-3634 inhibits viral entry, thereby preventing subsequent rounds of infection that unfold on the order of ~30 h per replication cycle, its full downstream antiviral effects may not be fully captured within this early sampling window. Collectively, these results support further evaluation of MWAC-3634 as a lead series for EBOV therapeutics. Because the mouse-adapted EBOV strain differs from wild-type virus and murine disease kinetics differ from human infection, translation of potency will require evaluation in additional preclinical models, particularly non-human primates. Going forward, pharmacokinetic optimization will be essential to strengthen in vivo performance and enable rigorous evaluation in non-human primate models. Given its distinct mechanism of action as an entry inhibitor, MWAC-3634 may also be worth evaluating in combination with other inhibitors, such as remdesivir or obeldesivir^11,12^, that target a different stage of the EBOV infection cycle, for benchmarking and to assess potential combination therapy.

In summary, using DEC-Tec, we identified MWAC-3634, a potent EBOV entry inhibitor with substantially greater GP-binding affinity and anti-EBOV potency than previously reported inhibitors. Its high therapeutic index indicates a favorable safety profile. The compound blocks EBOV entry through a proposed distinct mechanism, providing mechanistic insight into how stability tuning governs filovirus entry. MWAC-3634 also exhibits favorable oral pharmacokinetic properties. As a proof of concept, in a lethal-challenge mouse model, treatment improved survival and clinical disease outcomes following both IP and oral dosing. These findings position MWAC-3634 as a potent and promising lead compound for further therapeutic development. Our nanobody-enabled, high-resolution cryo-EM strategy provides a structural platform to support continued optimization of GP-stabilizing anti-filovirus inhibitors and to further suggest stability tuning as a mechanistically distinct mode of filovirus entry inhibition.

## Methods

### Ethics statement

This study was performed in strict accordance with the recommendations in the Guide for the Care and Use of Laboratory Animals of the National Institutes of Health. All of the animals were handled according to approved institutional animal care and use committee (IACUC) protocols at Boston University (protocol number: 201900062) and at the University of Florida Scripps Biomedical Research (protocol number: 15-022-04).

### Cell lines, plasmids and viruses

HEK293T and HeLa cells (American Type Culture Collection, ATCC; CRL-3216 and CCL-2, respectively) and Huh-7 cells (Applied Biological Materials; T8973) were cultured in DMEM (Dulbecco’s Modified Eagle Medium; Gibco) supplemented with 10% FBS (fetal bovine serum; GeminiBio), 2 mM L-glutamine, and 100 U/mL P/S (penicillin-streptomycin; Gibco). Expi293F cells (Thermo Fisher Scientific; A14527) were maintained in Expi293 Expression Medium (Thermo Fisher Scientific). The cell lines were obtained from commercial vendors that authenticate their cell lines. They were not independently authenticated in our laboratories. No commonly misidentified cell lines were used in this study.

The EBOV *GP* gene (Zaire strain; NCBI Reference Sequence NC_002549.1), the SUDV *GP* gene (Gulu strain; NCBI Reference Sequence NC_006432.1), and the MARV *GP* gene (Musoke strain; NCBI Reference Sequence DQ217792.1) were synthesized (GenScript). For pseudovirus production, the full-length EBOV, SUDV, and MARV *GP* genes were individually cloned into the pcDNA3.1(+) vector with a C-terminal C9 tag. For protein expression, the gene encoding the EBOV GP ectodomain (residues 1–632), with the mucin-like domain deleted (residues 313–463), was cloned into the Lenti-CMV vector (Vigene Biosciences). For simplicity, this construct is referred to as the GP ectodomain. A C-terminal foldon trimerization motif followed by a His tag was included to promote trimer formation and facilitate purification.

Authentic EBOV (H.sapiens-tc/COD/1976/Yambuku–Mayinga, wild type [WT]) was used to infect HeLa cells in vitro. A mouse-adapted (MA) EBOV strain used in this study, bearing one GP substitution (S246P) and eight additional genomic substitutions, was derived from a previously reported mouse-adapted strain (GenBank AF499101)^50^. This MA strain had GP residues 65 and 544 reverted to the WT residues because these two residues are located at or close to the MWAC-3634-binding region. The GP S246P mutation was retained because residue 246 is in a disordered region not mapped in our structural model and is therefore unlikely to directly affect MWAC-3634 binding. The eight mutations in other parts of the viral genome were also retained because they are outside GP and are not expected to affect compound binding to GP. The MA strain was further assessed for in vivo infection in mice, confirming its suitability for evaluating MWAC-3634 in vivo. All work with authentic EBOV was performed in approved biosafety level-4 (BSL-4) laboratories at Boston University.

### Protein expression and purification

The EBOV GP ectodomain was expressed and purified from mammalian cells^37^. Plasmids encoding the EBOV GP ectodomain were transiently transfected into Expi293F cells using polyethylenimine (PEI; Polysciences). Three days post-transfection, the protein was collected from the culture supernatant and purified using a Ni-NTA affinity column, followed by further purification on a Superose 6 Increase 10/300 gel filtration column (Cytiva).

His-tagged Nanosota-EB2 was expressed and purified from the periplasm of ss320 *E. coli*^37^. Protein expression was induced with 1 mM IPTG. Cell pellets were collected and resuspended in 15 mL TES buffer (0.2 M Tris, pH 8.0; 0.5 mM EDTA; 0.5 M sucrose), then shaken on ice for 1 h. The suspension was diluted with 40 mL of ¼-strength TES buffer (each component at one-quarter concentration) and shaken on ice for another hour. The supernatant was collected, and the protein was purified sequentially using a Ni-NTA affinity column followed by a Superdex 200 gel filtration column (Cytiva).

### DEC-Tec screens for EBOV GP-targeting compounds

Affinity selection using DEC-Tec libraries was performed as follows^32–36^. Previously reported DEC-Tec libraries, comprising a total of 4.73 billion molecules, were pooled and incubated with 1 μM His-tagged EBOV GP ectodomain in selection buffer (25 mM Tris, 150 mM NaCl, 1 mM TCEP, 10 mM imidazole, 0.02% Tween-20, and 0.1 mg/mL sheared salmon sperm DNA). A parallel no-target control (NTC) pool, identical in composition but without target protein, was incubated under the same conditions to assess background binding to the affinity resin. Prior to incubation, 2 μL of the library pool was collected for quantification by qPCR. Ni-NTA magnetic beads (50 μL) were prewashed with 500 μL of selection buffer, then incubated with the target protein-library mixture for affinity selection. Selection and informatics procedures were then carried out using established DEC-Tec protocols^32–36^. Sequencing was performed at the Baylor College of Medicine Genomic and RNA Profiling Core Facility using an Illumina NextSeq 500 (San Diego, CA). Selection output data were analyzed using in-house data pipelines^32–36^.

### Chemical synthesis of EBOV GP-targeting compounds

Chemical synthesis of EBOV-targeting compounds was described in Protocol S1.

### Plasma protein binding

Plasma protein binding was determined using equilibrium dialysis. All samples were tested in duplicate using the RED Rapid Equilibrium Dialysis Device (Thermo Scientific). The initial drug concentration in the plasma chamber was 2 µM, and phosphate-buffered saline (PBS) was added to the receiver chamber. The plate was covered and shaken in a 37 °C incubator for 6 h. A 25 µL aliquot was collected from each of the plasma and PBS chambers, and samples were diluted with either blank PBS or plasma to achieve a 1:1 plasma ratio for all samples. Drug concentrations in the plasma and PBS chambers were determined by LC-MS/MS. The fraction bound was calculated as follows: fraction bound = ([plasma]–[PBS])/[plasma].

### Plasma stability

Plasma stability was evaluated by incubating 10 µM test compound with undiluted plasma at 37 °C, with aliquots removed at multiple time points up to 4 h. Aliquots were added to acetonitrile (5:1, v/v) to stop enzymatic activity and were held on ice. Samples were centrifuged through a Millipore Multiscreen Solvinter 0.45 µm low-binding hydrophilic PTFE filter plate and analyzed by LC-MS/MS. The percentage of compound remaining at each time point was log-transformed, and the slope of the linear regression was used to determine the elimination rate constant. Half-life was calculated using the equation: half-life = ln(2)/−slope.

### Hepatic microsomal stability

Hepatic microsomal stability was evaluated by incubating 1 µM test compound with 0.5 mg/mL hepatic microsomes in 100 mM potassium phosphate buffer, pH 7.4. The reaction was initiated by adding NADPH to a final concentration of 1 mM. Aliquots were removed at 0, 5, 10, 20, 40, and 60 min and added to acetonitrile (5:1, v/v) to stop the reaction and precipitate protein. NADPH dependence was evaluated by performing parallel incubations without NADPH. At the end of the assay, samples were centrifuged through a Millipore Multiscreen Solvinter 0.45 µm low-binding hydrophilic PTFE filter plate and analyzed by LC-MS/MS. Data were fit to a one-phase exponential decay model using 1/Y weighting, with the plateau constrained to 0.

### Pharmacokinetics of EBOV GP-targeting compounds

The pharmacokinetics of MWAC-3634 were evaluated in female C57BL/6J mice (16 weeks old; Jackson Laboratory). Mice were maintained on a 12-h light/12-h dark cycle, at a target ambient temperature of 22.2–23.3 °C and a target relative humidity of 45–55%, with food and water available ad libitum. The compound was formulated as a clear solution consisting of 10% DMSO, 10% Tween 80, and 80% water, and administered at 5 µL/g body weight intravenously (IV) or 10 µL/g by oral gavage. Doses were 2 mg/kg IV and 50 mg/kg orally. Three mice per group were dosed, and blood samples were collected at 0.08, 0.25, 0.5, 1, 2, 4, 6, and 8 h post-dose. A microsampling strategy was employed using EDTA-containing capillary tubes to collect approximately 25 µL of blood (~ 10–12 µL plasma). A standard curve was prepared in blank mouse plasma and processed alongside the pharmacokinetic samples. Plasma (5 µL) was treated with 75 µL of 90% acetonitrile containing carbamazepine as an internal standard. The resulting precipitate was cleared by filtration through a 0.2 µm filter plate. MWAC-3634 concentrations were quantified by liquid chromatography-tandem mass spectrometry (LC-MS/MS) using the transition m/z 733 → 306 and normalized to the internal standard signal (m/z 237 → 194). Pharmacokinetic parameters were calculated using a non-compartmental model (Phoenix WinNonlin, Pharsight Inc.).

### Surface plasmon resonance

Surface plasmon resonance (SPR) was used to evaluate the binding of EBOV GP-targeting compounds to the EBOV GP ectodomain using a Biacore S200 system (Cytiva). Recombinant EBOV GP ectodomain was immobilized on a CM5 sensor chip (Cytiva) via chemical crosslinking. Test compounds were injected at the indicated concentrations in a running buffer composed of 10 mM HEPES (pH 7.4), 150 mM NaCl, 3 mM EDTA, and 0.05% Tween-20. SPR data were analyzed using Biacore Evaluation Software (Cytiva).

### Neutralizing potency of filovirus GP-targeting compounds against filovirus pseudoviruses

The neutralizing potency of filovirus GP-targeting compounds against EBOV, SUDV, and MARV pseudoviruses was assessed^37,47^. Pseudoviruses were generated by co-transfecting HEK293T cells with a pcDNA3.1(+) plasmid encoding full-length EBOV, SUDV, or MARV GP, the helper plasmid psPAX2, and the reporter plasmid plenti-CMV-luc. After 72 h, pseudoviruses were harvested and incubated with varying concentrations of each compound for 1 h at 37 °C, then used to infect Huh7 cells. Following a 48-h incubation, the cells were lysed, and aliquots of the lysates were transferred to new plates. A luciferase substrate was added, and Relative Light Units (RLUs) were measured using an EnSpire plate reader (PerkinElmer). Compound potency was quantified as the concentration required to inhibit pseudovirus entry by 50% (IC₅₀).

### Cell viability assay

Cell viability was evaluated using the CellTiter 96 AQueous One Solution Cell Proliferation Assay (MTS; Promega, Madison, WI, USA) according to the manufacturer’s instructions. Huh7 cells were seeded in 96-well plates at the indicated density and allowed to adhere overnight at 37 °C in a humidified incubator with 5% CO_2_. Cells were treated with serial dilutions of the compounds under the same conditions and for the same duration as in the pseudovirus entry assay. Following treatment, CellTiter 96 AQueous One Solution reagent was added directly to each well (20 µL reagent per 100 µL culture medium), and the plates were incubated at 37 °C for 2 h. Absorbance was measured at 490 nm using a microplate reader. Cell viability was expressed relative to untreated control cells, which were set to 100%.

### Thermal stability assay

To evaluate the impact of EBOV GP-targeting compounds on the thermal stability of EBOV GP, differential scanning fluorimetry was performed as follows^37^. 20 μg of EBOV GP ectodomain was incubated with varying amounts of an EBOV GP-targeting compound at room temperature for 30 min. The sample was then mixed with 1.25 μL of Protein Thermal Shift Dye (ThermoFisher), diluted in 10 μL of PBS, in a 96-well MicroAmp optical qPCR plate. For the control group, 20 μg of EBOV GP ectodomain was incubated with the dye alone. Measurements were performed using a PCR instrument (Applied Biosystems) with a temperature ramp from 25 to 99 °C at a rate of 0.05 °C/s. Data were collected using QuantStudio real-time PCR software. The negative first derivative of fluorescence was plotted against temperature, and the melting temperature (Tm) was determined as the peak of the first derivative curve.

### Cryo-EM data collection, data processing, model building and refinement

The EBOV GP ectodomain/Nanosota-EB2 complex (0.25 mg/mL) was mixed with 100 µM compound and incubated at room temperature for 1 h prior to grid preparation. A 4 µL aliquot of the sample was applied to freshly glow-discharged Quantifoil R1.2/1.3 300-mesh copper grids (Electron Microscopy Sciences), blotted for 4 s at 22 °C under 100% humidity, and plunge-frozen in liquid ethane using a Vitrobot Mark IV (FEI). A total of 6,106 images were collected in CDS mode using a K3 Summit detector (Gatan) with a BioQuantum GIF energy filter (20 eV slit width), attached to a 300 kV FEI Titan-Krios TEM. Cryo-EM data collection statistics are summarized in Table S2.

Cryo-EM data were processed using cryoSPARC v4.4.1^51^, following the workflow shown in Fig. S8. All movies were motion-corrected using MotionCor2^52^ and contrast transfer functions (CTFs) were estimated with CTFFIND-4.1.13^53^. Particles were extracted and downsampled by a factor of 3/4. Particle selection was performed using the Blob Picker in cryoSPARC, followed by three rounds of 2D classification to remove junk particles. High-quality 2D classes were used for ab initio reconstruction of three maps, followed by heterogeneous refinement. Particles from the best classes were subjected to non-uniform refinement and CTF refinement, yielding the final maps. Map resolution was determined using the gold-standard Fourier shell correlation (FSC) at the 0.143 threshold between the two half-maps. Local resolution was estimated using cryoSPARC v4.4.1.

Initial model building was performed in Coot-0.8.9^54^, using PDB entry 9BSV as the starting model. Additional distinct densities were observed in the pocket at the GP1–GP2 interface, into which the compound was fitted. Refinement was carried out using Phenix v1.16^55^, along with manual adjustments in Coot v0.8.9. Model and map statistics are summarized in Table S2. Figures were prepared using UCSF ChimeraX v0.93^56^ and PyMOL Molecular Graphics System, Version 3.0 (Schrödinger, LLC). Interacting residues between the compound and GP were analyzed using LigPlot.

### Neutralizing potency of EBOV GP–targeting compounds against authentic EBOV in vitro

Two EBOV strains were used to test the neutralizing potency of MWAC-3634: wild type (WT) and mouse-adapted (MA). For all other compounds, only the WT strain was tested.

To generate the MA strain, a previously reported mouse-adapted virus^50^ was first engineered by introducing 11 mutations, including three GP substitutions (S65P, S246P, I544T), into shuttle vectors containing six EBOV genomic fragments using site-directed mutagenesis. The fragments were amplified by PCR and assembled by circular polymerase extension reaction (CPER), and virus rescue was performed. To generate the MA strain used in this study, CPER was used to revert GP residues P65 and T544 to S65 and I544, respectively, producing a virus that retained only GP S246P along with the same eight additional genomic substitutions.

EBOV infection efficiency was quantified by focus-forming assay (FFA). The two EBOV strains were titered on HeLa cells^57^. HeLa cells were seeded in 96-well plates 24 h before infection, incubated with fivefold serial dilutions of virus for 30 min at 37 °C, 5% CO₂, and overlaid with 0.25% methylcellulose. At 48 h post-infection, plates were fixed in 10% formalin and stained with EBOV GP mAb 4F3 (1:3000; IBT 0201-020, Rockville, MD, USA). Foci comprising 3–5 infected cells were enumerated to calculate focus-forming units per mL (FFU/mL).

Dose-response assays were used to assess neutralizing potency against authentic EBOV. HeLa cells were seeded in 384-well plates 24 h prior to infection. Medium was replaced with 40 µL DMEM containing 2% FBS and 100 U/mL penicillin–streptomycin (Gibco). Each compound of interest was dispensed using an I.DOT Liquid Handler (Dispendix) to generate a 20-point, twofold serial dilution from 625 to 0.0012 nM. Ten microliters of virus inoculum (multiplicity of infection, MOI = 0.1–0.3) was added per well. After 48 h, cells were formalin-fixed and stained with EBOV GP 4F3 and Hoechst 33342 (Thermo Fisher). Plates were imaged on a Cytation 1 Imaging Reader (BioTek) using DAPI and GFP channels. Images were processed with CellProfiler v4.2.6 to quantify nuclei and infected cells. Potency was defined as the concentration reducing virus counts by 50% (IC₅₀) relative to virus-only controls. Dose-response curves were generated in triplicate for each virus.

### In vivo potency in a lethal-challenge mouse model

Potency of MWAC-3634 against MA EBOV was evaluated in BALB/cJ mice (5–8 weeks old; Jackson Laboratory)^58^. Mice were maintained on a 12-h light/12-h dark cycle, at a target ambient temperature of 20.0–22.2 °C and a target relative humidity of 50%, with food and water available ad libitum. Mice were randomized into groups of 10 with equal numbers of males and females. All animals were challenged on day 0 with 2000 FFU of MA EBOV in 0.1 mL PBS via intraperitoneal (IP) injection.

MWAC-3634 was formulated in 10% DMSO/10% Tween-80/80% H₂O. A dose of 25 mg/kg was administered in 100 µL per mouse by IP or oral administration at 6 h post-infection, then twice daily from days 1–8. Animals were monitored for survival, weight, and clinical signs; observations were conducted twice daily on days 3–10. Disease scoring included eye appearance, general appearance, responsiveness, and body-weight loss >11%. Humane euthanasia followed AVMA and AAALAC guidelines when ≥3 of 4 criteria were met over two consecutive observations; mice with ≥20% weight loss were also euthanized.

On day 4, three animals per group were randomly selected for serum collection and then euthanized; these animals were excluded from survival analyses after day 4. Remaining animals were followed through day 16. Day-4 sera were inactivated with TRIzol LS (Invitrogen), RNA was extracted using the Direct-zol RNA Miniprep Kit (Zymo Research), and EBOV *NP* RNA was quantified by RT-qPCR (Luna One-Step Universal Probe RT-qPCR Kit) using a standard curve generated from in-vitro–transcribed EBOV *NP* RNA. Viral loads are reported as genome copies per mL^37^.

### Statistical analysis of mouse study

GraphPad Prism (v10.3.0) was used for data analysis. Survival curves were generated using the Kaplan–Meier method, and each treatment group was compared with the vehicle control using the Mantel–Cox (log-rank) test. Body weight was recorded daily for each animal available at the time of measurement, and percent change was calculated relative to that animal’s baseline. Animals that died on a given day were included in that day’s body-weight data if their weight had been recorded. Clinical scores were recorded daily only for animals that were alive at the time of assessment. Viral load was measured on day 4 only for a subset of three animals per group, expressed as genome copies per milliliter and log-transformed prior to analysis. Percent weight change (day 7), clinical scores (day 6), and viral load (day 4) were analyzed by one-way ANOVA, comparing each treatment group with the vehicle control group.

### Reporting summary

Further information on research design is available in the Nature Portfolio Reporting Summary linked to this article.

## Supplementary information

Supplementary Information

Reporting Summary

Transparent Peer Review file

## Source data

Source Data

## Data Availability

The atomic model and corresponding cryo-EM density map of the ternary complex comprising EBOV GP, MWAC-3634, and Nanosota-EB2 have been deposited in the Protein Data Bank and Electron Microscopy Data Bank under accession codes 9PEL and EMD-71566, respectively. Previously published sequence data used in this study are available under accession codes NC_002549.1, NC_006432.1, DQ217792.1, and AF499101. Previously published structural data are available under accession codes 9BSV and 5JQ7. Source data are provided with this paper.
