# Supplementary material for: A small molecule inhibits Ebola virus entry through glycoprotein stabilization
Source: Nat Commun. 2026 Sep 1;17:10401. doi: 10.1038/s41467-026-77332-3 (PMC13627709; doi:10.1038/s41467-026-77332-3)
Supplement: Supplementary file 1 [file 41467_2026_77332_MOESM1_ESM.pdf]

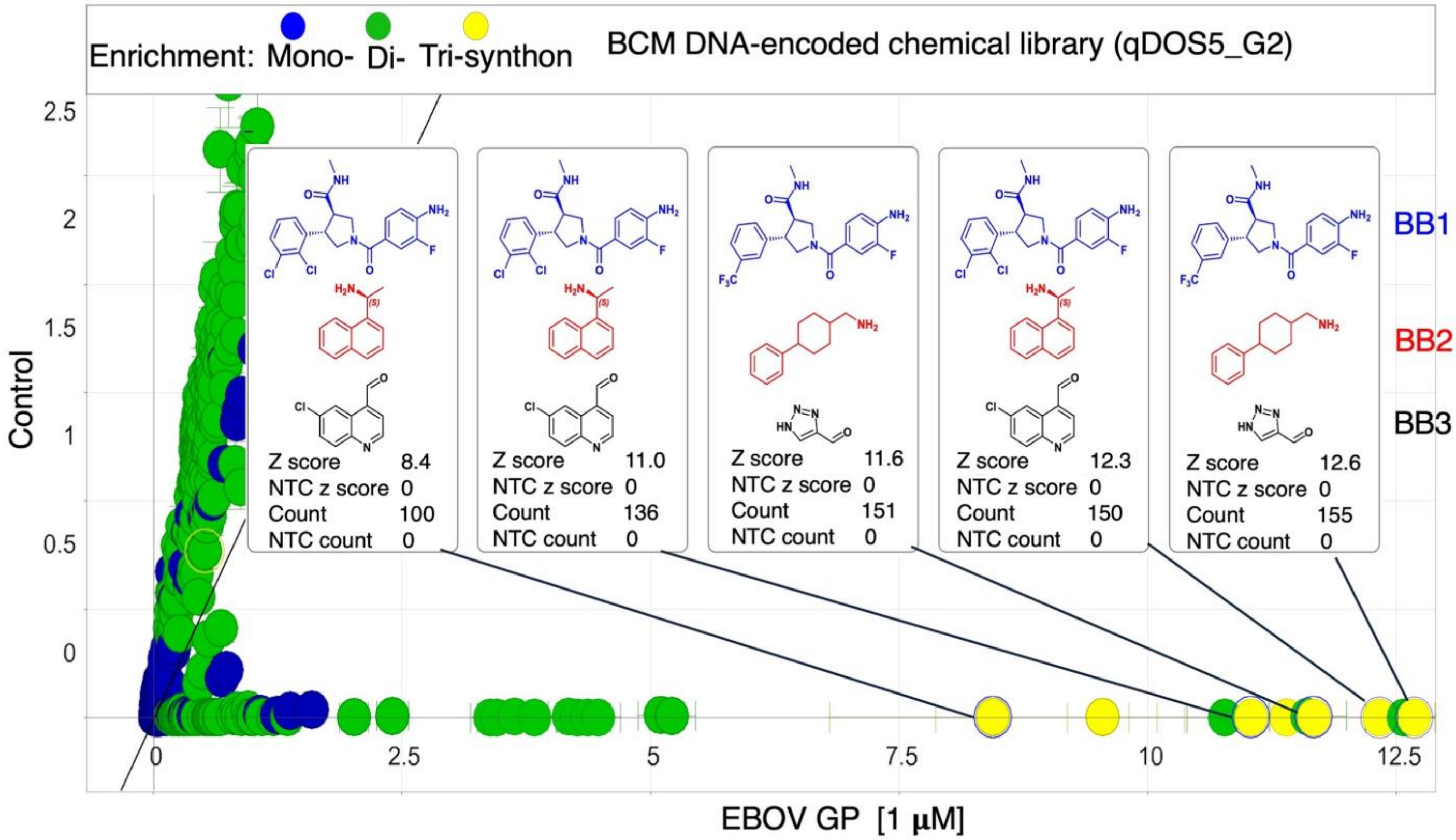


**Figure S1. DEC-Tec screening.** Enrichment profile of the BCM DNA-encoded chemical library qDOS5_G2 against the EBOV GP ectodomain at 1 μM, plotted as z-scores: target binding (x-axis) versus no-target control (y-axis). A series of hit compounds sharing common building blocks were identified: BB1 (blue; DNA-attached), BB2 (red), and BB3 (black). Blue, green, and yellow circles represent mono-, di-, and tri-synthons, respectively. The enrichment of each tri-synthon is displayed as sequencing z-scores/counts at 1 μM (boxed). NTC, no-target control.

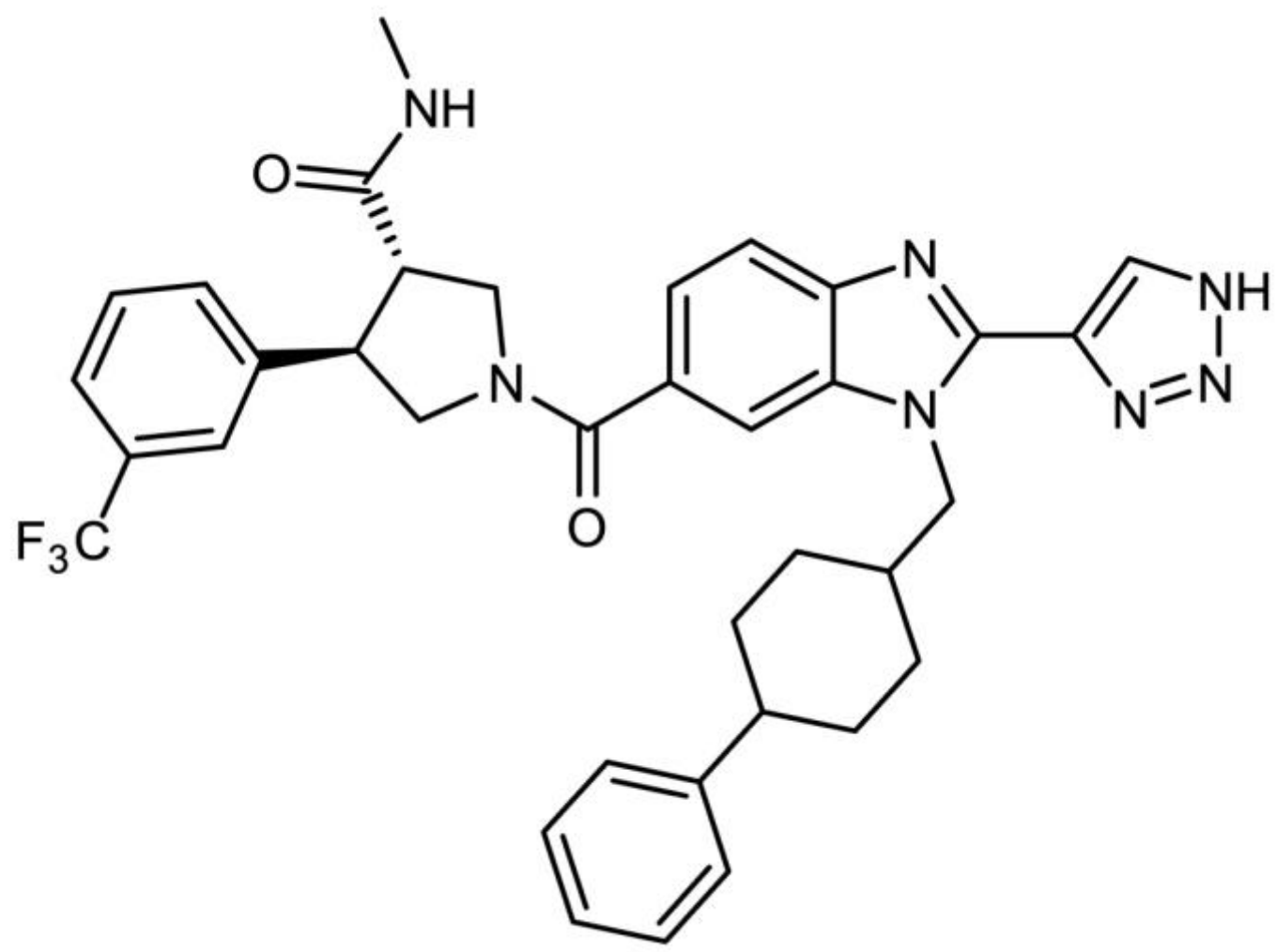

**MWAC-2601**

$IC_{50}$ (pseudovirus) = 51 μM
$CC_{50}$ > 10 μM

**Figure S2. Structure and antiviral activity of MWAC-2601.** $IC_{50}$, half-maximal inhibitory concentration; $CC_{50}$, half-maximal cytotoxic concentration.

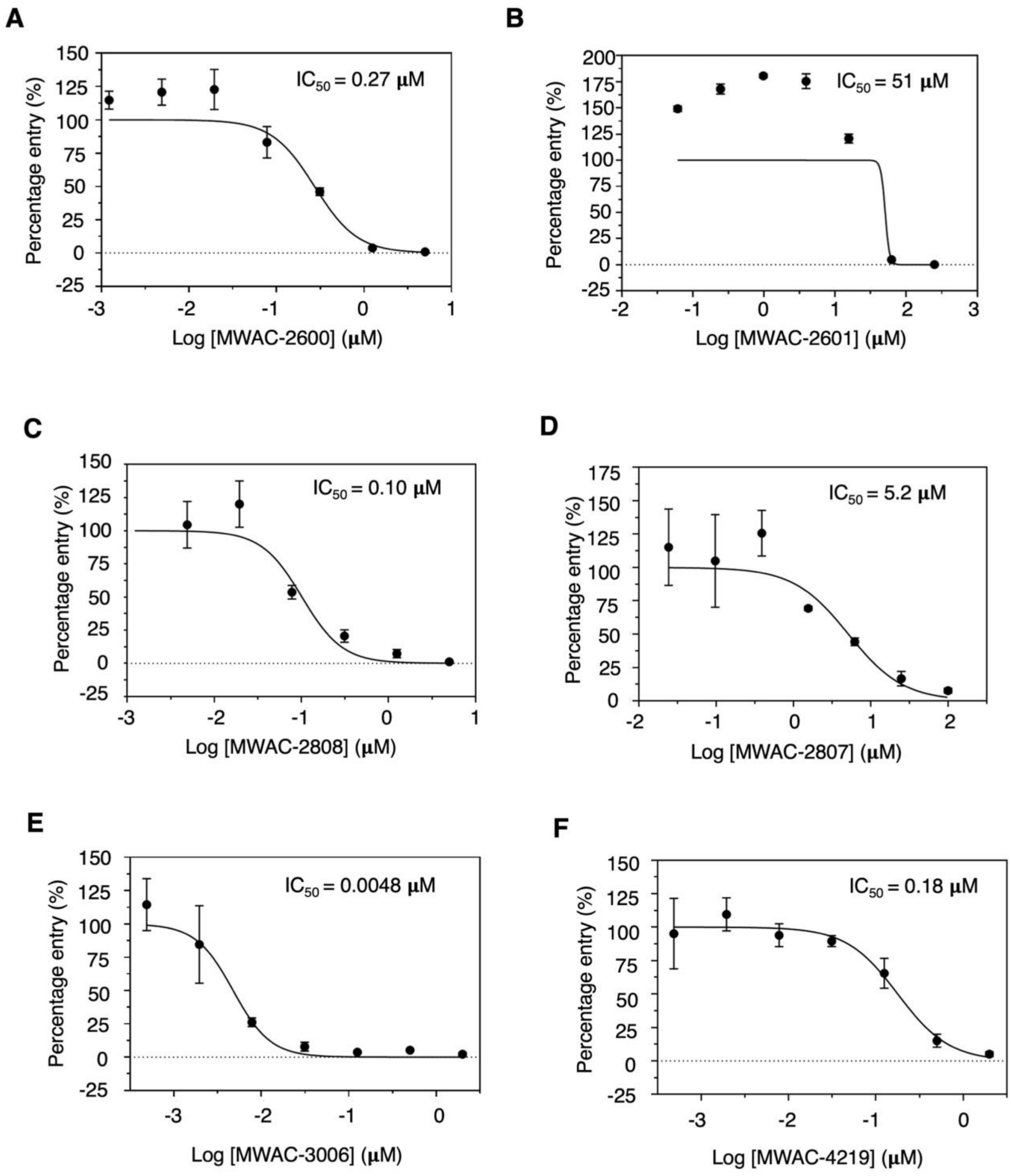


**Figure S3. Dose-response analysis of EBOV GP-pseudovirus entry inhibition by MWAC compounds.** EBOV GP-pseudotyped retroviruses were incubated with serial dilutions of each compound for 1 h at 37°C before infection of Huh7 cells. Pseudovirus entry was quantified 48 h post-infection using luciferase reporter activity. Dose-response curves are shown for **(A)** MWAC-2600, **(B)** MWAC-2601, **(C)** MWAC-2808, **(D)** MWAC-2807, **(E)** MWAC-3006, and **(F)** MWAC-4219. $IC_{50}$ values are indicated in each panel. Circles indicate mean values, and solid lines indicate fitted dose-response curves. Data are presented as mean ± SEM (*n* = 3 technical replicate wells per concentration). $IC_{50}$, half-maximal inhibitory concentration; SEM, standard error of the mean. Source data are provided as a Source Data file.

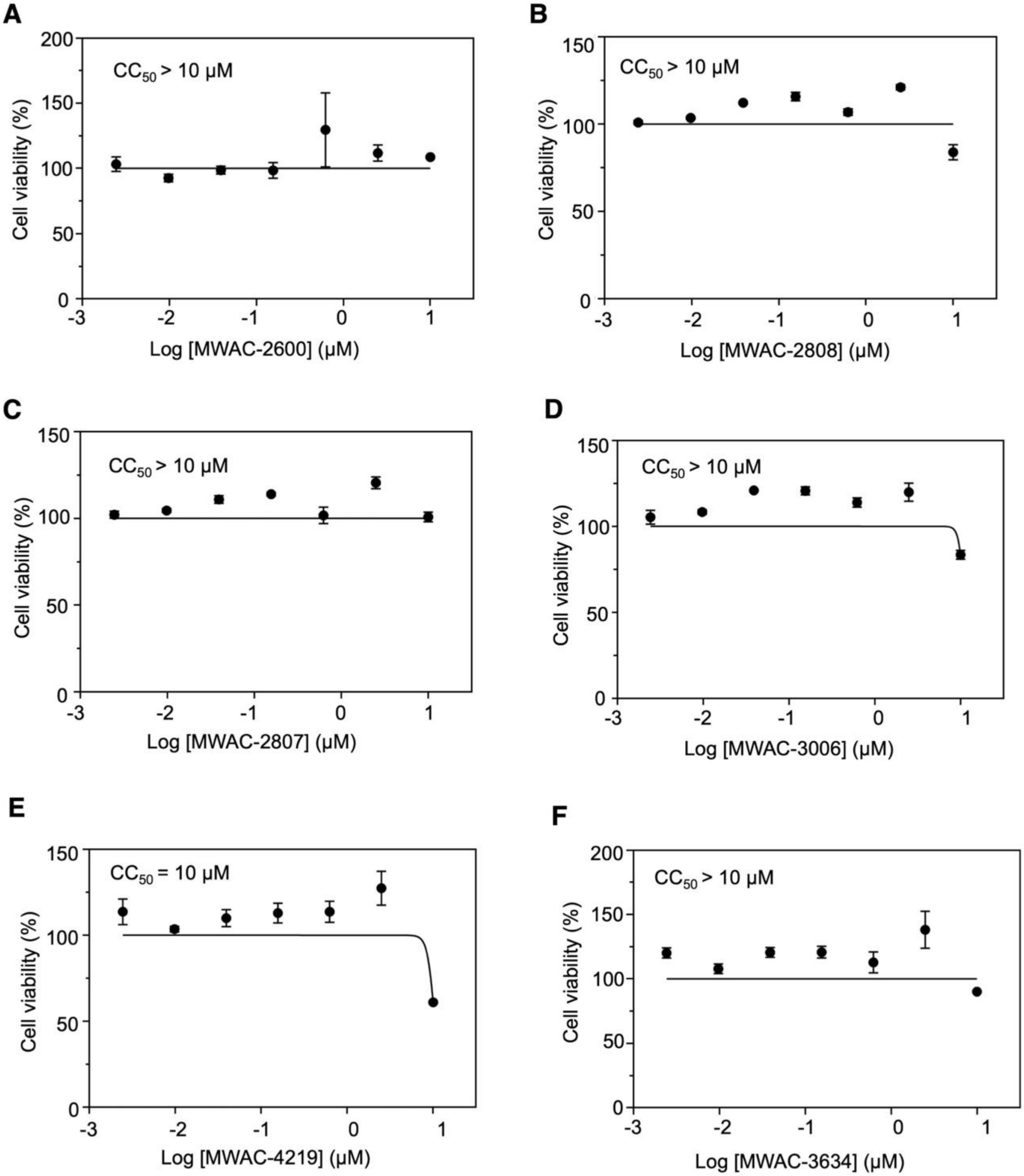


**Figure S4. Dose-response analysis of MWAC compound cytotoxicity in Huh7 cells.** Huh7 cells were incubated with serial dilutions of each compound under the same compound-exposure conditions used for EBOV GP-pseudovirus entry assays. Cell viability was quantified and normalized to vehicle-treated cells. Dose-response curves are shown for **(A)** MWAC-2600, **(B)** MWAC-2808, **(C)** MWAC-2807, **(D)** MWAC-3006, **(E)** MWAC-4219, and **(F)** MWAC-3634. $CC_{50}$ values are indicated in each panel. Circles indicate mean values, and solid lines indicate fitted dose-response curves. Data are presented as mean ± SEM (*n* = 3 technical replicate wells per concentration). $CC_{50}$, half-maximal cytotoxic concentration; SEM, standard error of the mean. Source data are provided as a Source Data file.

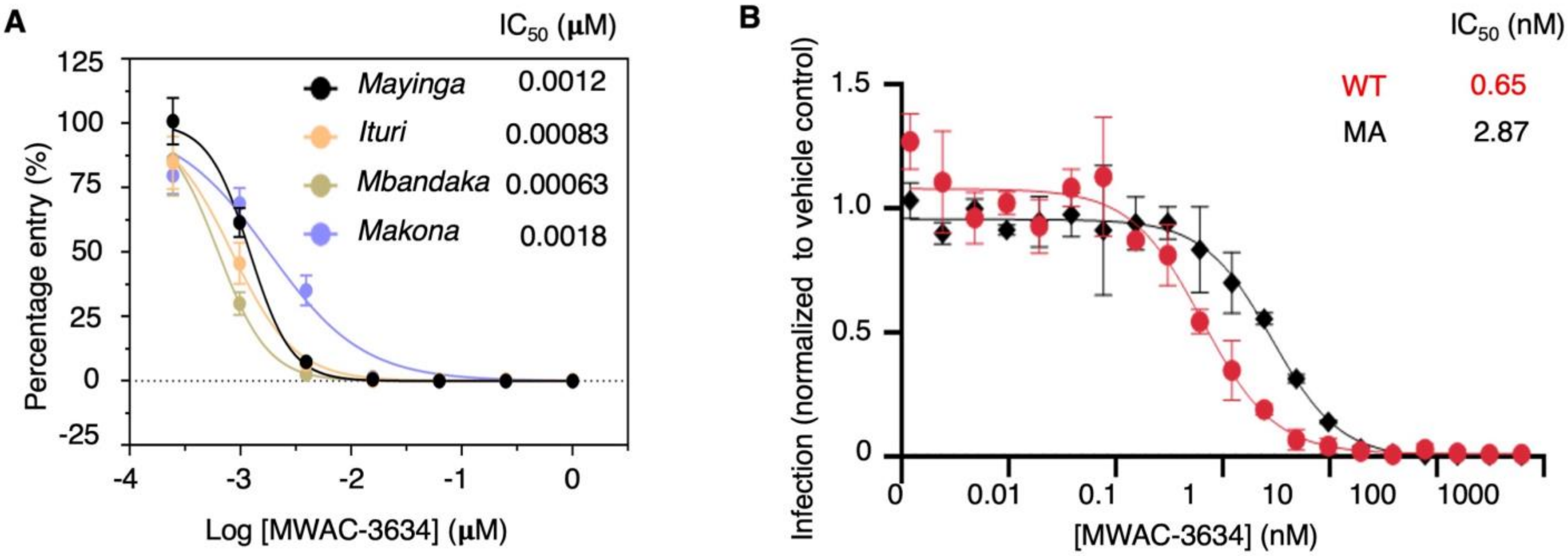


**Figure S5. Antiviral activity of MWAC-3634 against multiple EBOV strains. (A)** Neutralizing potency of MWAC-3634 was measured against EBOV GP-pseudoviruses representing the Mayinga, Ituri, Mbandaka, and Makona strains. $IC_{50}$ values are indicated in the panel. Mayinga, Ituri, Mbandaka, and Makona are shown as black, orange, tan, and blue circles, respectively. **(B)** Neutralizing potency of MWAC-3634 was measured against two strains of authentic EBOV in HeLa cells: wild-type EBOV (WT) and a mouse-adapted strain (MA) with one GP substitution (S246P) and additional substitutions in other genes. $IC_{50}$ values are indicated in the panel. WT and MA are shown as red circles and black diamonds, respectively. Data are presented as mean ± SEM (*n* = 3 technical replicate wells per concentration for each strain). $IC_{50}$, half-maximal inhibitory concentration; SEM, standard error of the mean. Source data are provided as a Source Data file.

**A**

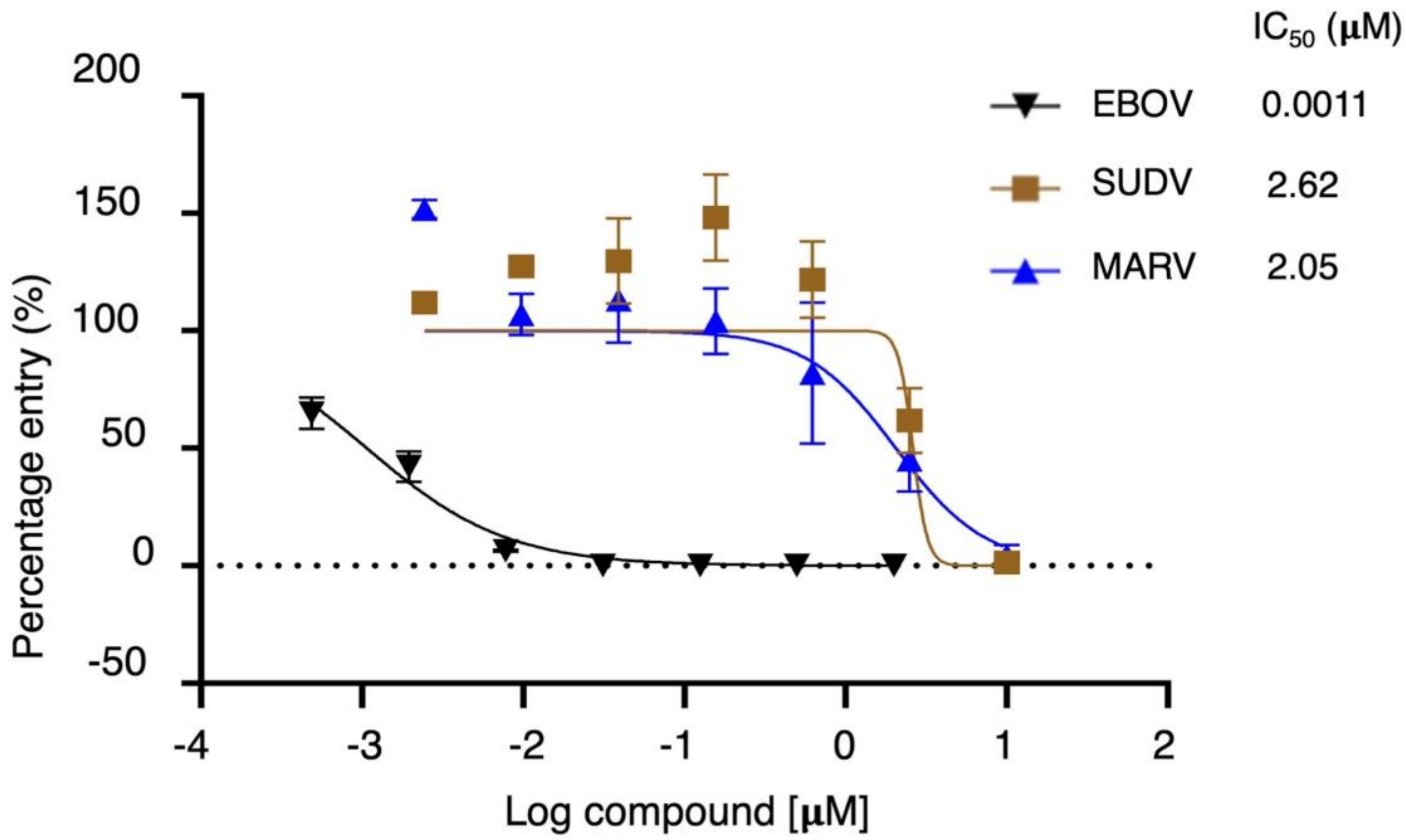


**B**

| | 38 | 43 | 64 | 65 | 66 | 100 | 101 | 186 | 187 | 189 | 515 | 517 | 519 | 548 | 551 | 554 | 555 | 558 |
|---|---|---|---|---|---|---|---|---|---|---|---|---|---|---|---|---|---|---|
| EBOV | I | L | R | S | V | E | A | L | P | A | L | Y | T | M | Q | L | I | L |
| SUDV | V | L | K | S | V | E | A | L | K | T | L | Y | T | M | Q | L | V | L |
| MARV | - | - | H | L | M | T | E | M | I | S | L | I | S | I | Q | L | V | L |
| | | | : | . | : | : | . | : | . | . | * | : | : | : | * | * | : | * |

**Figure S6. Antiviral activity of MWAC-3634 against filovirus GP-pseudoviruses and conservation of GP contact residues. (A)** Retroviruses pseudotyped with GPs from EBOV, SUDV, or MARV were used to infect Huh7 cells in the presence of increasing concentrations of MWAC-3634. Entry efficiency was quantified by luciferase reporter activity. Black downward triangles, brown squares, and blue upward triangles represent EBOV, SUDV, and MARV, respectively. $IC_{50}$ values are indicated in the panel. Data are presented as mean ± SEM ($n$ = 3 technical replicate wells per concentration for each virus). Because MWAC-3634 was highly potent against EBOV GP-pseudoviruses, inhibition at the lowest compound concentrations was not fully resolved, which may affect the precision of the calculated EBOV $IC_{50}$. **(B)** Alignment of filovirus GP residues corresponding to the EBOV MWAC-3634 contact residues shown in Fig. 5A. Asterisks, colons, and periods indicate identical, strongly similar, and weakly similar residues, respectively. EBOV, Ebola virus; SUDV, Sudan virus; MARV, Marburg virus; $IC_{50}$, half-maximal inhibitory concentration; SEM, standard error of the mean. Source data are provided as a Source Data file.

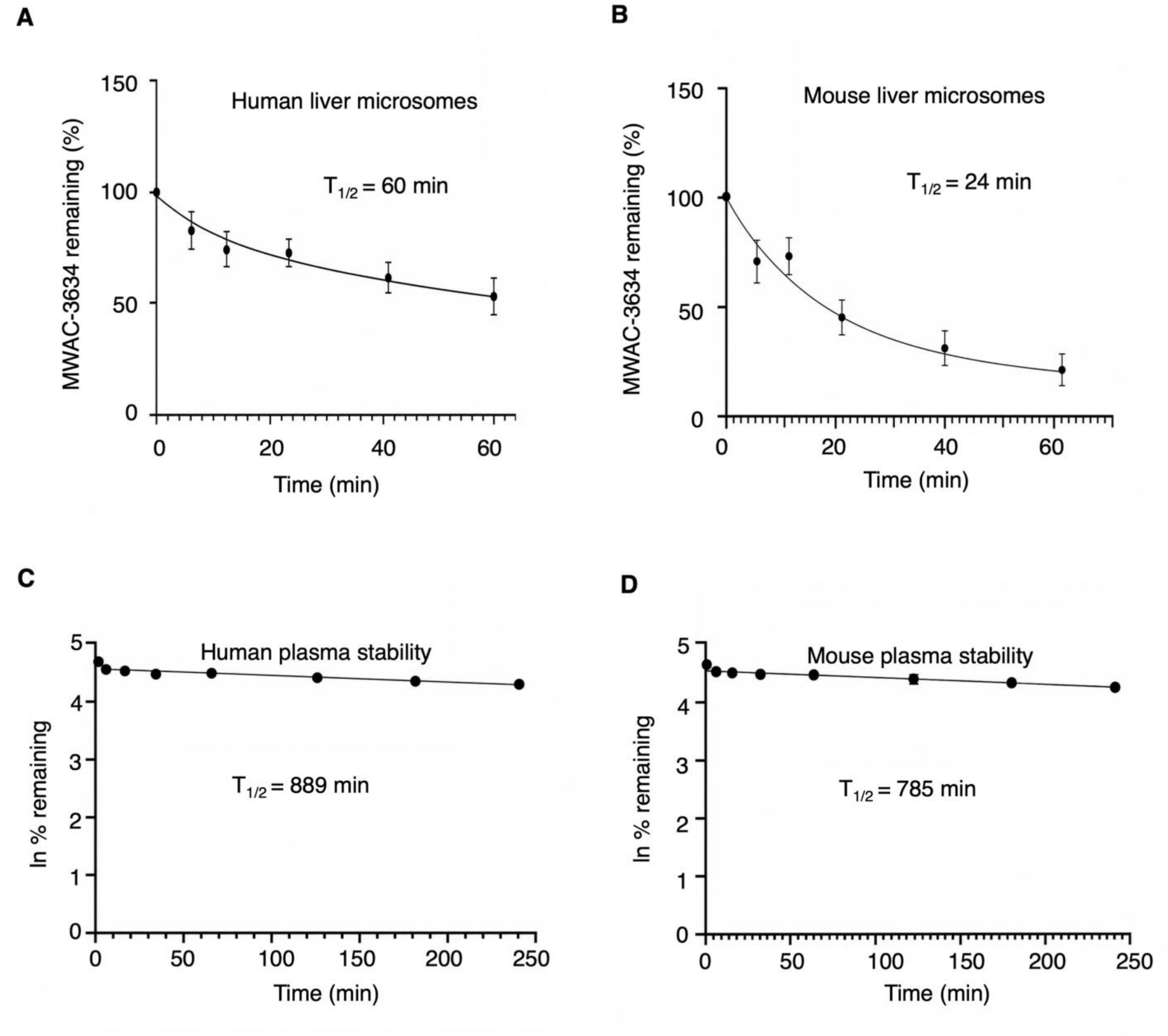


**Figure S7. In vitro metabolic and plasma stability of MWAC-3634. (A and B)** MWAC-3634 was incubated with human **(A)** or mouse **(B)** liver microsomes, and the percentage of compound remaining was measured over time. **(C and D)** MWAC-3634 was incubated in human **(C)** or mouse **(D)** plasma, and the percentage of compound remaining was measured over time. Half-lives are indicated in each panel. Circles indicate mean values, and solid lines indicate fitted curves. In **(A)** and **(B)**, data are presented as mean ± SEM (n = 3 technical replicate incubations per time point). In **(C)** and **(D)**, points represent the mean of n = 2 technical replicate incubations per time point; error bars are not shown because n < 3. $T_{1/2}$, half-life; ln, natural logarithm; SEM, standard error of the mean. Source data are provided as a Source Data file.

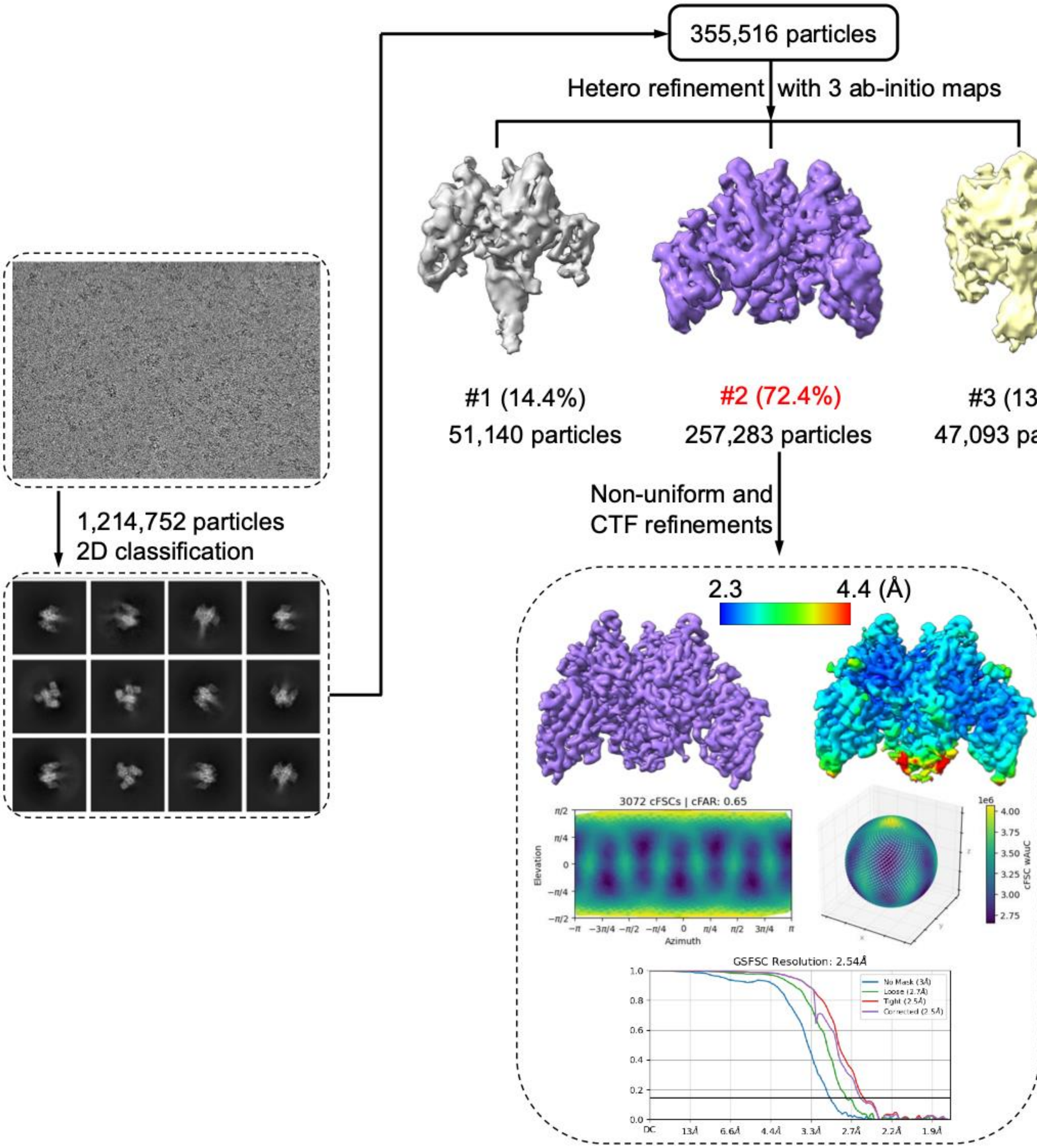


**Figure S8. Cryo-EM image processing and map reconstruction workflow for the ternary complex of EBOV GP ectodomain, MWAC-3634, and Nanosota-EB2.** Representative raw cryo-EM images and 2D class averages of the complex are shown. Representative cryo-EM micrographs were selected from a dataset comprising 6,106 independently acquired images, all of which showed similar particle distributions and yielded a consistent reconstruction. 3D refinement using particles from high-quality 3D classes yielded a 2.54 Å map. The three ab initio class maps are shown in grey, purple, and yellow, respectively. The final maps, half-map FSC curves, angular distribution plot, and corresponding local-resolution maps are shown within the dashed black box. Local-resolution maps are colored according to the indicated scale. Cryo-EM, cryogenic electron microscopy; 2D, two-dimensional; 3D, three-dimensional; CTF, contrast transfer function; FSC, Fourier shell correlation.

**Table S1. Comparison of MWAC-3634 and toremifene for structural contacts, efficacy, and pharmacokinetic properties**

| Parameter | MWAC-3634 | Toremifene |
|---|---|---|
| GP-bound structure used for structural comparison | EBOV GP/Nanosota-EB2/MWAC-3634 cryo-EM structure, PDB 9PEL | EBOV GP/toremifene crystal structure, PDB 5JQ7 |
| Compound-GP interface area (Å²) | 574 | 429 |
| Total compound-contacting GP residues | 18 GP residues | 17 GP residues |
| Hydrophobic contacts | 16 GP residues | 15 GP residues |
| Polar interactions | 2 GP residues | 2 GP residues |
| Contacts in western region of the pocket | 8 GP residues | 13 GP residues |
| Contacts in eastern region of the pocket | 10 GP residues | 4 GP residues |
| GP-binding affinity | $K_d$ = 40.7 nM by SPR | $K_d$ = 16 ± 4 μM, reported from thermal-shift-based binding analysis |
| Antiviral potency against EBOV | $IC_{50}$ = 0.65 nM against authentic wild-type EBOV;<br>$IC_{50}$ = 1.2 nM against Mayinga GP-pseudovirus | $IC_{50}$ values are in the low-micromolar range against EBOV entry or infection, depending on assay |
| Aqueous solubility | 0.08 μM | 0.44 mg/mL has been reported for toremifene citrate |
| Plasma protein binding | 99.96% | >99.5% serum protein binding, mainly albumin |
| In vitro metabolic stability | Human liver microsomal $T_{1/2}$ = 60 min; mouse liver microsomal $T_{1/2}$ = 24 min | Not reported in a directly comparable liver-microsomal stability assay |
| Plasma stability | Human plasma T1/2 = 889 min; mouse plasma $T_{1/2}$ = 785 min | Not reported in a directly comparable human or mouse plasma-stability assay |

| | | |
|---|---|---|
| In vivo half-life in mice | IV $T_{1/2}$ = 2.6 h | Oral mouse PK reported $T_{1/2}$ = 0.5 h at 100 mg/kg and 0.2 h at 200 mg/kg |
| Mouse oral exposure | Oral bioavailability = 69%; oral $C_{max}$ = 16.6 µM after 50 mg/kg oral dosing | Oral mouse PK reported $C_{max}$ = 1.23 µM at 100 mg/kg and 3.19 µM at 200 mg/kg |
| Mouse efficacy dosing regimen | 25 mg/kg by IP or oral administration, twice daily after lethal mouse-adapted EBOV challenge | 60 mg/kg on days 0 and 1, then every other day thereafter for a total of 6 doses, in a lethal EBOV mouse model |
| Mouse efficacy outcome | Improved survival after both oral and IP dosing; IP dosing achieved 100% survival in this study | Reported partial protection in mice; 5 of 10 toremifene-treated mice survived to the end of the observation period in the original study |

Notes: The western and eastern pocket-contact counts follow the orientation shown in Fig. 5C. Compound-GP interface areas were calculated using PDBePISA (https://www.ebi.ac.uk/pdbe/pisa/) with a 1.4 Å probe radius. For MWAC-3634, structural values represent the mean across the three ligand-binding sites in the EBOV GP trimer. PK and efficacy values are not from head-to-head assays.

**Table S2: Cryo-EM data collection, refinement and validation statistics.**

| | EBOV GP ectodomain/ Nanosota-EB2/MWAC-3634 |
|---|---|
| **Data collection and processing** | |
| Magnification | 130,000 |
| Voltage (kV) | 300 |
| Electron exposure (e–/Å$^2$) | 54.5 |
| Defocus range (μm) | -1.0 ~ -2.0 |
| Pixel size (Å) | 0.664 |
| Symmetry imposed | C3 |
| Initial particle images (no.) | 1,214,752 |
| Final particle images (no.) | 257,283 |
| Map resolution (Å) | 2.54 |
| FSC threshold | 0.143 |
| Map resolution range (Å) | 2.3-4.4 |
| | |
| **Refinement** | |
| Initial model used (PDB code) | 9BSV |
| Model resolution (Å) | 2.6 |
| FSC threshold | 0.5 |
| Model resolution range (Å) | 35.6-2.5 |
| Map sharpening *B* factor (Å$^2$) | -97.4 |
| Model composition | |
| Non-hydrogen atoms | 9758 |
| Protein residues | 1218 |
| Ligands | 19 |
| *B* factors (Å$^2$) | |
| Protein | 43.18 |
| Nucleotide | Not applicable |
| Ligand | 30.88 |
| R.m.s. deviations | |
| Bond lengths (Å) | 0.003 |
| Bond angles (°) | 0.650 |
| Validation | |
| MolProbity score | 1.83 |
| Clashscore | 11.83 |
| Poor rotamers (%) | 0.10 |
| Ramachandran plot | |
| Favored (%) | 96.31 |
| Allowed (%) | 3.60 |
| Disallowed (%) | 0.08 |

**Protocol S1: Chemical Synthesis of EBOV GP-Targeting Compounds**
**Synthesis of *rac*-(3R,4S)-*N*-Methyl-4-(3-(trifluoromethyl)phenyl)pyrrolidine-3-carboxamide[a]**

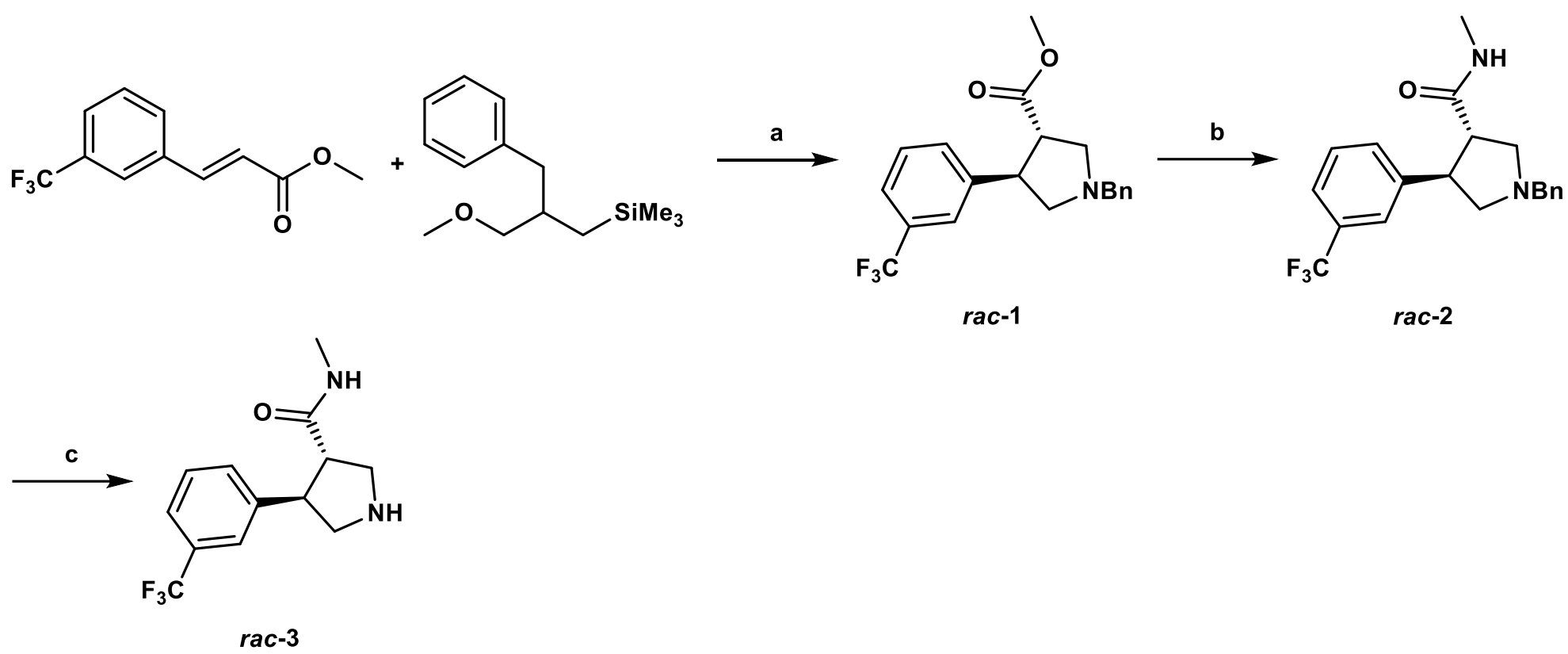


[a]Reagents and Conditions: (a) TFA (0.1 eq), DCM; (b) $MeNH_2$, MeOH, 55 °C; (c) 10% Pd/C, 20% $Pd(OH)_2$, $H_2$, MeOH.

**Synthesis of *rac*-(3R,4S)-*N*-Methyl-4-(3-(trifluoromethyl)phenyl)pyrrolidine-3-carboxamide (*rac*-3)**

**Step 1: *rac*-Methyl (3*R*,4*S*)-1-benzyl-4-(3-(trifluoromethyl)phenyl)pyrrolidine-3-carboxylate (*rac*-1)**

To a solution of methyl (*E*)-3-(3-(trifluoromethyl)phenyl)acrylate (900 mg, 3.91 mmol) and *N*-benzyl-1-methoxy-N-((trimethylsilyl)methyl)methanamine (928 mg, 3.91 mmol) in DCM (25 mL) at 0 °C was added a solution of TFA (44.6 mg, 0.391 mmol) in DCM (5 mL) dropwise. The mixture was stirred at RT overnight and then concentrated and the residue purified by silica gel chromatography (Pet. ether:EtOAc=10:1 to 5:1) to afford the title compound (720 mg, 51%) as a colorless oil. $^1$H NMR (400 MHz, DMSO-*d₆*) δ 7.69 (s, 1H), 7.67 – 7.62 (m, 1H), 7.60 – 7.51 (m, 2H), 7.36 – 7.22 (m, 5H), 3.72 – 3.56 (m, 6H), 3.20 – 3.10 (m, 1H), 3.01 (t, *J* = 9.0 Hz, 1H), 2.93 (t, *J* = 9.2, 7.8 Hz, 1H), 2.83 (dd, *J* = 9.2, 6.4 Hz, 1H), 2.58 (dd, *J* = 9.2, 6.4 Hz, 1H). LC/MS calculated for $C_{20}H_{20}F_3NO_2 + H^+$, 364.14, observed 364.05.

**Step 2: *rac*-(3*R*,4*S*)-1-Benzyl-N-methyl-4-(3-(trifluoromethyl)phenyl)pyrrolidine-3-carboxamide (*rac*-2)**

A solution of ***rac*-1** (200 mg, 0.55 mmol) in 30% $NH_2Me$ in MeOH (10 mL) was heated to 55°C overnight in a sealed tube. The mixture was concentrated to afford the title compound (180 mg, 90%) as a yellow solid. $^1$H NMR (400 MHz, $CDCl_3$) δ 7.57 (s, 1H), 7.48 (t, *J* = 7.2 Hz, 2H), 7.41 (d, *J* = 7.6 Hz, 1H), 7.39

– 7.27 (m, 5H), 3.76 – 3.70 (m, 2H), 3.69 – 3.60 (m, 1H), 3.14 (t, *J* = 8.8 Hz, 1H), 3.01 – 2.89 (m, 2H), 2.88 – 2.81 (m, 1H), 2.78 (d, *J* = 4.8 Hz, 3H), 2.74 – 2.67 (m, 1H). LC/MS calculated for $C_{20}H_{21}F_3N_2O + H^+$, 363.2, observed 363.3.

**Step 3: *rac*-(3*R*,4*S*)-*N*-Methyl-4-(3-(trifluoromethyl)phenyl)pyrrolidine-3-carboxamide (*rac*-3)**

To a solution of ***rac*-2** (180 mg, 0.497 mmol) in MeOH (10 mL) was added 10% Pd/C (100 mg) and 20% $Pd(OH)_2$/C (50 mg) and the mixture was stirred at RT for 2 days under $H_2$ balloon. The mixture was filtered and the filtrate concentrated to afford the title compound (120 mg, 88%) as a yellow oil, which was used directly in the next step. $^1$H NMR (400 MHz, DMSO-*d*$_6$) δ 7.56 – 7.53 (m, 4H), 3.48 (q, *J* = 8.2 Hz, 1H), 3.21 – 3.13 (m, 1H), 2.93 – 2.82 (m, 2H), 2.74 – 2.66 (m, 1H), 2.55 – 2.52 (m, 3H). LC/MS calculated for $C_{13}H_{15}F_3N_2O + H^+$, 273.1, observed 273.0.

**Synthesis of (3*S*,4*R*)-*N*-Methyl-4-(3-(trifluoromethyl)phenyl)pyrrolidine-3-carboxamide[a]**

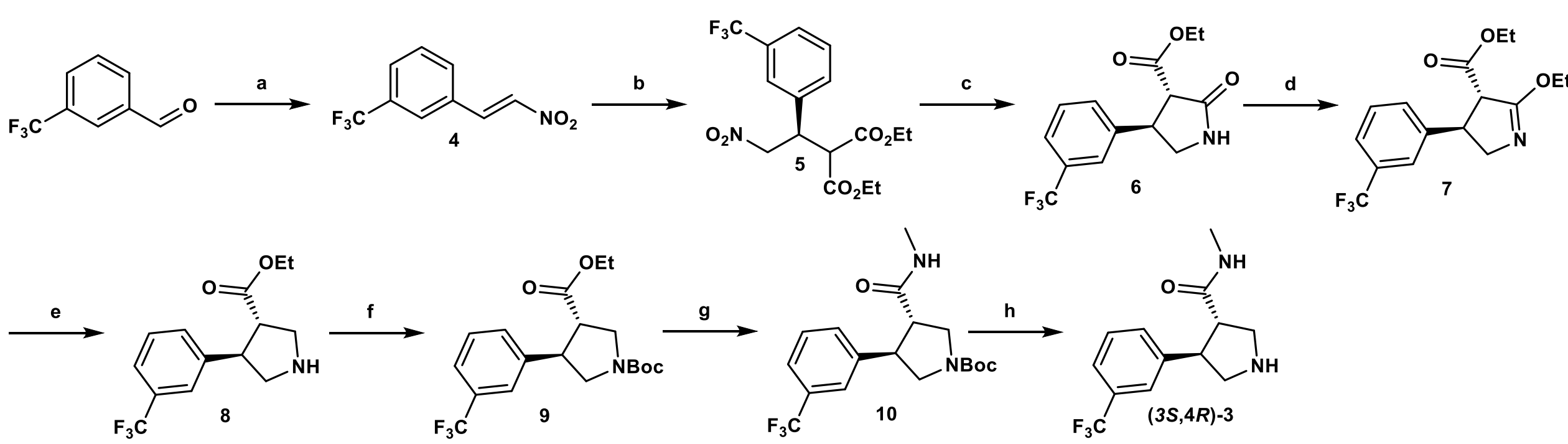


[a]Reagents and Conditions: (a) Ammonium acetate, acetic acid, nitromethane; (b) Ni(II)-Bis[(*S*,*S*)-*N*,*N*′-dibenzylcyclohexane-1,2-diamine]$Br_2$, diethyl malonate, toluene; (c) $NiCl_2 \cdot 6H_2O$, $NaBH_4$, EtOH; (d) $Et_3OBF_4$, DCM; (e) $NaBH_3CN$, 4M HCl in MeOH; (f) $Boc_2O$, DIEA, DCM; (g) $MeNH_2$, MeOH, 55 °C; (h) 4M HCl in dioxane.

**Synthesis of (3*S*,4*R*)-*N*-Methyl-4-(3-(trifluoromethyl)phenyl)pyrrolidine-3-carboxamide (*3S*,*4R*)-3**

**Step 1: (*E*)-1-(2-Nitrovinyl)-3-(trifluoromethyl)benzene (4)**

To a solution of 3-(trifluoromethyl)benzaldehyde (5.0 g, 28.7 mmol) in $MeNO_2$ (15 mL) and acetic acid (30 mL) was added $CH_3COONH_4$ (5.31 g, 68.92 mmol). The mixture was heated to 90 ºC overnight under $N_2$. After cooling to RT, the mixture was diluted with $H_2O$ (80 mL), extracted with DCM (4 × 100 mL) and the combined organic layers were dried over $Na_2SO_4$, filtered and concentrated. The residue was purified by silica gel chromatography (Pet. ether:EtOAc=100:1, v/v) to afford the title compound (2.0 g, 32%) as a green solid. $^1$H NMR (400 MHz, DMSO-$d_6$) δ 8.39 (d, $J$ = 13.6 Hz, 1H), 8.28 (s, 1H), 8.22 (d, $J$ = 13.6 Hz, 1H), 8.15 (d, $J$ = 7.8 Hz, 1H), 7.86 (d, $J$ = 7.8 Hz, 1H), 7.71 (t, $J$ = 7.8 Hz, 1H).

**Step 2: Diethyl (*R*)-2-(2-nitro-1-(3-(trifluoromethyl)phenyl)ethyl)malonate (5)**

To a solution of **4** (1.3 g, 5.99 mmol) and diethyl malonate (1.44 g, 8.98 mmol) in toluene (15 mL) was added Ni(II)-bis[(*S*,*S*)-*N*,*N*'-dibenzylcyclohexane-1,2-diamine]$Br_2$ (96.19 mg, 0.120 mmol) and the mixture was heated to 55 °C overnight. The mixture was diluted with $H_2O$ (40 mL), extracted with DCM (3 × 30 mL) and the combined organic layers were dried over $Na_2SO_4$, filtered and concentrated. The residue was purified by silica gel chromatography on (DCM:MeOH = 100: 1 to 10:1) to afford the title compound (1.9 g, 84%) as a yellow oil. $^1$H NMR (400 MHz, DMSO-$d_6$) δ 7.78 (d, $J$ = 2.2 Hz, 1H), 7.70 – 7.62 (m, 2H), 7.56 (t, $J$ = 7.6 Hz, 1H), 5.09 – 5.04 (m, 2H), 4.23 – 4.09 (m, 4H), 3.83 (q, $J$ = 7.0 Hz, 2H), 1.19 (t, $J$ = 7.0 Hz, 3H), 0.83 (t, $J$ = 7.0 Hz, 3H). LC/MS calculated for $C_{16}H_{18}F_3NO_6 + H^+$, 378.1, observed 378.1.

**Step 3: Ethyl (3*S*,4*R*)-2-oxo-4-(3-(trifluoromethyl)phenyl)pyrrolidine-3-carboxylate (6)**

To a solution of **5** (1.9 g, 5.04 mmol) and $NiCl_2·6H_2O$ (1.44 g, 6.06 mmol) in EtOH (15 mL) at 0°C was added $NaBH_4$ (571 mg, 15.1 mmol) slowly and the mixture was stirred at RT overnight. The mixture was diluted with a saturated aqueous $NH_4Cl$ solution (30 mL) and EtOAc (30 mL) and stirred at RT for 1 h. The layers were separated and the aqueous layer extracted with EtOAc (3 × 30 mL). The combined organic layers were dried over $Na_2SO_4$, filtered and concentrated. The residue was purified by silica gel chromatography (DCM:MeOH = 100:1 to 80:1) to afford the title compound (1.4 g, 92%) as a yellow oil. $^1$H NMR (400 MHz, DMSO-$d_6$) δ 8.16 (s, 1H), 7.73 (s, 1H), 7.69 (d, $J$ = 7.6 Hz, 1H), 7.64 (d, $J$ = 7.8 Hz, 1H), 7.61 – 7.55 (m, 1H), 4.14 – 4.06 (m, 2H), 4.06 – 3.96 (m, 1H), 3.75 (d, $J$ = 11.0 Hz, 1H), 3.69 – 3.63 (m, 1H), 3.27 (t, $J$ = 9.4 Hz, 1H), 1.15 (t, $J$ = 7.0 Hz, 3H). LC/MS calculated for $C_{14}H_{14}F_3NO_3 + H^+$, 302.1, observed 302.0.

**Step 4: Ethyl (3*R*,4*S*)-5-ethoxy-3-(3-(trifluoromethyl)phenyl)-3,4-dihydro-2*H*-pyrrole-4-carboxylate (7)**

To a solution of **6** (1.0 g, 3.3 mmol) in DCM (10 mL) at 0 °C was added $Et_3OBF_4$ (1.26 g, 6.64 mmol) slowly and the mixture was stirred at RT overnight. The reaction was quenched with a saturated aqueous $NaHCO_3$ solution (50 mL) and the mixture extracted with DCM (3 × 30 mL). The combined organic layers were dried over $Na_2SO_4$, filtered and concentrated to give the title compound (1.09 g) as a yellow oil, which was used directly in the next step. LC/MS calculated for $C_{16}H_{18}F_3NO_3 + H^+$, 330.1, observed 330.0.

**Step 5: Ethyl (3*S*,4*R*)-4-(3-(trifluoromethyl)phenyl)pyrrolidine-3-carboxylate (8)**

A mixture of **7** (1.09 g), $NaBH_3CN$ (312 mg, 4.96 mmol) and bromocresol green (23.1 mg, 0.033 mmol) was adjusted to pH 3-4 with a 4 M HCl/MeOH solution and the mixture was stirred at RT for 3 h. The mixture was diluted with $H_2O$ (25 mL), extracted with DCM (4 × 30 mL) and the combined organic layers were dried over $Na_2SO_4$, filtered and concentrated to afford the title compound (860 mg) as a yellow oil, which was used directly in the next step. LCMS: *m/z* 288.100 $[M+H]^+$. LC/MS calculated for $C_{14}H_{16}F_3NO_2 + H^+$, 288.1, observed 288.1.

**Step 6: 1-(*tert*-Butyl) 3-ethyl (3*S*,4*R*)-4-(3-(trifluoromethyl)phenyl)pyrrolidine-1,3-dicarboxylate (9)**

To a solution of **8** (860 mg) and DIEA (1.16 g, 8.98 mmol) in DCM (20 mL) was added $Boc_2O$ (1.03 mL, 4.49 mmol) and the mixture was stirred at RT overnight. The mixture was concentrated and the residue purified by silica gel chromatography (Pet. ether:EtOAc = 20:1 to 10:1) to afford the title compound (460 mg, 35% over 3 steps) as a yellow oil. $^1H$ NMR (400 MHz, DMSO-$d_6$) δ 7.74 – 7.66 (m, 2H), 7.64 – 7.60 (m, 1H), 7.57 (t, $J$ = 7.6 Hz, 1H), 4.06 – 3.90 (m, 2H), 3.83 – 3.72 (m, 2H), 3.69 – 3.58 (m, 1H), 3.50 – 3.40 (m, 2H), 3.31 – 3.26 (m, 1H), 1.41 (d, $J$ = 8.8 Hz, 9H), 1.01 (t, $J$ = 7.0 Hz, 3H).

**Step 7: *tert*-Butyl (3*S*,4*R*)-3-(methylcarbamoyl)-4-(3-(trifluoromethyl)phenyl)pyrrolidine-1-carboxylate (10)**

A solution of **9** (460 mg, 1.19 mmol) in 30% $MeNH_2$ in MeOH (15 mL) was heated at 55 °C overnight in a sealed tube. The mixture was concentrated to afford the title compound (450 mg) as a yellow oil. $^1H$ NMR (400 MHz, DMSO-$d_6$) δ 7.93 (s, 1H), 7.64 – 7.52 (m, 4H), 3.84 – 3.76 (m, 1H), 3.71 – 3.61 (m, 2H), 3.30 – 3.25 (m, 2H), 3.17 – 3.06 (m, 1H), 2.52 (s, 3H), 1.41 (d, $J$ = 5.2 Hz, 9H).

**Step 8: (3*S*,4*R*)-*N*-Methyl-4-(3-(trifluoromethyl)phenyl)pyrrolidine-3-carboxamide hydrochloride (*3S*,*4R*)-3**

A mixture of **10** (50 mg, 0.134 mmol) in 4 M HCl solution in dioxane (4 mL) was stirred at RT for 1 h. The mixture was concentrated to afford the title compound (40 mg). $^1$H NMR (400 MHz, DMSO-$d_6$) δ 8.15 – 8.07 (m, 1H), 7.70 (s, 1H), 7.68 – 7.64 (m, 1H), 7.64 – 7.57 (m, 2H), 3.76 – 3.66 (m, 2H), 3.65 – 3.57 (m, 1H), 3.30 – 3.22 (m, 2H), 3.14 (q, *J* = 8.6 Hz, 1H), 2.53 (s, 3H). LC/MS calculated for $C_{13}H_{15}F_3N_2O + H^+$, 273.1, observed 273.1.

**Synthesis of MWAC-2600 and MWAC-2808[a]**

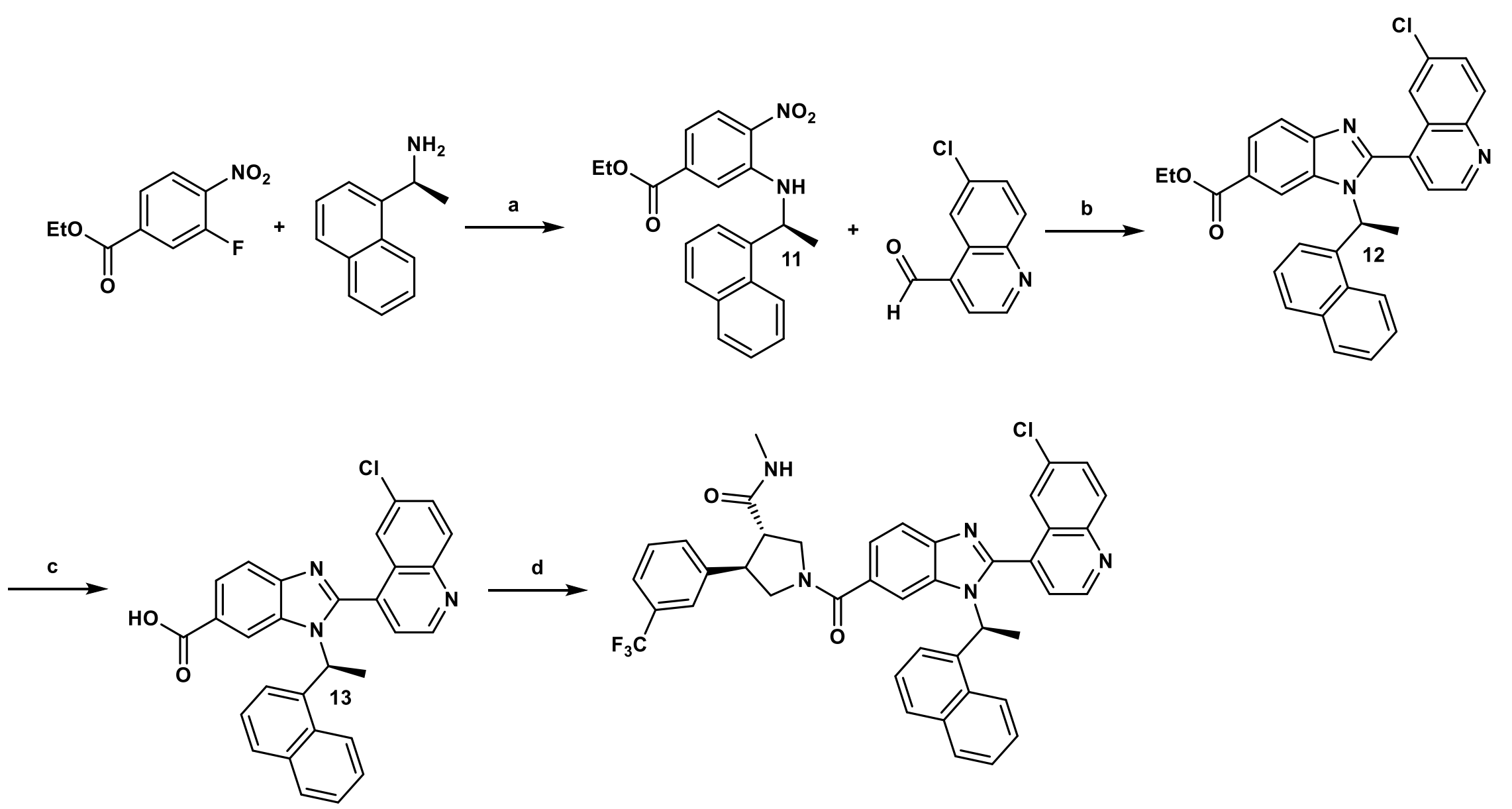


[a]Reagents and Conditions: (a) DIEA, ACN, 80 °C; (b) $Na_2S_2O_4$, DMF, MeOH, 100 ºC; (c) NaOH, THF, MeOH, $H_2O$; (d) HATU, DMF, DIEA, *rac*-**3** (affords MWAC-2600) or **(*3S*,*4R*)-3** (affords MWAC-2808).

**Synthesis of MWAC-2600**

**Step 1: Ethyl (*S*)-3-((1-(naphthalen-1-yl)ethyl)amino)-4-nitrobenzoate (11)**

To a solution of ethyl 3-fluoro-4-nitrobenzoate (1.0 g, 4.69 mmol) and (*S*)-1-(naphthalen-1-yl)ethan-1-amine (1.21 g, 7.04 mmol) in MeCN (10 mL) was added DIEA (3.03 g, 23.5 mmol) and the mixture was heated

to 80°C overnight. After cooling to RT, the mixture was diluted with $H_2O$ (25 mL) and extracted with DCM (4 × 20 mL). The combined organic layers were dried over $Na_2SO_4$, filtered and concentrated. The residue was purified by silica gel chromatography (Pet. ether:EtOAc =20:1 to 15:1) to afford the title compound (1.8 g, ≥99%) as a yellow solid. $^1H$ NMR (400 MHz, DMSO-$d_6$) δ 8.44 – 8.35 (m, 2H), 8.19 (d, *J* = 8.8 Hz, 1H), 7.99 (d, *J* = 8.0 Hz, 1H), 7.84 (d, *J* = 8.2 Hz, 1H), 7.65 (t, 1H), 7.62 – 7.55 (m, 2H), 7.43 (t, *J* = 7.6 Hz, 1H), 7.18 – 7.15 (m, 1H), 7.10 (dd, *J* = 8.8, 1.6 Hz, 1H), 5.74 – 5.68 (m, 1H), 4.01 (q, *J* = 7.0 Hz, 2H), 1.71 (d, *J* = 6.6 Hz, 3H), 0.93 (t, *J* = 7.0 Hz, 3H). LC/MS calculated for $C_{21}H_{20}N_2O_4 + Na^+$, 387.1, observed 387.2.

**Step 2: Ethyl (*S*)-2-(6-chloroquinolin-4-yl)-1-(1-(naphthalen-1-yl)ethyl)-1*H*-benzo[*d*]imidazole-6-carboxylate (12)**

To a solution of **11** (500 mg, 1.37 mmol) and 6-chloroquinoline-4-carbaldehyde (394 mg, 2.06 mmol) in DMF/MeOH (6 mL/2 mL) was added $Na_2S_2O_4$ (1.67 g, 9.60 mmol) and the mixture was heated at 100°C overnight. After cooling to RT, the mixture was diluted with $H_2O$ (25 mL) and extracted with EtOAc (3 × 20 mL). The combined organic layers were washed with brine (3 × 30 mL), dried over $Na_2SO_4$, filtered and concentrated. The residue was purified by prep-TLC (Pet. ether:EtOAc = 1:1, v/v) to afford the title compound (400 mg, 58%) as a yellow solid. $^1H$ NMR (400 MHz, DMSO-$d_6$) δ 8.92 (d, *J* = 4.4 Hz, 1H), 8.63 (s, 1H), 8.00 (d, *J* = 8.2 Hz, 1H), 7.93 (d, *J* = 9.0 Hz, 1H), 7.88 (d, *J* = 8.4 Hz, 1H), 7.68 (d, *J* = 4.4 Hz, 1H), 7.65 – 7.56 (m, 2H), 7.44 – 7.31 (m, 2H), 7.31 – 7.14 (m, 3H), 7.02 – 6.91 (m, 1H), 6.84 – 6.74 (m, 1H), 6.70 – 6.45 (m, 1H), 4.46 – 4.35 (m, 2H), 2.14 (d, *J* = 6.8 Hz, 3H), 1.40 (t, *J* = 7.0 Hz, 3H). LC/MS calculated for $C_{31}H_{24}ClN_3O_2 + H^+$, 506.2, observed 506.3.

**Step 3: (*S*)-2-(6-Chloroquinolin-4-yl)-1-(1-(naphthalen-1-yl)ethyl)-1*H*-benzo[d]imidazole-6-carboxylic acid (13)**

To a solution of **12** (200 mg, 0.395 mmol) in THF/MeOH/$H_2O$ (4 mL/4 mL/2 mL) was added NaOH (47.4 mg, 1.19 mmol) and the mixture was heated to 50°C for 2 h. The mixture was concentrated to remove THF/MeOH and the aqueous residue acidified with 2 M HCl to pH 4-5. The resulting solid was collected by filtration to afford the title compound (150 mg, 79%) as a yellow solid. $^1H$ NMR (400 MHz, DMSO-$d_6$) δ 8.91 (d, *J* = 4.4 Hz, 1H), 8.65 (s, 1H), 8.00 (d, *J* = 8.4 Hz, 1H), 7.92 (d, *J* = 9.0 Hz, 1H), 7.84 (d, *J* = 8.4 Hz, 1H),

7.65 (d, *J* = 4.4 Hz, 1H), 7.59 (t, *J* = 8.8 Hz, 2H), 7.42 – 7.32 (m, 2H), 7.29 (d, *J* = 8.6 Hz, 1H), 7.24 – 7.14 (m, 2H), 6.94 (s, 1H), 6.81 – 6.73 (m, 1H), 6.71 – 6.37 (m, 1H), 2.14 (d, *J* = 6.8 Hz, 3H). LC/MS calculated for $C_{29}H_{20}ClN_3O_2 + H^+$, 478.1, observed 478.2.

**Step 4: [*rac*-(3*R*,4*S*)]-1-(2-(6-Chloroquinolin-4-yl)-1-((*S*)-1-(naphthalen-1-yl)ethyl)-1*H*-benzo[*d*]imidazole-6-carbonyl)-*N*-methyl-4-(3-(trifluoromethyl)phenyl)pyrrolidine-3-carboxamide (MWAC-2600)**

To a solution of **13** (50 mg, 0.105 mmol) and ***rac*-3** (28 mg, 0.105 mmol) in DMF (2 mL) was added HATU (59.7 mg, 0.157 mmol) and DIEA (40.6 mg, 0.314 mmol) and the mixture was stirred at RT overnight. The mixture was diluted with $H_2O$ (20 mL) and extracted with EtOAc (3 × 25 mL). The combined organic layers were washed with brine (3 × 30 mL), dried over $Na_2SO_4$, filtered, and concentrated. The residue was purified by prep-TLC (DCM:MeOH=20:1, v/v) to afford the title compound (35 mg, 46%) as a yellow solid. $^1$H NMR (400 MHz, DMSO-$d_6$) δ 9.01 – 8.88 (m, 1H), 8.29 – 8.07 (m, 1H), 8.05 – 7.77 (m, 3H), 7.74 – 7.49 (m, 9H), 7.43 – 7.24 (m, 3H), 7.22 – 7.14 (m, 1H), 7.10 – 6.84 (m, 2H), 6.71 – 6.56 (m, 1H), 4.21 – 3.58 (m, 6H), 2.58 (d, *J* = 4.4 Hz, 3H), 2.21 – 2.07 (m, 3H). LC/MS calculated for $C_{42}H_{33}ClF_3N_5O_2 + H^+$, 732.2, observed 732.1.

**Synthesis of MWAC-2808**

**Step 1: (3*S*,4*R*)-1-(2-(6-Chloroquinolin-4-yl)-1-((*S*)-1-(naphthalen-1-yl)ethyl)-1*H*-benzo[*d*]imidazole-6-carbonyl)-*N*-methyl-4-(3-(trifluoromethyl)phenyl)pyrrolidine-3-carboxamide (MWAC-2808)**

To a solution of **13** (50 mg, 0.105 mmol), **(*3S*,*4R*)-3** (28 mg, 0.105 mmol) and HATU (59.7 mg, 0.157 mmol) in DMF (3 mL) was added DIEA (40.56 mg, 0.314 mmol). The reaction mixture was stirred at RT for 2 hours. The mixture was diluted with $H_2O$ (20 mL) and extracted with EtOAc (3 × 25 mL). The combined organic layers were dried over $Na_2SO_4$, filtered, and concentrated. The residue was purified by prep-TLC (Gradient:DCM:MeOH=30:1) and prep-HPLC (61% MeCN in $H_2O$ with 0.1% HCOOH) to afford the title compound (14 mg, 18%) as a white solid. $^1$H NMR (400 MHz, DMSO-d$_6$) δ 8.97 (d, *J* = 4.2 Hz, 1H), 8.10 (s, 1H), 8.05 – 7.76 (m, 3H), 7.75 – 7.43 (m, 9H), 7.43 – 7.20 (m, 3H), 7.19 – 6.70 (m, 3H), 6.67 – 6.55 (m, 1H),

4.19 – 3.55 (m, 5H), 3.42 – 3.28 (m, 1H), 2.58 (d, *J* = 4.6 Hz, 3H), 2.16 (s, 3H). LC/MS calculated for $C_{42}H_{33}ClF_3N_5O_2 + H^+$, 732.2, observed 732.1.

**Synthesis of MWAC-3006 and MWAC-3634**[a]

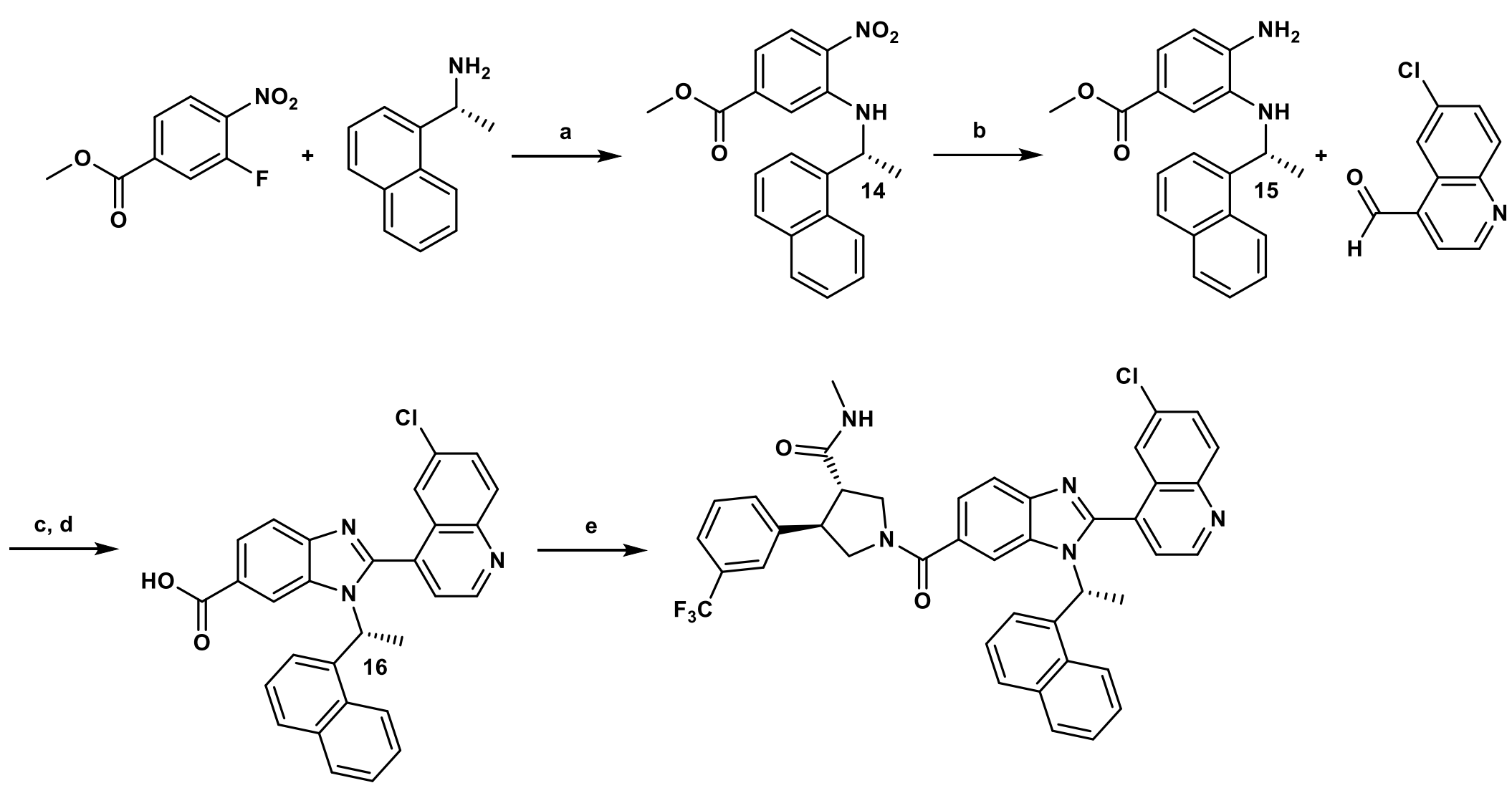


[a]Reagents and Conditions: (a) DIEA, ACN, 80 °C; (b) Fe, $NH_4Cl$, THF, EtOH, $H_2O$, 80 °C; (c) Oxone, DMF; (d) NaOH, THF, MeOH, $H_2O$; (e) HATU, DMF, DIEA, *rac*-**3** (affords **MWAC-3006**) or **(*3S*,*4R*)-3** (affords **MWAC-3634**).

**Synthesis of MWAC-3006**

**Step 1: Methyl (*R*)-3-((1-(naphthalen-1-yl)ethyl)amino)-4-nitrobenzoate (14)**

To a solution of methyl 3-fluoro-4-nitrobenzoate (5.0 g, 25 mmol) and (*R*)-1-(naphthalen-1-yl)ethan-1-amine (4.3 g, 25 mmol) in MeCN (40 mL) was added DIEA (9.74 g, 75.3 mmol) and the mixture was heated at 80 °C overnight. The mixture was concentrated and the residue purified by trituration (DCM/hexane = 3 mL/30 mL) to afford the title compound (9.8 g, ≥99%) as a yellow solid. $^1H$ NMR (400 MHz, DMSO-*d6*) δ 8.40 – 8.36 (m, 2H), 8.19 (d, *J* = 8.8 Hz, 1H), 7.99 (dd, *J* = 8.2, 1.2 Hz, 1H), 7.84 (d, *J* = 8.0 Hz, 1H), 7.69 – 7.64 (m, 1H), 7.62 – 7.56 (m, 2H), 7.44 (t, *J* = 7.6 Hz, 1H), 7.22 (d, *J* = 1.6 Hz, 1H), 7.11 (dd, *J* = 8.8, 1.6 Hz, 1H), 5.74 (p, *J* = 6.4 Hz, 1H), 3.61 (s, 3H), 1.71 (d, *J* = 6.6 Hz, 3H).

**Step 2: Methyl (*R*)-4-amino-3-((1-(naphthalen-1-yl)ethyl)amino)benzoate (15)**

To a solution of **14** (1.38 g, 3.71 mmol) in THF/EtOH/$H_2O$ (15 mL/15 mL/7.5 mL) was added Fe (1.04 g, 18.6 mmol) and $NH_4Cl$ (1.98 g, 37.1 mmol) and the mixture was heated at 80 °C for 4 h. The mixture was filtered and the filtrate concentrated. The residue was diluted with $H_2O$ (30 mL), extracted with DCM (25 mL × 3) and the combined organic phases were dried over $Na_2SO_4$, filtered and concentrated. The residue was triturated with MeOH (10 mL × 2) to afford the title compound (870 mg, 73%) as an off-white solid. $^1$H NMR (400 MHz, DMSO-$d_6$) δ 8.35 (d, $J$ = 8.4 Hz, 1H), 7.95 (d, $J$ = 8.0 Hz, 1H), 7.77 (d, $J$ = 8.0 Hz, 1H), 7.65 – 7.59 (m, 1H), 7.57 – 7.53 (m, 1H), 7.52 – 7.49 (m, 1H), 7.41 (t, $J$ = 7.6 Hz, 1H), 7.03 (dd, $J$ = 8.0, 1.8 Hz, 1H), 6.66 (d, $J$ = 1.8 Hz, 1H), 6.53 (d, $J$ = 8.0 Hz, 1H), 5.65 (s, 2H), 5.35 (p, $J$ = 6.4 Hz, 1H), 5.27 (d, $J$ = 6.0 Hz, 1H), 3.48 (s, 3H), 1.59 (d, $J$ = 6.4 Hz, 3H). LC/MS calculated for $C_{20}H_{20}N_2O_2 + H^+$, 321.2, observed 321.1.

**Step 3: Methyl (*R*)-2-(6-chloroquinolin-4-yl)-1-(1-(naphthalen-1-yl)ethyl)-1*H*-benzo[*d*]imidazole-6-carboxylate**

To a solution of **15** (878 mg, 2.74 mmol) and 6-chloroquinoline-4-carbaldehyde (500 mg, 2.61 mmol) in DMF (10 mL) was added Oxone (587 mg, 1.70 mmol) and the mixture was stirred at RT for 3 h. The mixture was diluted with $H_2O$ (50 mL), extracted with EtOAc (3 × 30 mL) and the combined organic layers washed with brine (3 × 45 mL), dried over $Na_2SO_4$, filtered and concentrated. The residue was purified by silica gel chromatography on (DCM/MeOH = 100/1) and prep-TLC (DCM/MeOH = 25/1) to afford the title compound (1 g, 78%) as a yellow oil. $^1$H NMR (400 MHz, DMSO-$d_6$) δ 8.90 (d, $J$ = 4.4 Hz, 1H), 8.70 (br s, 1H), 8.01 (dd, $J$ = 8.4, 1.4 Hz, 1H), 7.94 – 7.86 (m, 2H), 7.65 (d, $J$ = 4.4 Hz, 1H), 7.58 (t, $J$ = 8.8 Hz, 2H), 7.34 (t, $J$ = 7.4 Hz, 2H), 7.28 (d, $J$ = 8.4 Hz, 1H), 7.24 – 7.13 (m, 2H), 6.97 – 6.88 (m, 1H), 6.81 (q, $J$ = 6.2 Hz, 1H), 6.58 – 6.37 (m, 1H), 3.95 (s, 3H), 2.13 (d, $J$ = 6.8 Hz, 3H). LC/MS calculated for $C_{30}H_{22}ClN_3O_2 + H^+$, 492.14, observed 492.15.

**Step 4: (*R*)-2-(6-Chloroquinolin-4-yl)-1-(1-(naphthalen-1-yl)ethyl)-1*H*-benzo[*d*]imidazole-6-carboxylic acid (16)**

To a solution of methyl (*R*)-2-(6-chloroquinolin-4-yl)-1-(1-(naphthalen-1-yl)ethyl)-1*H*-benzo[d]imidazole-6-carboxylate (1.23 g, 2.50 mmol) in THF/MeOH/$H_2O$ (10 mL/10 mL/5 mL) was added NaOH (200 mg, 5.0 mmol) and the mixture was heated at 50°C for 2 h. The mixture was concentrated to

remove THF/MeOH and the aqueous residue adjusted pH 3-4 with 6 M aqueous HCl. The resulting precipitate was collected by filtration to afford the title compound (860 mg, 72%) as a white solid. $^1$H NMR (400 MHz, DMSO-*d6*) δ 13.0 (s, 1H), 8.91 (d, *J* = 4.4 Hz, 1H), 8.66 (br s, 1H), 7.99 (d, *J* = 8.4 Hz, 1H), 7.92 (d, *J* = 9.0 Hz, 1H), 7.86 (d, *J* = 8.4 Hz, 1H), 7.66 (d, *J* = 4.4 Hz, 1H), 7.59 (t, *J* = 8.6 Hz, 2H), 7.41 – 7.32 (m, 2H), 7.29 (d, *J* = 8.4 Hz, 1H), 7.24 – 7.14 (m, 2H), 7.01 – 6.89 (m, 1H), 6.84 – 6.73 (m, 1H), 6.68 – 6.46 (m, 1H), 2.14 (d, *J* = 6.8 Hz, 3H). LC/MS calculated for $C_{29}H_{20}ClN_3O_2$ + $H^+$, 478.12, observed 478.10.

**Step 5: [*rac*-(3*R*,4*S*)]-1-(2-(6-Chloroquinolin-4-yl)-1-((*R*)-1-(naphthalen-1-yl)ethyl)-1*H*-benzo[*d*]imidazole-6-carbonyl)-*N*-methyl-4-(3-(trifluoromethyl)phenyl)pyrrolidine-3-carboxamide (MWAC-3006)**

A solution of **16** (90 mg, 0.188 mmol), ***rac*-3** (102 mg, 0.376 mmol), HATU (107 mg, 0.282 mmol), DIEA (121 mg, 0.941 mmol) in DMF (3 mL) was stirred at RT overnight. The mixture was poured into water (30 mL) and extracted with EtOAc (20 mL x 3). The combined organic layers were washed with brine (10 mL x 2), dried over $Na_2SO_4$, concentrated and the residue purified by prep-TLC (DCM: MeOH=20:1) to afford the title compound (115 mg, 83%) as a white solid.

$^1$H NMR (400 MHz, DMSO-*d6*) δ 8.99 – 8.90 (m, 1H), 8.29 – 8.08 (m, 1H), 8.05 – 7.77 (m, 3H), 7.75 – 7.52 (m, 8H), 7.47 – 7.12 (m, 4H), 7.11 – 6.83 (m, 2H), 6.79 – 6.56 (m, 1H), 4.24 – 3.91 (m, 1H), 3.90 – 3.58 (m, 4H), 3.43 – 3.21 (m, 1H), 2.62 – 2.52 (m, 3H), 2.22 – 2.07 (m, 3H). LC/MS calculated for $C_{42}H_{33}ClF_3N_5O_2$ + $H^+$, 732.2, observed 732.1.

**Synthesis of MWAC-3634**

**Step 1: (3*S*,4*R*)-1-(2-(6-Chloroquinolin-4-yl)-1-((*R*)-1-(naphthalen-1-yl)ethyl)-1*H*-benzo[*d*]imidazole-6-carbonyl)-*N*-methyl-4-(3-(trifluoromethyl)phenyl)pyrrolidine-3-carboxamide (MWAC-3634)**

To a solution of **16** (250 mg, 0.523 mmol), **(*3S*,*4R*)-3** (171 mg, 0.628 mmol) and HATU (298 mg, 0.785 mmol) in DMF (8 mL) was added DIEA (203 mg, 1.57 mmol) and the mixture was stirred at RT overnight. The mixture was diluted with $H_2O$ (50 mL) and the resulting precipitate collected by filtration and purified by prep-TLC (DCM/MeOH = 20/1) to afford the title compound (304 mg, 79%) as a white solid. $^1$H NMR (400 MHz, DMSO-*d6*) δ 8.93 (d, *J* = 4.2 Hz, 1H), 8.26 (s, 1H), 8.03 – 7.78 (m, 3H), 7.74 – 7.46 (m, 9H), 7.44 – 7.22 (m,

3H), 7.21 – 7.13 (m, 1H), 6.96 – 6.89 (m, 1H), 6.80 – 6.55 (m, 2H), 4.24 – 3.86 (m, 2H), 3.85 – 3.65 (m, 3H), 3.34 – 3.21 (m, 1H), 2.62 – 2.52 (m, 3H), 2.11 (d, *J* = 6.8 Hz, 3H). LC/MS calculated for $C_{42}H_{33}ClF_3N_5O_2 + H^+$, 732.2, observed 732.2.
