# Supplementary material for: A small molecule inhibits Ebola virus entry through glycoprotein stabilization
Source: Nat Commun. 2026 Sep 1;17:10401. doi: 10.1038/s41467-026-77332-3 (PMC13627709; doi:10.1038/s41467-026-77332-3)
Supplement: Supplementary file 2 [file 41467_2026_77332_MOESM2_ESM.pdf]

nature portfolio

Corresponding author(s): Robert Davey, Srinivas Chamakuri, Peter Dosa, Fang Li

Last updated by author(s): Jul 31, 2026

Reporting Summary

Nature Portfolio wishes to improve the reproducibility of the work that we publish. This form provides structure for consistency and transparency in reporting. For further information on Nature Portfolio policies, see our Editorial Policies and the Editorial Policy Checklist.

Statistics

For all statistical analyses, confirm that the following items are present in the figure legend, table legend, main text, or Methods section.

n/a	Confirmed	
☐	☒	The exact sample size (*n*) for each experimental group/condition, given as a discrete number and unit of measurement
☐	☒	A statement on whether measurements were taken from distinct samples or whether the same sample was measured repeatedly
☐	☒	The statistical test(s) used AND whether they are one- or two-sided<br>*Only common tests should be described solely by name; describe more complex techniques in the Methods section.*
☒	☐	A description of all covariates tested
☐	☒	A description of any assumptions or corrections, such as tests of normality and adjustment for multiple comparisons
☐	☒	A full description of the statistical parameters including central tendency (e.g. means) or other basic estimates (e.g. regression coefficient) AND variation (e.g. standard deviation) or associated estimates of uncertainty (e.g. confidence intervals)
☐	☒	For null hypothesis testing, the test statistic (e.g. *F*, *t*, *r*) with confidence intervals, effect sizes, degrees of freedom and *P* value noted<br>*Give P values as exact values whenever suitable.*
☒	☐	For Bayesian analysis, information on the choice of priors and Markov chain Monte Carlo settings
☒	☐	For hierarchical and complex designs, identification of the appropriate level for tests and full reporting of outcomes
☐	☒	Estimates of effect sizes (e.g. Cohen's *d*, Pearson's *r*), indicating how they were calculated

Our web collection on statistics for biologists contains articles on many of the points above.

Software and code

Policy information about availability of computer code

Data collection	Cryo-EM data were collected using EPU version 2.5.<br>SPR data were collected using a Biacore S200.<br>Thermal-shift data were collected using a QuantStudio real-time PCR instrument.<br>Pseudovirus-entry data were collected using an EnSpire plate reader.<br>Authentic-virus imaging data were collected using a Cytation 1 Imaging Reader.
Data analysis	Biacore Evaluation Software (Cytiva); Phoenix WinNonlin (Pharsight Inc.); CellProfiler v4.2.6; cryoSPARC v4.4.1; MotionCor2; CTFFIND-4.1.13; Coot v0.8.9; Phenix v1.16; UCSF ChimeraX v0.93; PyMOL Molecular Graphics System, Version 3.0; LigPlot; GraphPad Prism v10.3.0.

For manuscripts utilizing custom algorithms or software that are central to the research but not yet described in published literature, software must be made available to editors and reviewers. We strongly encourage code deposition in a community repository (e.g. GitHub). See the Nature Portfolio guidelines for submitting code & software for further information.

Data

Policy information about availability of data

All manuscripts must include a data availability statement. This statement should provide the following information, where applicable:

- Accession codes, unique identifiers, or web links for publicly available datasets
- A description of any restrictions on data availability
- For clinical datasets or third party data, please ensure that the statement adheres to our policy

The atomic model and corresponding cryo-EM density map of the ternary complex comprising EBOV GP, MWAC-3634, and Nanosota-EB2 have been deposited in the Protein Data Bank and the Electron Microscopy Data Bank, respectively, under accession numbers 9PEL and EMD-71566.
Source data are provided with this paper.

Research involving human participants, their data, or biological material

Policy information about studies with human participants or human data. See also policy information about sex, gender (identity/presentation), and sexual orientation and race, ethnicity and racism.

Reporting on sex and gender	No human participants were used in this study.
Reporting on race, ethnicity, or other socially relevant groupings	No human participants were used in this study.
Population characteristics	No human participants were used in this study.
Recruitment	No human participants were used in this study.
Ethics oversight	No human participants were used in this study.

Note that full information on the approval of the study protocol must also be provided in the manuscript.

Field-specific reporting

Please select the one below that is the best fit for your research. If you are not sure, read the appropriate sections before making your selection.

☒ Life sciences ☐ Behavioural & social sciences ☐ Ecological, evolutionary & environmental sciences

For a reference copy of the document with all sections, see nature.com/documents/nr-reporting-summary-flat.pdf

Life sciences study design

All studies must disclose on these points even when the disclosure is negative.

Sample size	No statistical method was used to predetermine sample size. Sample sizes were selected based on technical feasibility and prior experience with these assays.
Data exclusions	No data were excluded from the analysis.
Replication	Numbers and types of biological and technical replicates are stated in the figure legends. The mouse efficacy and pharmacokinetic studies were each performed once.
Randomization	For in vivo testing, BALB/cJ mice were randomized into groups of 10 with equal numbers of males and females. For pharmacokinetic testing, female C57BL/6J mice were used.
Blinding	Investigators were not blinded to treatment allocation in the mouse study. Survival and body weight were objectively recorded, and serum viral load was quantified by RT-qPCR. Clinical scores were assigned using predefined scoring criteria.

Reporting for specific materials, systems and methods

We require information from authors about some types of materials, experimental systems and methods used in many studies. Here, indicate whether each material, system or method listed is relevant to your study. If you are not sure if a list item applies to your research, read the appropriate section before selecting a response.

Materials & experimental systems

n/a	Involved in the study
☐	☒ Antibodies
☐	☒ Eukaryotic cell lines
☒	☐ Palaeontology and archaeology
☐	☒ Animals and other organisms
☒	☐ Clinical data
☒	☐ Dual use research of concern
☒	☐ Plants

Methods

n/a	Involved in the study
☒	☐ ChIP-seq
☒	☐ Flow cytometry
☒	☐ MRI-based neuroimaging

Antibodies

Antibodies used	EBOV GP mAb 4F3 (IBT, 0201-020)
Validation	The antibody were purchased from IBT BIOSERVICES. It was validated by the company as shown on their website. https://ibtbioservices.com/shop/primary-antibodies/mouse-anti-ebov-gp-mab-4f3/

Eukaryotic cell lines

Policy information about cell lines and Sex and Gender in Research

Cell line source(s)	HEK293T (American Type Culture Collection, ATCC, CRL-3216), HeLa cells (ATCC, CCL-2), Expi293F cells (Thermo Fisher, A14527), Huh-7 cells (Applied Biological Materials, T8973).
Authentication	Cell lines were obtained from commercial vendors that authenticate their cell lines; they were not independently authenticated in our laboratories.
Mycoplasma contamination	Cell lines were tested negative for mycoplasma contamination by vendors.
Commonly misidentified lines (See ICLAC register)	None.

Animals and other research organisms

Policy information about studies involving animals; ARRIVE guidelines recommended for reporting animal research, and Sex and Gender in Research

Laboratory animals	Female C57BL/6J mice (16 weeks old; Jackson Laboratory); BALB/cJ mice (5-8 weeks old; Jackson Laboratory).
Wild animals	The study did not involve wild animals.
Reporting on sex	For in vivo efficacy testing, mice were randomized into groups of 10 with equal numbers of males and females. For pharmacokinetic testing, female C57BL/6J mice were used. The study was not designed to assess sex-specific effects.
Field-collected samples	The study did not involve samples collected from field.
Ethics oversight	This study was performed in strict accordance with the recommendations in the Guide for the Care and Use of Laboratory Animals of the National Institutes of Health. All of the animals were handled according to approved institutional animal care and use committee (IACUC) protocols at Boston University (protocol number: 201900062) and at the University of Florida Scripps Biomedical Research (protocol number: 15-022-04).

Note that full information on the approval of the study protocol must also be provided in the manuscript.

Plants

Seed stocks	No plants were used in this study.
Novel plant genotypes	No plants were used in this study.
Authentication	No plants were used in this study.
