# Supplementary material for: A small molecule inhibits Ebola virus entry through glycoprotein stabilization
Source: Nat Commun. 2026 Sep 1;17:10401. doi: 10.1038/s41467-026-77332-3 (PMC13627709; doi:10.1038/s41467-026-77332-3)
Supplement: Supplementary file 3 [file 41467_2026_77332_MOESM3_ESM.pdf]

nature portfolio

Peer Review File

A small molecule inhibits Ebola virus entry through glycoprotein stabilization

Corresponding Author: Professor Fang Li

This file contains all reviewer reports in order by version, followed by all author rebuttals in order by version.

Version 0:

Reviewer comments:

Reviewer #1

(Remarks to the Author)
The manuscript "A small molecule inhibits Ebola virus entry through glycoprotein stabilization" by Bu et al. describes the identification and testing of an Ebola virus (EBOV) inhibitor both in vitro and in vivo. The authors used a DNA-encoded chemical library featuring 4.7 billion compounds to screen for GP binders, with follow-up and compound optimization in retroviral pseudotype assays. Top candidates were then evaluated in vitro using infectious EBOV, and in vivo using mouse-adapted EBOV. In the mouse model authors observe full protection after i.p. treatment with MWAC-3634, and, importantly, partial protection after oral treatment, making this compound very interesting for potential use in the field, as currently available treatments (with the notable exception of obeldesivir, although here only preclinical data are currently available) require i.v. administration, which is highly challenging during outbreaks. Additionally, the authors performed mechanistic work, demonstrating that the mechanism of action of MWAC-3634 is most likely by stabilizing GP in its prefusion conformation, which represents (to the best of my knowledge) a novel mechanism of action against EBOV entry, and is a significant strength of the manuscript.

The manuscript is well-structured and easy to follow. However, experimental details should sometimes be better described, primary data are sometimes missing, and some of the references in the introduction should have been picked with greater care. Experiments are well-executed and appropriate for the conclusions drawn. Unfortunately, in their discussion and introduction the authors often tend to represent already existing countermeasures in an overly negative fashion. They also directly comment on the quality of their own study (e.g. "Our structural study […] represents a major breakthrough"), which in my view seems inappropriate for a scientific paper. While I understand the motivation for this, it becomes problematic when the current state of the field is presented in a distorted fashion. Importantly, I don't think the authors would have to resort to diminishing other researchers' work, as their own work clearly has merit. However, these issues can be corrected with relatively minor changes to the text (easiest would be to just omit these statements).

The other major potential issue I see with this study is with respect to the specificity of the drug. A single point mutation in GP significantly reduces its in vitro activity, and there is only residual activity against other filovirus species, even the relatively closely related Sudan virus. To be fair, the currently approved antibody treatments are also species specific; however, within a species they are protective against a wide range of different strains/isolates, and based on the presented data it is not clear whether this would be the case for MWAC-3634. This is something that needs to be addressed experimentally, and that can be easily done by the authors with assays available to them.

Overall, while the work clearly has merit, the authors should address the following comments:

Major comments:

1) Figure 1 and 2A: While the authors show IC50 and CC50 values, they do not show the actual data underlying these calculations. These data should be added in one or several supplemental figure(s).

2) Figure 2B: The same is true for the microsomal half-life and plasma stability values – primary data need to be presented as supplemental figure.

3) One major potential weakness is a possible strain specificity, since already a single point mutation in GP significantly

affects the IC50 of the drug. While the authors checked efficacy against the Mayinga isolate, they should compare this against more contemporary isolates such as Makona and/or Kikwit. This should be done at least in the pseudotype assay, although doing so with infectious EBOV would be the more informative experiment. The resulting data should be included in the manuscript, and the authors should discuss their implications for the practical usability of MWAC-3634.

Minor comments:

4) Line 56: “The sole FDA-approved vaccine and two 56 FDA-approved human antibody therapies reduce case fatality to ~25% and ~35%, respectively [6-8].“ This statement misrepresents the benefits that these countermeasures have provided since they have become available, particularly in context of the implied comparison with the overall case fatality rate (CFR) of EBOV, which is given as 40% a few lines above.

In case of countermeasures, this comparison is misleading as treatment efforts are limited to severe cases where the CFR is much higher than the overall CFR of 40% (which is mainly driven by the West African outbreak, where massive diagnostic efforts revealed many milder or even asymptomatic cases that would not be included in studies of EBOV countermeasures). By omitting this information the authors generate false expectations that then undermine the efficacy of existing therapies. Further, the references supporting this statement are poorly chosen: One of them (REF [7]) is a brief review that actually provides no CFRs after treatment at all, whereas the other (REF [8]) is a review which reports the results of the PALM trial. Importantly, while this trial represents an important milestone in the development of EBOV countermeasures, it did not include a standard-of-care/no-specific-treatment control group, but rather compared 4 different specific treatments. Maybe a better reference would be PMID 40375337, which is an analysis of data from the 2018-2020 outbreak in DRC and includes a standard-of-care/no-specific-treatment group with a CFR of 89%, compared to CFRs of 30% and 32% for the Ebanga/Mab114 and Inmazeb/REGN-EB3 treatment groups, respectively.

With respect to vaccines, a reduction in CFR is actually not the main benchmark, as Ervebo/rVSVΔG-ZEBOV-GP is used during outbreaks in form of ring vaccinations in order to limit spread of disease, and has been shown to have a very high efficacy for this (PMID 28017403). In contrast, the study the authors cite (REF[6]) looks at benefits of vaccination in persons with confirmed EBOV diagnosis, and in the majority of these patients the time between vaccination and onset of symptoms was less than 10 days, leaving insufficient time to establish a protective adaptive immunity. Nevertheless, the study clearly showed a) a benefit on CFR even if vaccination occurred 2 or less days prior to onset of symptoms (27%) compared to the control group (52%), and a further reduction in this CFR with increased time between vaccination and onset of symptoms. As a side note, the statement also completely fails to mention the second EBOV vaccine (Zabdeno/MVAbea) that had regulatory approval (albeit only by the EMA but not the FDA), although this approval was recently withdrawn on request of the manufacturer for commercial reasons.

Given that many of the readers of Nature Communications might not be familiar with the intricacies of EBOV countermeasures, this statement by the authors can result in serious misinformation, and I would urge them to rephrase it to represent the current state-of-the-art in a factually accurate and neutral way. Alternatively, it might be best to omit statements about the efficacy of previous countermeasures completely, and instead focus on issues with respect to requirements for storage and delivery route, which is where I see the main potential benefits of MWAC-3634.

5) line 60: “although none has been approved by the FDA” – To what extent is FDA approval relevant for a disease that is not endemic in the US? Consider omitting this statement.

6) Line 69: The authors should reconsider their use of abbreviations, particularly taking into consideration the wider audience of NComms. It might be better to avoid abbreviations such as RBS, MLD, and IFL, to improve accessibility of the manuscript and to avoid jargon.

7) Similarly, some abbreviations such as “DFF”, “CTF” are never defined. Others (e.g. “PO”, “DSF”) are defined but then never used again.

8) Line 88: “To date, no experimental high-throughput screen has directly targeted EBOV GP, owing to the lack of suitable GP based assays.” There have indeed been screens relying on GP as the sole EBOV protein present in the assay, e.g. PMID 21270170, 33705865, to name just a few. I would recommend omitting this sentence.

9) Line 151: Why do the authors check activity against hERG ion channel? They should provide a rationale for this experiment.

10) Line 190 – 195: Given that the authors report the structural data at a later point in the manuscript, they should consider whether it makes sense to move this section down so that their conclusion comes after the presentation of the results supporting it.

11) Line 239: Is the use of “western portion/region” and “eastern portion/region” commonly used when talking about protein structures?

12) Line 246: The authors should justify their decision to use a non-standard mouse-adapted strain with just 1 of 3 GP mutations included. Why did they retain the S246P mutation, but reverted the mutation at position 65 and 544? How does this affect the stringency of their animal model?

13) The authors should comment on the location of S246 with relation to the binding pocket of MWAC-3634. Do they, based on the available structural information (maybe in conjunction with modelling), expect an impact of the S246P mutation on this pocket?

14) Line 296: “Our structural study of the EBOV GP ectodomain in complex with MWAC-3634 represents a major breakthrough” – I would recommend the authors leave it up to the readers to make such quality judgements about their work. Alternatively, they would have to provide compelling arguments for why they consider their study to represent such a breakthrough.

15) Line 308: “Our published structures of SUDV and MARV GPs in complex with NPC1 indicate that this pocket is relatively conserved with respect to EBOV. Consistent with this structural conservation, MWAC-3634 retains measurable activity against both SUDV and MARV pseudoviruses, albeit at substantially reduced potency.” Given the authors’ access to these structural information, can they model MWAC-3634 in complex with SUDV GP or MARV GP, and by doing so provide an explanation for the reduced potency?

16) Line 313: “suggest that structure-guided adaptation of this scaffold may enable broader-spectrum GP-stabilizing anti-filovirus inhibitors.“ Given that the current data indicate a rather narrow specificity of MWAC-3634, I would consider this statement rather speculative. Can the authors provide some arguments why they think this pocket or the mechanism of GP stabilization might be amenable to broad spectrum inhibition? The fact that they observe apparent conservation of the pocket when comparing it to SUDV/MARV doesn’t really hold, as MWAX-3634 acts comparatively poorly against those viruses. Otherwise I would recommend omitting or rephrasing this statement.

17) Line 335: “our study offers new insights into the mechanisms of EBOV entry and evolution”. Please specify what these new insights are, or omit the sentence.

18) Methodological details appear to be missing, e.g. descriptions of assays to determine microsomal half-life or plasma stability (reported in Fig. 2B).

19) Figure 2A: What do logP and tPSAS stand for? Units (Da, nm²?) appear to be missing.

20) Figure 3: GP-ΔM is not defined.

21) Figure 3: Colours are not explained

Reviewer #2

(Remarks to the Author)
This study reports the discovery of MWAC-3634, a potent small molecule inhibitor of EBOV GP that appears to function through GP stabilization. The use of DEC Tec screening, cryo EM structure determination, and in vivo efficacy in a mouse model is impressive. However, several significant issues need to be addressed to strengthen the mechanistic conclusions, improve data transparency, and better contextualize the compound’s limitations. The following points should be addressed through moderate revision.

1. The main claim of “GP stabilization” relies on DSF thermal shifts and the cryo EM structure. No orthogonal biophysical or functional assay is provided to confirm that MWAC 3634 indeed raises the kinetic barrier to GP2 refolding. Please add at least one independent method to support the stabilization model.

2. MWAC 3634 has very low aqueous solubility, high plasma protein binding, and short mouse microsomal half life. Although oral efficacy was observed, these properties are far from desirable. The authors should provide a critical discussion of whether this scaffold can be further optimized or whether the current results mainly serve as a mechanistic probe.

3. The comparison with toremifene is qualitative. Please provide a supplementary table listing the buried surface area (BSA) and number of hydrophobic/hydrogen bond contacts for MWAC 3634 versus toremifene from the superimposed structures. This would objectively support the claim that MWAC 3634 engages more extensively with the eastern region of the pocket.

4. Throughout the manuscript, units and numbers sometimes lack a space (e.g., “0.65nM”).

Reviewer #3

(Remarks to the Author)
I co-reviewed this manuscript with one of the reviewers who provided the listed reports. This is part of the Nature Communications initiative to facilitate training in peer review and to provide appropriate recognition for Early Career Researchers who co-review manuscripts.

Reviewer #4

(Remarks to the Author)
Comments:
In this manuscript, Bu et al. screened 4.73 billion compounds from DNA-encoded chemical libraries against EBOV GP and identified MWAC-3634 after stereochemical optimization. This compound binds to GP with high affinity (Kd = 40.7 nM), can potently neutralize the infection (IC_{50} = 0.65 nM), shows no detectable cytotoxicity, and has favorable pharmacokinetics in mice.
Moreover, they solved the cryo-EM structure of a ternary complex consisting of EBOV GP ectodomain, MWAC-3006, and Nanosota-EB2 at 2.54 Å resolution, revealing MWAC-3634's unique mechanism: occupation of a hydrophobic cavity at the GP1-GP2 interface that stabilizes the glycoprotein—a distinct approach compared to GP destabilizing inhibitors. In lethal-challenge models, both intraperitoneal and oral administration significantly increased the survival rates, while reducing weight loss and the severity of clinical diseases. In summary, this study demonstrates the effectiveness of DNA-encoded chemical libraries in anti-filovirus drug discovery, identifies GP stabilization as a promising anti-filovirus strategy, and highlights MWAC-3634 as a highly potent and promising lead compound for future therapeutic development.

Specific comments:
1.Please compare the efficacy and pharmacokinetic properties (including half-life, solubility, and and dosing regimen in mice) of the small-molecule inhibitor MWAC-3634 with those of previously reported small-molecule inhibitors. This comparison will provide a clear and intuitive assessment of the advantages and disadvantages of MWAC-3634.

2.The authors propose that MWAC-3634 functions by stabilizing GP in its prefusion conformation, representing a novel mechanism distinct from previously reported small-molecule inhibitors. However, during structural determination of the MWAC-3634-GP binary complex, the flexibility of GP made it challenging to unambiguously identify the density corresponding to MWAC-3634. To address this, the authors introduced a nanobody Nanosota-EB2 to stabilize the GP conformation, enabling successful determination of the GP-EB2-MWAC-3634 ternary complex structure where MWAC-3634 density could be clearly resolved. Interestingly, the previously reported structures of GP-destabilizing small molecules were directly determined through binary complex. To further validate the proposed stabilization mechanism of MWAC-3634, it would be valuable for the authors to provide additional experimental evidence demonstrating that MWAC-3634 indeed maintains GP in its prefusion state.

Version 1:

Reviewer comments:

Reviewer #1

(Remarks to the Author)
The authors have adequately addressed my concerns.

Reviewer #2

(Remarks to the Author)
The authors have addressed previous concerns and current version is recommended to publish at nature communication

Reviewer #3

(Remarks to the Author)
I co-reviewed this manuscript with one of the reviewers who provided the listed reports. This is part of the Nature Communications initiative to facilitate training in peer review and to provide appropriate recognition for Early Career Researchers who co-review manuscripts.

Reviewer #4

(Remarks to the Author)
The authors have adequately addressed all my concerns, and the revised results are robust and well-supported. Therefore, I recommend this manuscript for publication.

Open Access This Peer Review File is licensed under a Creative Commons Attribution 4.0 International License, which permits use, sharing, adaptation, distribution and reproduction in any medium or format, as long as you give appropriate credit to the original author(s) and the source, provide a link to the Creative Commons license, and indicate if changes were made.
In cases where reviewers are anonymous, credit should be given to 'Anonymous Referee' and the source.
The images or other third party material in this Peer Review File are included in the article's Creative Commons license, unless indicated otherwise in a credit line to the material. If material is not included in the article's Creative Commons license and your intended use is not permitted by statutory regulation or exceeds the permitted use, you will need to obtain permission directly from the copyright holder.
To view a copy of this license, visit https://creativecommons.org/licenses/by/4.0/

Responses to Reviewers' comments:

Reviewer 1

(1) Review: Figure 1 and 2A: While the authors show IC50 and CC50 values, they do not show the actual data underlying these calculations. These data should be added in one or several supplemental figure(s).

Response: We agree and have added the primary IC_{50} and CC_{50} data to the revised Supplementary Information. The EBOV pseudovirus dose-response curves are now shown in Supplementary Fig. S3, and the corresponding cell-viability data are shown in Supplementary Fig. S4.

(2) Review: Figure 2B: The same is true for the microsomal half-life and plasma stability values – primary data need to be presented as supplemental figure.

Response: We agree and have added the primary data underlying the microsomal half-life and plasma-stability values to the revised Supplementary Information. These data are now shown in Supplementary Fig. S7.

(3) Review: One major potential weakness is a possible strain specificity, since already a single point mutation in GP significantly affects the IC50 of the drug. While the authors checked efficacy against the Mayinga isolate, they should compare this against more contemporary isolates such as Makona and/or Kikwit. This should be done at least in the pseudotype assay, although doing so with infectious EBOV would be the more informative experiment. The resulting data should be included in the manuscript, and the authors should discuss their implications for the practical usability of MWAC-3634.

Response: We addressed this concern by testing MWAC-3634 against EBOV GP-pseudoviruses representing multiple EBOV strains, including Mayinga, Ituri, Mbandaka, and Makona. MWAC-3634 retained strong activity across all tested strains, with IC50 values of 1.2 nM, 0.83 nM, 0.63 nM, and 1.8 nM, respectively. These data are now included in Fig. S5A and described in the Results. We have also noted in the Results that MWAC-3634 shows strong activity across the tested EBOV strains.

(4) Review: Line 56: "The sole FDA-approved vaccine and two 56 FDA-approved human antibody therapies reduce case fatality to ~25% and ~35%, respectively [6-8]." This statement misrepresents the benefits that these countermeasures have provided since they have become available, particularly in context of the implied comparison with the overall case fatality rate (CFR) of EBOV, which is given as 40% a few lines above.

In case of countermeasures, this comparison is misleading as treatment efforts are limited to severe cases where the CFR is much higher than the overall CFR of 40% (which is mainly driven by the West African outbreak, where massive diagnostic efforts revealed many milder or even asymptomatic cases that would not be included in studies of EBOV countermeasures). By omitting this information the authors generate false expectations that then undermine the efficacy of existing therapies. Further, the references supporting this statement are poorly chosen: One of them (REF [7]) is a brief review that actually provides no CFRs after treatment at all, whereas the other (REF [8]) is a review which reports the results of the PALM trial. Importantly, while this trial represents an important milestone in the development of EBOV countermeasures, it did not include a standard-of-care/no-specific-treatment control group, but rather compared 4 different specific treatments. Maybe a better reference would be PMID 40375337, which is an analysis of data from the 2018-2020 outbreak in DRC and includes a standard-of-care/no-specific-treatment group with a CFR of 89%, compared to CFRs of 30% and 32% for the Ebanga/Mab114 and Inmazeb/REGN-EB3 treatment groups, respectively.

With respect to vaccines, a reduction in CFR is actually not the main benchmark, as Ervebo/rVSVΔG-ZEBOV-GP is used during outbreaks in form of ring vaccinations in order to limit spread of disease, and has been shown to have a very high efficacy for this (PMID 28017403). In contrast, the study the authors cite (REF[6]) looks at benefits of vaccination in persons with confirmed EBOV diagnosis, and in the majority of these patients the time between vaccination and onset of symptoms was less than 10 days, leaving insufficient time to establish a

protective adaptive immunity. Nevertheless, the study clearly showed a) a benefit on CFR even if vaccination occurred 2 or less days prior to onset of symptoms (27%) compared to the control group (52%), and a further reduction in this CFR with increased time between vaccination and onset of symptoms. As a side note, the statement also completely fails to mention the second EBOV vaccine (Zabdeno/MVAbea) that had regulatory approval (albeit only by the EMA but not the FDA), although this approval was recently withdrawn on request of the manufacturer for commercial reasons.

Given that many of the readers of Nature Communications might not be familiar with the intricacies of EBOV countermeasures, this statement by the authors can result in serious misinformation, and I would urge them to rephrase it to represent the current state-of-the-art in a factually accurate and neutral way. Alternatively, it might be best to omit statements about the efficacy of previous countermeasures completely, and instead focus on issues with respect to requirements for storage and delivery route, which is where I see the main potential benefits of MWAC-3634.

Response: We thank the reviewer for this important clarification regarding EBOV countermeasures. We have removed the previous statement about case-fatality rates and replaced it with: "FDA-approved vaccines and antibody therapies have improved prevention and treatment of EBOV disease." We have also updated the supporting references as recommended.

(5) Review: line 60: "although none has been approved by the FDA" – To what extent is FDA approval relevant for a disease that is not endemic in the US? Consider omitting this statement.

Response: This statement has been removed.

(6) Review: Line 69: The authors should reconsider their use of abbreviations, particularly taking into consideration the wider audience of NComms. It might be better to avoid abbreviations such as RBS, MLD, and IFL, to improve accessibility of the manuscript and to avoid jargon.

Response: We agree and have revised the manuscript to improve accessibility for a broad Nature Communications readership. In the revised manuscript, we now spell out "receptor-binding site" and "mucin-like domain," because these terms appeared only a limited number of times. We have retained the abbreviation "IFL" after defining it at first use as "internal fusion loop," because this term appears more frequently in the structural and mechanistic sections, where repeated spelling-out would reduce readability and concision.

(7) Review: Similarly, some abbreviations such as "DFF", "CTF" are never defined. Others (e.g. "PO", "DSF") are defined but then never used again.

Response: We have revised the manuscript and supplementary materials to improve abbreviation use and consistency.

The DFF lid was defined in the original manuscript, but we have revised the wording to make the definition clearer. The revised text now reads: "This pocket is occupied and sealed by the DFF lid, named for its component residues Asp192 (D), Phe193 (F), and Phe194 (F); this lid stabilizes the GP structure (Fig. 4D)."

We have also defined CTF as "contrast transfer function" at first use.

The abbreviations "PO" and "DSF" have been removed, and the corresponding terms are now written as "oral administration" and "differential scanning fluorimetry," respectively.

(8) Review: Line 88: "To date, no experimental high-throughput screen has directly targeted EBOV GP, owing to the lack of suitable GP based assays." There have indeed been screens relying on GP as the sole EBOV

protein present in the assay, e.g. PMID 21270170, 33705865, to name just a few. I would recommend omitting this sentence.

Response: The two cited studies are GP-dependent or GP-directed discovery efforts, but they differ from the specific point we intended to make. PMID 21270170 used a pseudovirus-based screening assay, and PMID 33705865 used a GP-directed in silico screening approach. Neither study used purified EBOV GP as the direct target in an experimental high-throughput screen. To avoid ambiguity, we have revised the sentence as follows:

"To our knowledge, no prior experimental high-throughput screen has directly used purified EBOV GP as the target, likely reflecting the difficulty of adapting purified GP-based binding assays to conventional high-throughput screening formats."

(9) Review: Line 151: Why do the authors check activity against hERG ion channel? They should provide a rationale for this experiment.

Response: We have removed the hERG ion-channel assay from the revised manuscript to keep the study focused on antiviral potency, pharmacokinetics, and the GP-stabilization mechanism.

(10) Review: Line 190 – 195: Given that the authors report the structural data at a later point in the manuscript, they should consider whether it makes sense to move this section down so that their conclusion comes after the presentation of the results supporting it.

Response: We have revised the text so that the mechanistic model is presented after the structural data.

(11) Review: Line 239: Is the use of "western portion/region" and "eastern portion/region" commonly used when talking about protein structures?

Response: "Western" and "eastern" are used here as visual descriptors, consistent with directional nomenclature sometimes used in structure-based drug discovery to describe different regions of ligands. To help readers, we have clarified that these terms refer to the two portions of MWAC-3634 in the orientation shown in Fig. 5C.

(12) Review: Line 246: The authors should justify their decision to use a non-standard mouse-adapted strain with just 1 of 3 GP mutations included. Why did they retain the S246P mutation, but reverted the mutation at position 65 and 544? How does this affect the stringency of their animal model?

Response: We have clarified in the Methods section that the MA EBOV strain used in this study was designed to retain mouse-adaptive mutations while minimizing changes near the MWAC-3634-binding region. GP residues 65 and 544 were reverted to the WT residues because they are located at or close to the MWAC-3634-binding region and could directly affect compound binding. In contrast, GP residue 246 is in a disordered region not mapped in our structural model, and the eight additional mutations are outside GP. Therefore, these retained mutations are not expected to directly affect MWAC-3634 binding to GP.

The goal was to use a suitable mouse-adapted EBOV strain for proof-of-concept in vivo evaluation of MWAC-3634, not to identify an optimal strain or mouse model, which would require a separate systematic study.

The observed disease severity in this model indicates reasonable stringency: the vehicle-control group showed substantial disease severity and mortality, with only 2 of 7 mice surviving.

(13) Review: The authors should comment on the location of S246 with relation to the binding pocket of MWAC-3634. Do they, based on the available structural information (maybe in conjunction with modelling), expect an impact of the S246P mutation on this pocket?

Response: Please see our response to Comment 12. Briefly, GP residue 246 is in a disordered region not mapped in our structural model and is therefore not expected to directly affect the MWAC-3634-binding pocket. Regardless of any potential indirect effect on compound binding, MWAC-3634 still showed strong potency against this MA EBOV strain, although with slightly reduced potency compared with WT EBOV. The MA EBOV strain was therefore suitable for the intended proof-of-concept in vivo evaluation, as the vehicle-control group showed substantial disease severity and mortality.

(14) Review: Line 296: "Our structural study of the EBOV GP ectodomain in complex with MWAC-3634 represents a major breakthrough" – I would recommend the authors leave it up to the readers to make such quality judgements about their work. Alternatively, they would have to provide compelling arguments for why they consider their study to represent such a breakthrough.

Response: We agree that the original wording was unnecessarily subjective. We have removed the phrase "represents a major breakthrough" and revised the sentence to describe the technical outcome more neutrally. The revised text now states: "Our structural study of the EBOV GP ectodomain in complex with MWAC-3634, enabled by the use of an EBOV GP-targeting nanobody to facilitate cryo-EM analysis, provided a high-resolution view of the compound-binding mode."

(15) Review: Line 308: "Our published structures of SUDV and MARV GPs in complex with NPC1 indicate that this pocket is relatively conserved with respect to EBOV. Consistent with this structural conservation, MWAC-3634 retains measurable activity against both SUDV and MARV pseudoviruses, albeit at substantially reduced potency." Given the authors' access to these structural information, can they model MWAC-3634 in complex with SUDV GP or MARV GP, and by doing so provide an explanation for the reduced potency?

Response: We have added an alignment of the MWAC-3634-contacting residues across EBOV, SUDV, and MARV GPs as Fig. S6B. This analysis shows that the GP1-GP2 interface pocket is structurally present in these filoviruses, but the MWAC-3634-contacting residues are only partially conserved. We have revised the Discussion accordingly to explain that sequence and structural variation near the compound-contacting residues likely contributes to the reduced potency against SUDV and MARV pseudoviruses.

(16) Review: Line 313: "suggest that structure-guided adaptation of this scaffold may enable broader-spectrum GP-stabilizing anti-filovirus inhibitors." Given that the current data indicate a rather narrow specificity of MWAC-3634, I would consider this statement rather speculative. Can the authors provide some arguments why they think this pocket or the mechanism of GP stabilization might be amenable to broad spectrum inhibition? The fact that they observe apparent conservation of the pocket when comparing it to SUDV/MARV doesn't really hold, as MWAX-3634 acts comparatively poorly against those viruses. Otherwise I would recommend omitting or rephrasing this statement.

Response: We have revised the statement to tone down speculation and to better reflect the reduced potency against SUDV and MARV. The revised text now states: "Whether future structure-guided optimization of this scaffold can improve cross-filovirus potency will require further study."

(17) Review: Line 335: "our study offers new insights into the mechanisms of EBOV entry and evolution". Please specify what these new insights are, or omit the sentence.

Response: We agree that the original sentence was too broad. We have removed the sentence stating that the study provides new insights into EBOV entry and evolution. The revised paragraph now focuses on the mechanism directly supported by our data, namely that MWAC-3634 stabilizes prefusion GP and thereby blocks the GP conformational transition required for viral entry.

(18) Review: Methodological details appear to be missing, e.g. descriptions of assays to determine microsomal half-life or plasma stability (reported in Fig. 2B).

Response: We have added methodological details for the microsomal half-life and plasma-stability assays to the revised Methods section.

(19) Review: Figure 2A: What do logP and tPSAS stand for? Units (Da, nm²?) appear to be missing.

Response: We have revised Fig. 2A to include the missing units: molecular weight is now reported in daltons, and tPSA is reported in Å². We also revised the Fig. 2 legend to define LogP and tPSA.

(20) Review: Figure 3: GP-ΔM is not defined.

Response: We have changed "GP-ΔM" to "GP" in Figure 3 to avoid ambiguity.

(21) Review: Figure 3: Colours are not explained.

Response: We have revised the Figure 3 legend to explain the colors used in panels A and B. Specifically, red curves represent experimental SPR sensorgrams, and black curves represent fitted binding curves. The colors in panels C and D are already defined within the figure.

Reviewer 2

(1) Review: The main claim of "GP stabilization" relies on DSF thermal shifts and the cryo EM structure. No orthogonal biophysical or functional assay is provided to confirm that MWAC 3634 indeed raises the kinetic barrier to GP2 refolding. Please add at least one independent method to support the stabilization model.

Response: We thank the reviewer for this important comment. Our conclusion that MWAC-3634 stabilizes EBOV GP is based on multiple independent lines of evidence, including high-affinity binding to EBOV GP by SPR, compound-induced thermal stabilization by DSF, and a high-resolution cryo-EM structure showing that MWAC-3634 occupies the GP1–GP2 interface pocket and forms extensive contacts with both GP1 and GP2. Notably, in prior EBOV GP small-molecule studies, compounds binding the same GP1–GP2 pocket were proposed to destabilize GP and reduce the barrier to GP2 refolding primarily based on DSF-measured decreases in GP thermostability.

A similar evidentiary framework has also been widely used for filovirus GP-targeting antibodies and nanobodies. In these studies, stabilization of the prefusion GP conformation has commonly been inferred from structural characterization combined with thermostability assays, and not from direct measurement of GP2-refolding kinetics. Thus, our use of DSF-measured thermal stabilization, together with SPR binding, cryo-EM structural analysis, and viral entry inhibition, is consistent with established approaches for assigning GP-stabilizing mechanisms to filovirus GP-targeting inhibitors.

We also note that assays directly measuring the kinetic barrier for filovirus GP transition from the prefusion to post-fusion state are not currently well established, in part because the precise physiological triggers and intermediate states governing filovirus GP refolding remain incompletely defined. Developing and validating such an assay would require a substantial dedicated effort and is outside the scope of the present study.

Nevertheless, we agree that our current data do not directly measure GP2-refolding kinetics. We have therefore revised the manuscript to clarify this point and to frame the kinetic-barrier interpretation as a model rather than as a directly measured parameter. Specifically, in the Results, we now state that the SPR and thermostability data support a model in which MWAC-3634 stabilizes prefusion GP and blocks EBOV entry. In the Discussion, we further clarify that MWAC-3634 is proposed to raise the barrier to GP conformational transition based on thermostability assays, high-affinity GP binding, cryo-EM structural analysis, and viral entry inhibition.

(2) Review: MWAC 3634 has very low aqueous solubility, high plasma protein binding, and short mouse microsomal half life. Although oral efficacy was observed, these properties are far from desirable. The authors should provide a critical discussion of whether this scaffold can be further optimized or whether the current results mainly serve as a mechanistic probe.

Response: MWAC-3634 shows strong antiviral potency and oral activity, and we agree that further pharmacokinetic optimization will be needed before advancement as a therapeutic candidate. We have revised the Discussion to state: "In addition to its exceptional potency, MWAC-3634 demonstrated favorable pharmacokinetic properties, including high oral bioavailability (69% relative to IV), a reasonable in vivo half-life (2.6 hours after IV dosing), and prolonged plasma stability (889-minute half-life). Notably, total plasma concentrations following oral dosing remained above 2 μM for eight hours, consistent with sustained systemic exposure. Although MWAC-3634 has lower aqueous solubility than toremifene, its high antiviral potency and sustained plasma concentrations after oral dosing in mice support further structure-guided optimization (Table S1). Future analog development will focus on improving solubility and metabolic stability while preserving GP-binding affinity and antiviral potency, guided by the high-resolution GP-MWAC-3634 structure described below. Together, the high potency, lack of detectable cytotoxicity under assay conditions, and encouraging oral exposure support MWAC-3634 as a lead compound for further anti-EBOV therapeutic development." Thus, we view MWAC-3634 as both a mechanistic probe for GP stabilization and a lead scaffold for further development as an anti-EBOV therapeutic candidate.

(3) Review: The comparison with toremifene is qualitative. Please provide a supplementary table listing the buried surface area (BSA) and number of hydrophobic/hydrogen bond contacts for MWAC 3634 versus toremifene from the superimposed structures. This would objectively support the claim that MWAC 3634 engages more extensively with the eastern region of the pocket.

Response: We have added a new supplementary table comparing MWAC-3634 and toremifene as EBOV GP-targeting small-molecule inhibitors. The table includes compound-GP interface area, total compound-contacting GP residues, hydrophobic contacts, polar interactions, and the distribution of contacts in the western and eastern regions of the GP1-GP2 pocket. This comparison provides quantitative support for the conclusion that MWAC-3634 engages the pocket more broadly than toremifene, including more extensive interactions in the eastern region of the pocket.

(4) Review: Throughout the manuscript, units and numbers sometimes lack a space (e.g., "0.65nM").

Response: These have been fixed.

Reviewer 4

(1) Review: Please compare the efficacy and pharmacokinetic properties (including half-life, solubility, and and dosing regimen in mice) of the small-molecule inhibitor MWAC-3634 with those of previously reported small-molecule inhibitors. This comparison will provide a clear and intuitive assessment of the advantages and disadvantages of MWAC-3634.

Response: We have added a new supplementary table comparing MWAC-3634 with toremifene, a representative previously characterized EBOV GP-targeting small-molecule inhibitor. The table compares antiviral potency, GP-binding affinity, compound-GP interface area, compound-contacting GP residues, solubility, plasma protein binding, available pharmacokinetic properties, mouse dosing regimen, and efficacy. This comparison highlights the advantages of MWAC-3634, including higher GP-binding affinity, substantially greater anti-EBOV potency, broader engagement of the GP1-GP2 pocket, and oral activity in the mouse challenge model, while also noting

its current limitations, including low aqueous solubility, high plasma protein binding, and limited mouse microsomal stability.

(2) Review: The authors propose that MWAC-3634 functions by stabilizing GP in its prefusion conformation, representing a novel mechanism distinct from previously reported small-molecule inhibitors. However, during structural determination of the MWAC-3634-GP binary complex, the flexibility of GP made it challenging to unambiguously identify the density corresponding to MWAC-3634. To address this, the authors introduced a nanobody Nanosota-EB2 to stabilize the GP conformation, enabling successful determination of the GP-EB2-MWAC-3634 ternary complex structure where MWAC-3634 density could be clearly resolved. Interestingly, the previously reported structures of GP-destabilizing small molecules were directly determined through binary complex. To further validate the proposed stabilization mechanism of MWAC-3634, it would be valuable for the authors to provide additional experimental evidence demonstrating that MWAC-3634 indeed maintains GP in its prefusion state.s

Response: We have revised the manuscript to clarify the rationale for using Nanosota-EB2 in the cryo-EM analysis. Previous EBOV GP-compound structures were determined by crystallography, where crystal packing and/or crystallization conditions may help stabilize GP and facilitate ligand density visualization. However, the GP-MWAC-3006 complex showed preferred particle orientation on cryo-EM grids, which limited the quality of the reconstruction. To overcome this limitation, we purified a ternary complex comprising recombinant EBOV GP ectodomain, MWAC-3006, and Nanosota-EB2, a nanobody that binds the GP2 subunit of EBOV GP 37,39. The addition of the nanobody improved particle orientation distribution and increased the effective molecular mass of the complex, thereby facilitating higher-quality cryo-EM reconstruction. As a result, the cryo-EM structure of the ternary complex was determined at 2.54 Å resolution (Fig. S8; Table S2). Moreover, the proposed GP-stabilization mechanism is supported by DSF experiments performed in the absence of Nanosota-EB2, which showed that MWAC-3634 increased the thermal stability of recombinant EBOV GP. Thus, Nanosota-EB2 was used to overcome a cryo-EM technical limitation rather than to define the stabilization mechanism, which is supported independently by thermal-stability experiments performed without the nanobody.
